# Human Embryonic Stem Cell-Derived Cardiomyocytes Regenerate the Infarcted Pig Heart but Induce Ventricular Tachyarrhythmias

**DOI:** 10.1016/j.stemcr.2019.04.005

**Published:** 2019-05-02

**Authors:** Rocco Romagnuolo, Hassan Masoudpour, Andreu Porta-Sánchez, Beiping Qiang, Jennifer Barry, Andrew Laskary, Xiuling Qi, Stéphane Massé, Karl Magtibay, Hiroyuki Kawajiri, Jun Wu, Tamilla Valdman Sadikov, Janet Rothberg, Krishna M. Panchalingam, Emily Titus, Ren-Ke Li, Peter W. Zandstra, Graham A. Wright, Kumaraswamy Nanthakumar, Nilesh R. Ghugre, Gordon Keller, Michael A. Laflamme

**Affiliations:** 1 McEwen Stem Cell Institute, University Health Network, Toronto, ON M5G 1L7, Canada; 2 Peter Munk Cardiac Centre, University Health Network, Toronto, ON M5G 2N2, Canada; 3 Schulich Heart Research Program, Sunnybrook Health Sciences Centre, Toronto, ON M4N 3M5, Canada; 4 Toronto General Hospital Research Institute, University Health Network, Toronto, ON M5G 1L7, Canada; 5 Centre for Commercialization of Regenerative Medicine, Toronto, ON M5G 1M1, Canada; 6 University of Toronto, Toronto, ON M5G 1L7, Canada

**Keywords:** human embryonic stem cell-derived cardiomyocytes, pluripotent stem cells, myocardial infarction, ventricular tachyarrhythmias, electroanatomical mapping, MRI

## Abstract

Human embryonic stem cell-derived cardiomyocytes (hESC-CMs) show considerable promise for regenerating injured hearts, and we therefore tested their capacity to stably engraft in a translationally relevant preclinical model, the infarcted pig heart. Transplantation of immature hESC-CMs resulted in substantial myocardial implants within the infarct scar that matured over time, formed vascular networks with the host, and evoked minimal cellular rejection. While arrhythmias were rare in infarcted pigs receiving vehicle alone, hESC-CM recipients experienced frequent monomorphic ventricular tachycardia before reverting back to normal sinus rhythm by 4 weeks post transplantation. Electroanatomical mapping and pacing studies implicated focal mechanisms, rather than macro-reentry, for these graft-related tachyarrhythmias as evidenced by an abnormal centrifugal pattern with earliest electrical activation in histologically confirmed graft tissue. These findings demonstrate the suitability of the pig model for the preclinical development of a hESC-based cardiac therapy and provide new insights into the mechanistic basis of electrical instability following hESC-CM transplantation.

## Highlights

• hESC-CM transplantation partially remuscularizes the infarcted pig heart
• hESC-CM recipients show frequent tachyarrhythmias at early time points
• Graft-related arrhythmias arise from focal mechanisms rather than macro-reentry

## Teaser

In this article, Laflamme and colleagues show that the transplantation of human embryonic stem cell-derived cardiomyocytes (hESC-CMs) partially remuscularizes the scar of infarcted and appropriately immunosuppressed pigs. hESC-CM recipients exhibited frequent monomorphic ventricular tachycardia before reverting back to normal sinus rhythm by 4 weeks post transplantation. These graft-related tachyarrhythmias were found to be due to focal mechanisms rather than macro-reentry.

## Introduction

Following myocardial infarction (MI), necrotic cardiomyocytes are replaced with non-contractile scar tissue, often initiating heart failure. Currently available treatment options for post-MI heart failure include drugs that slow disease progression but do not reverse damage, mechanical circulatory support with complications including thrombosis, infection and the need for an external power supply, and cardiac transplantation limited by the inadequate supply of donor hearts. This situation has driven intense recent interest in the development of alternative cell-based approaches to achieve cardiac repair. The transplantation of various adult stem cell types has been reported to improve left ventricular (LV) contractile function, but beneficial effects appear to be modest and attributable to indirect mechanisms rather than the generation of new cardiomyocytes (Romagnuolo and Laflamme, 2017). By comparison, cardiomyocytes derived from pluripotent stem cells (PSCs) show stable engraftment in multiple MI models, repopulating the infarct scar with electromechanically-integrated new muscle (Caspi et al., 2007, Chong et al., 2014, Laflamme et al., 2007, Shiba et al., 2012, Shiba et al., 2014, Shiba et al., 2016, van Laake et al., 2008). In an initial proof-of-concept study, our group showed that the transplantation of human embryonic stem cell-derived cardiomyocytes (hESC-CMs) in a rat MI model mediates the partial remuscularization of the infarct scar and has beneficial effects on regional and global LV contractile function (Laflamme et al., 2007). Later, we used a guinea pig MI model and a fluorescent graft-autonomous reporter of graft activation to show that hESC-CM grafts are capable of electromechanical integration and synchronous activation with host myocardium during systole (Shiba et al., 2012).

There have also been more recent efforts to test hESC-CMs and related PSC derivatives in large-animal MI models. Primate ESC-derived multipotent cardiovascular progenitors have been shown to differentiate into multiple cardiac lineages including ventricular myocytes following allotransplantation into infarcted non-human primates (Blin et al., 2010). The Murry laboratory described the successful engraftment of committed cardiomyocytes from hESCs in the infarcted hearts of small macaques (Chong et al., 2014). In the latter study, the authors observed an impressive degree of remuscularization following hESC-CM transplantation, as well as histological evidence of graft cardiomyocyte maturation over time. However, hESC-CM recipients exhibited transient, non-lethal ventricular tachyarrhythmias (VTs) that were not observed in infarcted monkeys receiving vehicle alone. More recently, Shiba et al. (2016) described qualitatively similar results following the allotransplantation of primate induced PSC-derived cardiomyocytes (iPSC-CMs) in cynomolgus monkeys.

While the field has learned a tremendous amount from the work in the preceding animal models, we reasoned that efforts to develop and translate a safe, effective PSC-based cell therapy would greatly benefit from additional preclinical testing in the infarcted pig heart. The limitations of rodent MI models are widely recognized, and even the aforementioned transplantation work in non-human primate models involved relatively small species with substantially different cardiac structure and physiology from humans. *Macaca nemestrina* and *Macaca fascicularis* typically have body weights of ∼8 kg and ∼3 kg, respectively; and both species exhibit sinus heart rates that exceed those of humans (*Macaca nemestrina*, ∼120 beats per minute [bpm]; *Macaca fascicularis*, ∼165 bpm; humans, ∼60–100 bpm) (Chong et al., 2014, Kato et al., 2014, Malinow et al., 1977, Shiba et al., 2016). Heart rate is a particularly relevant parameter to consider when evaluating electrophysiological outcomes following cell transplantation because recipient species with rapid rates could affect the electromechanical function of implanted human cardiomyocytes and/or mask graft-related arrhythmias that would otherwise occur in slower-rated humans. By comparison, the pig heart's weight-to-body ratio is nearly identical to that of an adult human (∼5 g/kg) (Hughes, 1986). The pig heart also has a cardiac structure, sinus rate (∼90 bpm), and contractile function that closely resemble that of an adult human (Lelovas et al., 2014). Relative to the primate, the pig also provides significantly greater throughput and reduced experimental costs, and its larger size facilitates better imaging and makes it more amenable to interventions used in adult humans (e.g., catheter-based electroanatomical mapping [EAM]). Given these practical considerations, the pig has been routinely used for the late preclinical testing of novel cardiac interventions.

With this in mind, we hypothesized that hESC-CM transplantation into the infarcted hearts of suitably immunosuppressed pigs would result in their stable engraftment and the partial remuscularization of the infarct scar with outcomes comparable with that seen in other smaller, preclinical models. While this was a feasibility study with primarily histological endpoints, we also examined the functional consequences of hESC-CM transplantation, including LV dimensions and contractile function using cardiac magnetic resonance imaging (MRI), as well as electrophysiological behavior by telemetric electrocardiography (ECG) monitoring and catheter-based EAM and pacing studies.

## Results

### Scaled Production of hESC-CMs

**Figure 1:**
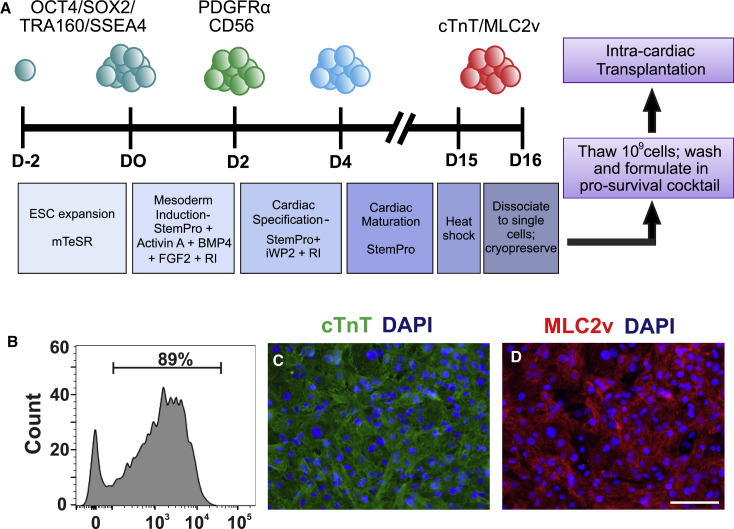
Generation and Phenotype of hESC-CMs for Transplantation Studies (A) Protocol for hESC-CM production in stirred-tank bioreactors. After expansion in the undifferentiated state, hESC aggregates were differentiated in suspension culture using a staged differentiation protocol. Cell populations were routinely monitored by flow cytometry for markers of pluripotency (OCT4, SOX2, TRA160, and SSEA-4), mesodermal progenitors (PDGFRα and CD56), and committed cardiomyocytes (cardiac troponin T [cTnT] and MLC2v). hESC-CM cultures were transiently heat-shocked after 15 days of *in vitro* differentiation, dispersed to single cells on day 16, and cryopreserved. Immediately prior to transplantation, cells were thawed, washed, and resuspended in a pro-survival cocktail. (B) cTnT flow cytometry plot for a representative preparation of ESI-17 hESC-CMs. (C and D) Immunostaining for cTnT (C; green) and MLC2v (D; red) with a nuclear stain (DAPI; blue) on representative fields of ESI-17 hESC-CMs. Scale bar, 100 μm.

To generate the requisite number of hESC-CMs for transplantation studies in the pig heart, we employed a highly scalable stirred-tank bioreactor system to expand undifferentiated hESCs and differentiate them into cardiomyocytes (Prowse et al., 2014). Figure 1A depicts the protocol used to induce cardiac differentiation, which was applied to suspension cultures in either 125-mL or 1-L bioreactors. For an initial pilot transplantation study (n = 2 pigs; animal identification numbers P1 and P4), we generated hESC-CMs using the HES-2 hESC line, which resulted in populations with a mean cardiomyocyte purity of 64.3% ± 17.2% and ventricular myocyte purity of 13.3% ± 5.3%, as determined by flow cytometry for the pan-cardiomyocyte and ventricular markers cardiac troponin T (cTnT) and myosin light chain-2v (MLC2v), respectively. For subsequent transplantation experiments, we switched to the ESI-17 hESC line (Crook et al., 2007), which was originally derived under good manufacturing practice conditions but was here differentiated under research-grade standards. ESI-17 hESC-CM populations had an average cTnT purity of 86.3% ± 0.8% and MLC2v purity of 36.3% ± 5.7% (Table S1). For transplantation studies using cardiomyocytes from both lines (mean cTnT purity of 81.9% ± 3.9%), hESC-CMs were cryopreserved prior to transplantation and then thawed at high viability (80.7% ± 2.4% live cells).

### hESC-CMs Form Stable Grafts in Injured Pig Hearts

Figure S1 depicts the sequence of experimental procedures employed in the pig MI model. In brief, to test the hypothesis that hESC-CMs will stably engraft in the infarcted pig heart, we induced MIs in adult pigs by 90-min occlusion of the mid-left anterior descending (LAD) coronary artery via percutaneous balloon dilation catheter, followed by reperfusion. At 3 weeks post MI, pigs underwent a small left lateral thoracotomy and direct transepicardial delivery of either 1 × 10^9^ hESC-CMs in pro-survival cocktail (n = 7) or vehicle alone (pro-survival cocktail alone; n = 7). To prevent graft cell rejection, we pharmacologically immunosuppressed all animals 5 days prior to transplantation until euthanasia at either 2 or 4 weeks post transplantation. Details regarding the specific cell populations implanted in each animal are listed in Table S1.

**Figure 2:**
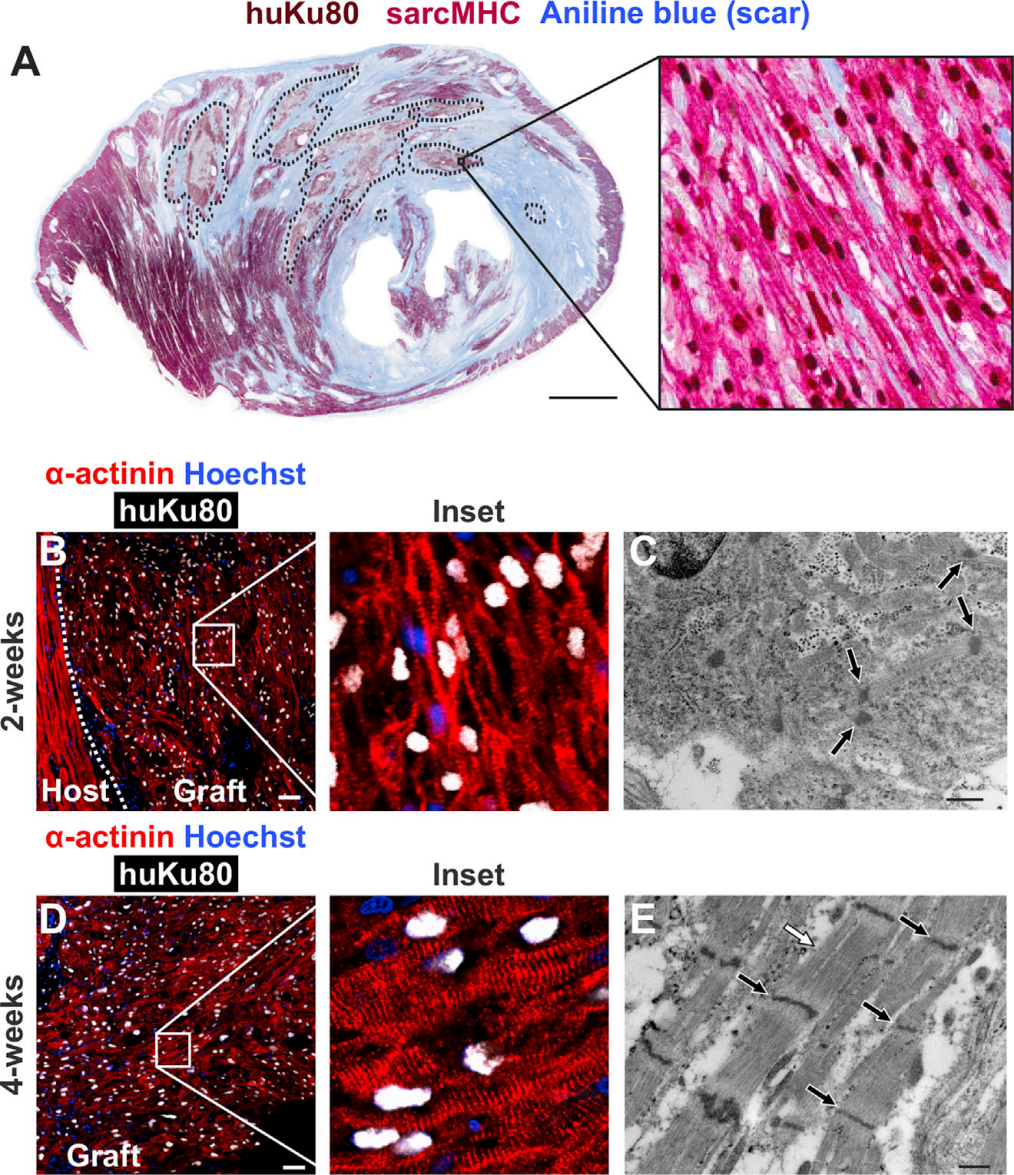
hESC-CMs Partially Remuscularize the Infarct Scar and Mature over Time Engrafted hearts were transversely sectioned from apex to base, then whole-mount sections were analyzed by immunohistochemistry to identify host and graft myocardium and their relationship to the scar. (A) Representative cross-section through hESC-CM recipient heart at 14 days post transplantation showing substantial remuscularization of the infarct scar (blue) by human myocardium (dotted lines). Both human and graft myocardium expressed sarcomeric myosin heavy chain (sarcMHC, red), and grafts cells were identified by immunostaining for human-specific Ku80 (brown nuclei). Scale bar, 5 mm. (B–E) Graft myocardium showed evidence of structural maturation by immunohistochemistry (B and D) and ultrastructure (C and E) with increasing sarcomeric organization and alignment from 2 to 4 weeks post transplantation (B and C versus D and E, respectively). (B and D) White, human-specific Ku80; red, α-actinin; blur, nuclear marker (Hoechst). Scale bar, 50 μm. (C and E) Black arrows, Z-lines; white arrow, M-bands. Scale bar, 0.5 μm.

All recipient hearts were then transversely sectioned at uniform intervals (Figure S1C) and subjected to histomorphometry to assess the extent of the infarct scar, as well as the size, composition, and distribution of the resulting graft. Infarct size (as determined by aniline blue staining on two whole-mount sections taken at 5 and 15 mm from the apex) was comparable in both cell recipients and vehicle controls (34.0% ± 5.8% versus 25.6% ± 6.6% of LV area at 4 weeks post MI, respectively; p = 0.35). hESC-CM recipients showed graft myocardium that occupied a mean of 15.2% ± 3.4% of the scar area (Table S1). hESC-CM graft was composed of irregularly contoured islands of myocardial tissue distributed widely throughout the scar that immunostained with the cardiac marker sarcomeric myosin heavy chain (sarcMHC) and the human-specific nuclear marker Ku80 (Figures 2A and S2). Individual graft implants ranged from a few mm^2^ to >90 mm^2^ in size. A comparison of hESC-CM graft structure at 2 versus 4 weeks post transplantation indicates that grafts at the later time point have greater sarcomeric content, organization, and alignment as determined by α-actinin staining (Figures 2B and 2D) and electron microscopy (Figures 2C and 2E). These findings are consistent with the *in vivo* graft maturation noted in prior transplantation work in smaller recipient species (Chong et al., 2014, Laflamme et al., 2005).

**Figure 3:**
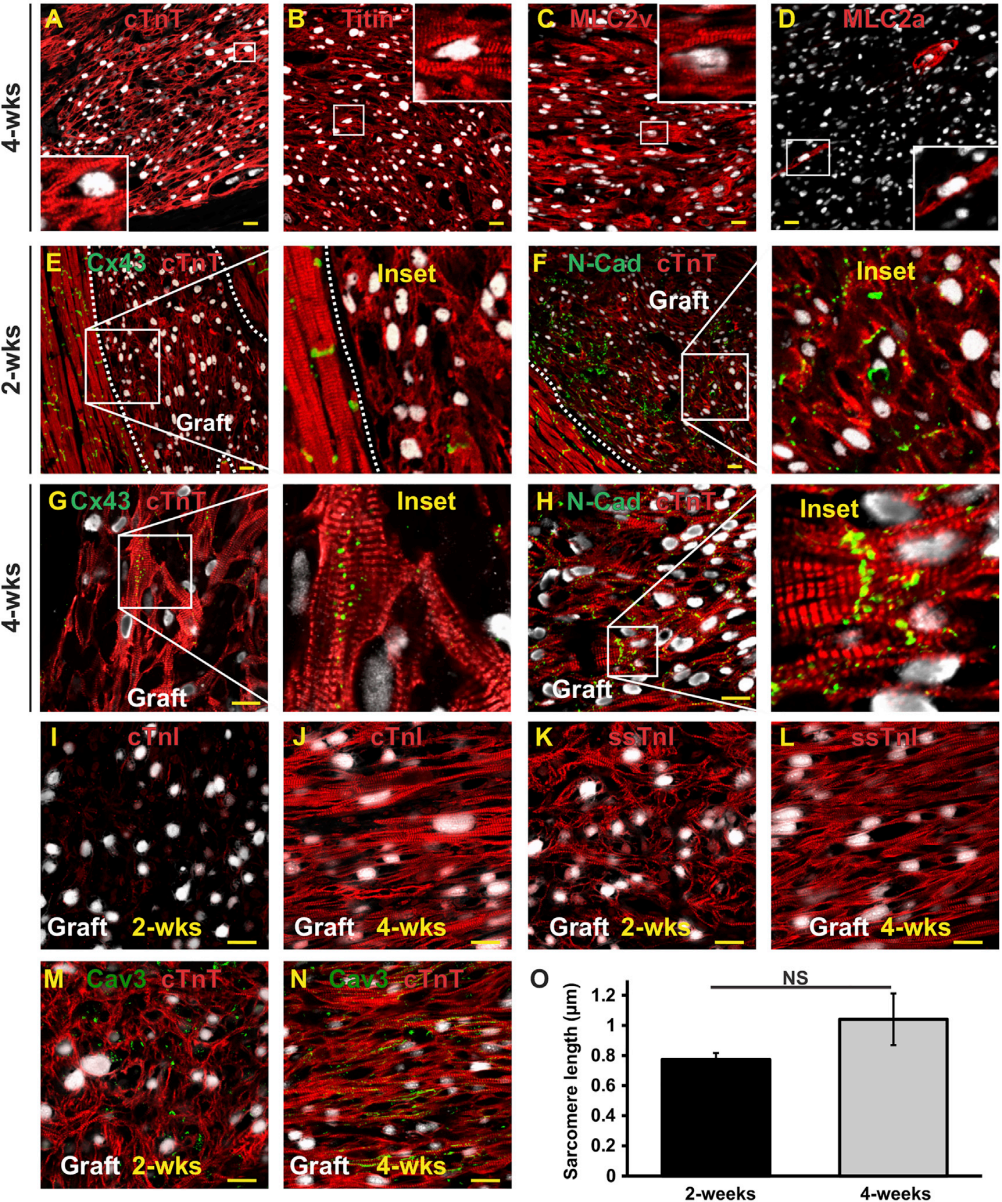
hESC-CM Graft Tissue Expresses Expected Cardiac Markers (A–N) Confocal immunofluorescence of engrafted hearts at either 4 weeks (A–D, G–J, L, and N) or 2 weeks (E, F, I, K, and M) post transplantation. In all images, graft cells were identified by dual-labeling for human-specific Ku80 (white nuclei). (A–D) hESC-CM graft myocardium was uniformly positive for cardiac troponin T (cTnT; A) and titin (B), largely positive for MLC2v (C), and included only scattered MLC2a-positive cells (D). (E–G) Graft myocardium exhibited patchy and low-level expression of the gap junction protein connexin-43 (Cx43, green), but expressed abundant N-cadherin (N-Cad, green) (E and F at 2 weeks and G and H at 4 weeks). (I–N) Graft myocardium showed evidence of structural maturation over time (2 versus 4 weeks post transplantation) as identified by better organized sarcomeres and enhanced expression of maturation markers including cardiac troponin I (cTnI; I and J), slow-skeletal troponin I (ssTnI; K and L), and the T-tubule-associated protein caveolin-3 (Cav3; M and N). Scale bars in all images, 20 μm. (O) Comparison of sarcomere length from graft at 2 or 4 weeks post transplantation (n = 3 pigs per time point).

The immunophenotype of graft tissue (from cardiomyocytes derived from HES-2 and ESI-17 lines) was examined using a wide variety of cardiac and non-cardiac markers. In addition to expressing sarcMHC and α-actinin, hESC-CM graft tissue immunostained strongly for expected sarcomeric markers including cTnT, titin, MLC2v, and MLC2a (Figures 3A–3D). Interestingly, although our cardiac differentiation protocol was expected to yield an admixture of cardiac subtypes (Protze et al., 2016) and only approximately one-third of cells expressed ML2Cv prior to transplantation (Table S1), the vast majority of the surviving graft cardiomyocytes were MLC2v positive (>90%), and only rarely scattered individual MLC2a-positive graft cells were identified (Figures 3C and 3D). Consistent with prior transplantation studies in other species (Chong et al., 2014, Laflamme et al., 2005), we found that hESC-CM graft tissue at both the 2- and 4-week time points expressed relatively low levels of the cardiac gap junction protein connexin-43 (Cx43). Cx43 immunoreactivity was largely limited to occasional areas at the periphery of the graft or near points of host-graft contact in the border zone (Figures 3E and 3G). By contrast, hESC-CM grafts at both time points strongly expressed the myocardial adherens junction protein N-cadherin (Figures 3F and 3H). We found additional histological evidence of graft maturation in 4-week-old versus 2-week-old graft tissue, with the former demonstrating increased expression of cardiac troponin I, stronger expression and more efficient sarcomeric organization of slow-skeletal troponin I (ssTnI), and increased expression and enhanced subcellular localization of the T-tubule-associated protein caveolin-3 (Figures 3I–3N). There was also a trend toward increased sarcomere length, although this did not reach statistical significance (Figure 3O).

**Figure 4:**
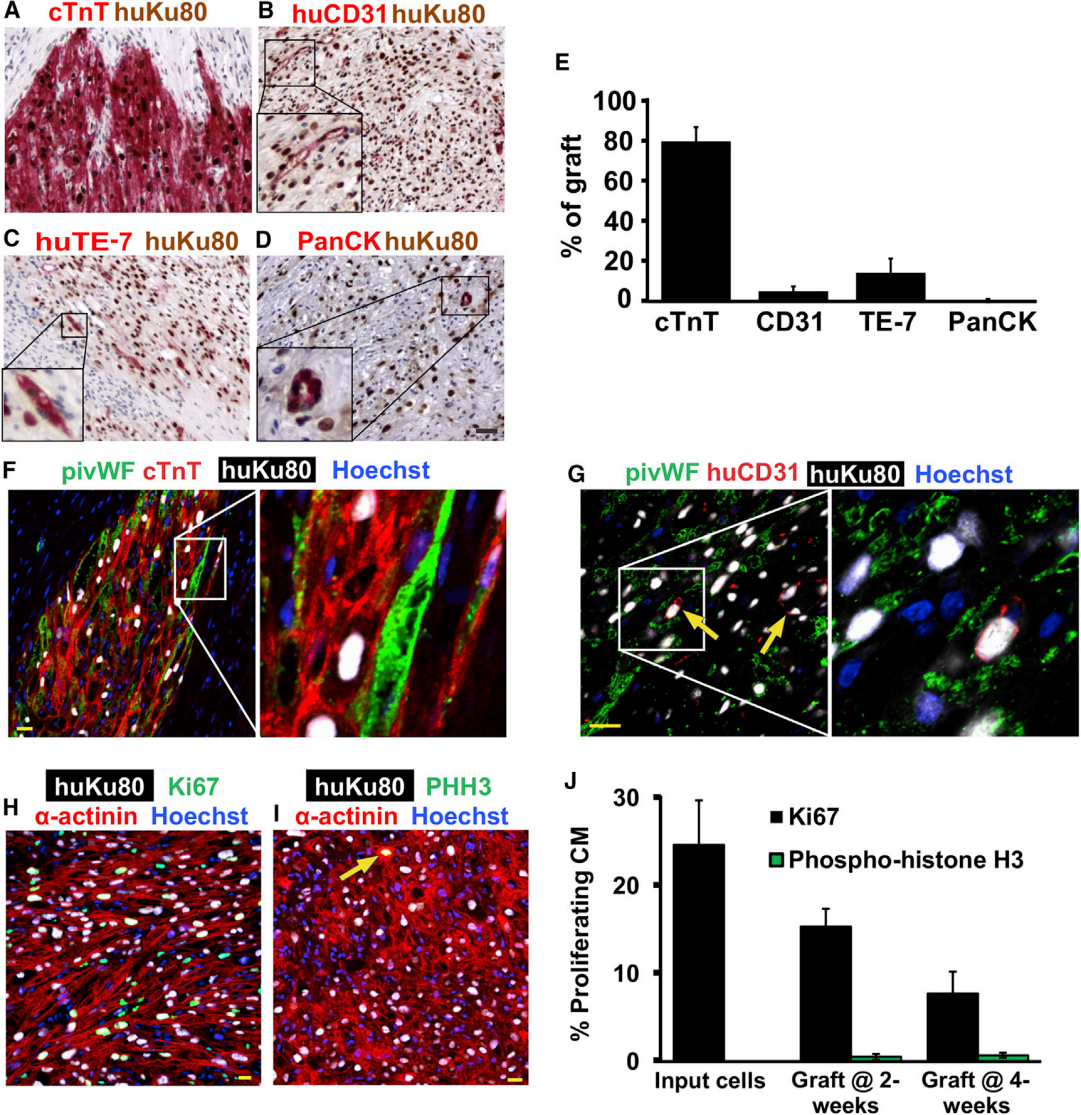
Composition and Proliferation of hESC-CM Grafts (A–D) To evaluate the cardiomyocyte purity and non-myocyte composition of the grafts, histological sections were dual-labeled for human-specific Ku80 (brown nuclei) and cell-type-specific markers (red) including cardiomyocytes (cTnT; A), human-specific endothelial cells (CD31; B), human-specific fibroblasts (TE-7; C), and epithelial cells (PanCK, pan-cytokeratin; D). Scale bar, 50 μm. (E) Fraction of Ku80-positive human graft cells immunostaining positively for each of the preceding cell-type-specific markers. n = 6. (F and G) Host-derived microvessels within the myocardial grafts were identified by confocal immunofluorescence for pig-specific von Willebrand factor (pivWF; green), human-specific Ku80 (white), and (F) cTnT (red) or (G) human-specific CD31 (huCD31; red). Nuclear marker, Hoechst (blue). Arrow indicates a CD31-positive human endothelial cell. Scale bar, 20 μm. (H–J) For determination of proliferation of graft cells, histological sections were triple-labeled for human-specific Ku80 (white), α-actinin (red), or cTnT (red) and either Ki-67 (H; green) or phospho-histone H3 (PHH3; green) (I). Arrow represents a cTnT, Ku80, and PHH3 triple-positive cell. Scale bar, 20 μm. (J) shows the percentage of graft cardiomyocytes that are either Ki-67^+^ or PHH3^+^ at the time of transplantation and at 2 or 4 weeks post transplantation. n = 3 for input cells and n = 6 for graft at 2 and 4 weeks.

No teratomas were identified in hESC-CM recipients by routine stains (H&E), and the graft cells were uniformly negative for tested endodermal (α-fetoprotein^+^) or neuronal (β−tubulin III^+^) markers (Figures S3A and S3B). Grafts were probed with other cell-type-specific antibodies to detect the presence of various non-cardiac elements. In brief, greater than 80% of the human nuclei could be accounted for by the cardiac marker cTnT, but small fractions of endothelial (5.1% ± 2.4% CD31^+^), fibroblastic (14.3% ± 7.1% TE-7^+^), and epithelial (0.5% ± 0.2% pan-cytokeratin^+^) graft cells (5.1% ± 2.4%) were identified (Figures 4A–4E; from n = 6 engrafted hearts analyzed). Moreover, if the two initial animals receiving HES-2-derived populations of lower cardiomyocyte purity are excluded from the analysis, graft composition in the remaining ESI-17 recipients (n = 4) improves to 91.1% ± 1.8% cardiomyocytes, 3.1% ± 1.0% fibroblasts, 0.16% ± 0.12% epithelial cells, and 1.6% ± 0.9% endothelial cells. Importantly, we found no obvious correlation between input cardiomyocyte purity and either graft or infarct size by histomorphometry (R^2^ values of 0.59 and 0.01, respectively; data not shown).

Because the limited quantity of graft capillary endothelium identified by human anti-CD31 immunohistochemistry seemed unlikely to support these large graft implants, we also immunostained engrafted hearts with an antibody against porcine endothelial cells (pig-specific anti-von Willebrand factor [pivWF]). This immunostaining confirmed a high density of porcine microvessels within the graft (Figures 4F and 4G), indicating a brisk host-derived angiogenic response that likely promotes graft survival and maturation. Of note, the employed anti-piVWF antibody does not cross-react with human cells as evidenced by the lack of overlapping staining seen in histological sections triple-stained with anti-piVWF, a second species-specific antibody against human CD31, and anti-human Ku80 (Figure 4G).

To evaluate the proliferative capacity of hESC-CM graft tissue (from cardiomyocytes derived from HES-2 and ESI-17 lines), we performed dual immunolabeling with human-specific Ku80 and either the pan-cell-cycle marker Ki-67 (Figure 4H) or the mitosis marker phospho-histone H3 (PHH3, Figure 4I). We observed a readily detectable but declining fraction of Ki-67^+^ graft cardiomyocytes at all time points (24.7% at the time of transplantation, 15.4% at 2 weeks, and 7.8% at 4 weeks post transplantation), as well as rare individual PHH3^+^ graft cardiomyocytes (Figure 4J). The fraction of Ki-67^+^ non-myocytes at 4 weeks post transplantation was found to be 6.0% ± 0.8%. To screen for graft cell apoptosis, we also routinely stained sections with an antibody against active caspase-3, but found only vanishingly rare positive cells (<0.1%), suggesting that ongoing graft cell death is not greatly limiting (data not shown).

Finally, we screened for evidence of cellular immune rejection in the hearts of hESC-CM recipients by performing immunohistochemistry with porcine-specific antibodies against pan-leukocyte (anti-CD45), T-lymphocyte (anti-CD3), and B-lymphocyte (anti-CD20) markers. In summary, most graft areas showed either no reaction or sparse infiltration at the graft periphery by host mononuclear cells that were composed almost exclusively of CD3^+^ T lymphocytes (Figures S3C–S3J). If this immunostaining pattern was graded using criteria applied to human heart allografts (Stewart et al., 2005), it would be best classified as mild cellular rejection, implying adequate immunosuppression with no change in treatment required.

### LV Structure and Contractile Function in hESC-CM Recipients by MRI

**Figure 5:**
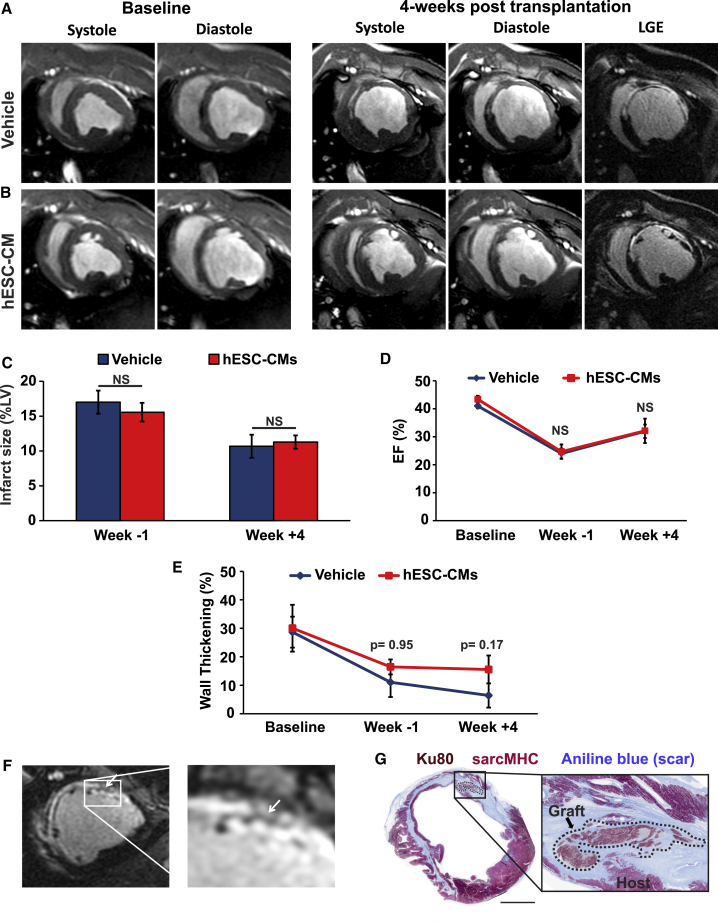
Infarct Size, LV Contractile Function, and Graft Geometry by Cardiac MRI in hESC-CM versus Vehicle Recipients Infarcted pigs receiving either hESC-CMs or vehicle underwent MRI scans at baseline (prior to MI), 1 week prior to transplantation (2 weeks post MI), and 4 weeks post transplantation (7 weeks post MI). (A and B) Short-axis slices from representative vehicle (A) or hESC-CM (B) recipients acquired during systole and diastole at either baseline or 4 weeks post transplantation. Rightmost panels also show LGE-MR images to highlight hyperenhanced scar tissue. (C) Infarct size by LGE-MRI in infarcted pigs receiving hESC-CMs versus vehicle. (D) LVEF in infarcted pigs receiving hESC-CMs versus vehicle. (E) Regional wall thickening in infarcted pigs receiving hESC-CMs versus vehicle. The number (n) for (C) to (E) can be found in Table S2. (F) LGE-MRI images showing strands of non-enhancing tissue identified within the hyperenhanced infarct scar of a representative hESC-CM recipient at 4 weeks post transplantation. (G) Histological section corresponding to the MR images depicted in (F). Sections were stained to identify sarcMHC (red), human-specific Ku80 (brown), and scar (blue), confirming the presence of human myocardial implants with a geometry matching the strands identified by LGE-MRI. Scale bar, 1 mm.

While this was a feasibility experiment that was neither powered nor designed to demonstrate statistically robust salutary effects on contractile function (especially given relatively small infarcts expected to produce only modest reduction in left ventricular ejection fraction [LVEF]), we nonetheless evaluated infarcted hESC-CM and vehicle recipients by serial myocardial late gadolinium enhancement (LGE) MRI. MRI scans were performed at baseline, 2 weeks post MI, and 4 weeks post transplantation (corresponding to 7 weeks post MI) (see Figures 5A and 5B for representative images). Table S2 details the MRI outcomes by individual animals. As expected, infarct scar size was comparable in both experimental groups prior to cell transplantation (15.6% ± 1.3% in hESC-CM versus 17.0% ± 1.7% in vehicle recipients at 2 weeks post MI; p = 0.51), and both groups demonstrated similar reductions in scar size over time (11.3% ± 1.0% in hESC-CM versus 10.7% ± 1.7% in vehicle recipients at 4 weeks post transplantation; p = 0.77) (Figure 5C). There was no evidence of an effect of cell transplantation on LV dimensions or global contractile function, and we found no significant differences in LVEF, LV systolic volume, or LV diastolic volume between hESC-CM and vehicle recipients at the 4-week time point or by pairwise comparisons from −1 to +4 weeks post transplantation (Figures 5D and S4A–S4D). On the other hand, while regional wall thickening in the infarcted segment was similar in both experimental groups prior to transplantation (p = 0.95), there was a suggestion of improved *regional* myocardial contraction in hESC-CM recipients, as evidenced by a non-significant (p = 0.17) increase in dynamic wall thickening of the hESC-CM implanted segment versus vehicle (Figure 5E).

Given the centimeter-scale grafts detected by histology, we hypothesized that larger hESC-CM implants would be visible within the hyperenhanced scar tissue by LGE-MRI. To test this, we used 3D renderings and anatomic landmarks to correlate short-axis LGE-MRI images with whole-mount, transverse histological sections of the engrafted left ventricle taken at the corresponding level. By this approach, we identified numerous instances in which there were islands of non-enhancing tissue with a unique geometry located within the infarct scar that nicely matched with the histologically confirmed graft (Figures 5F and 5G). While there is always some degree of heterogeneity, we did not find similar large non-enhancing regions in infarcted hearts with vehicle alone.

### hESC-CM Recipients Exhibit Abundant VT

**Figure 6:**
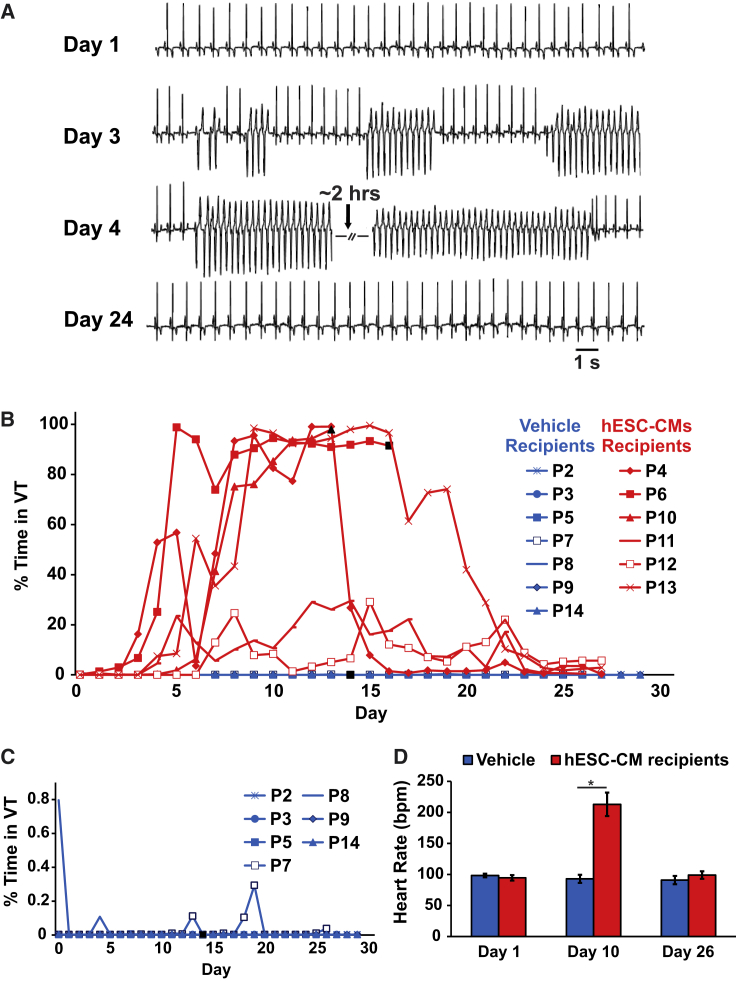
hESC-CM Transplantation Results in Ventricular Tachyarrhythmias Infarcted animals receiving either hESC-CM or vehicle were continuously monitored by telemetric ECG for spontaneous arrhythmias. (A) Representative ECG traces from a hESC-CM recipient acquired on days 1, 3, 4, and 24 post transplantation. (B) Plot depicting the fraction of each day spent in VT in vehicle (blue; n = 7) and hESC-CM (red; n = 6) recipients by time point post transplantation. Pigs that either died or were sacrificed prematurely (P5, P6, and P10) are indicated with a black terminal symbol. (C) By comparison, vehicle recipients showed either no VTs or rare episodes lasting only a few seconds in duration, so a different ordinate axis is required to visualize VT incidence in these animals. (D) Mean heart rate in hESC-CM versus vehicle recipients on days 1, 10, and 26 post transplantation. n = 3 for vehicle and hESC-CM recipients. ^∗^p < 0.01.

Infarcted pigs receiving hESC-CMs (n = 6) or vehicle (n = 7) were continuously monitored by telemetric ECG for spontaneous arrhythmias from the time of thoracotomy until euthanasia. One hESC-CM recipient (P1) was not fitted with a telemetric device but was confirmed to be in sustained VT at the time of euthanasia (2 weeks post thoracotomy). hESC-CM recipients were all initially in normal sinus rhythm, but this group was distinguished by frequent bouts of monomorphic VT that emerged approximately 3–4 days after transplantation and peaked in incidence at approximately 10 days post transplantation (Figures 6A and 6B). By comparison, infarcted pigs receiving vehicle alone showed remarkable electrical stability with rare bouts of VT detected in only 3 of 7 animals, and these events lasted only a few seconds in duration (Figure 6C). During VT, hESC-CM recipients typically showed heart rates in excess of 220 bpm (Figure 6D), and individual VT episodes ranged in duration from a few seconds to >1 h. Indeed, during the window of peak incidence, 4 of 6 hESC-CM recipients were observed to spend an excess of 80% of each day in near-continuous VT. Consistent with prior reports of transient arrhythmogenesis following the transplantation of PSC-derived cardiomyocytes in small monkeys (Chong et al., 2014, Shiba et al., 2016), the incidence of these graft-related arrhythmias gradually decreased over time, such that all hESC-CM recipients returned to normal sinus rhythm by 4 weeks post transplantation. Two hESC-CM recipients (animals P11 and P12) exhibited somewhat less frequent arrhythmias throughout (with a peak VT incidence of ∼30% of each 24-h recording period at 2 weeks post transplantation). One of these animals (P11) had the smallest amount of hESC-CM graft by histomorphometry, but the other (P12) had one of the largest grafts (Table S1). Although limited by the relatively small number of animals enrolled, we found no definite correlation between arrhythmia burden (i.e., total number of hours spent in VT) and either graft size (R^2^ < 0.01) or cardiomyocyte purity (R^2^ = 0.03) (data not shown).

While animals with the preceding graft-related arrhythmias were generally stable from a clinical veterinary standpoint despite no administration of anti-arrhythmic agents or defibrillation, 2 of 7 hESC-CM recipients were lost during the arrhythmogenic period (see Table S2). Cell recipient P6 died on day 16 with sustained VT that degenerated into ventricular fibrillation, while P10 was euthanized on day 13 as per veterinary advice due to complications of heart failure (large infarct) and sustained VT. By comparison, 1 of 7 vehicle recipients (P5) had to be prematurely euthanized due to decompensated heart failure at day 14 (an outcome similar to that of cell recipient P10, which had a comparably sized infarct).

### VT in hESC-CM Recipients Driven by Focal Mechanism

To investigate the mechanistic basis of these graft-related tachyarrhythmias, we performed catheter-based EAM and standard clinical pacing studies in infarcted pigs 10 days after intracardiac transplantation of hESC-CMs (n = 3) or vehicle (n = 4 animals). This 10-day time point was selected because it corresponded to the peak incidence of VT during the preceding telemetric ECG studies (Figure 6B). Consistent with our previous observations, infarcted vehicle recipients did not show spontaneous VT during EAM, nor were they induced to sustained monomorphic VT by challenge with programmed ventricular stimulation. While resistant to VT induction, vehicle recipients could be induced to ventricular fibrillation during programmed ventricular stimulation with coupling of three extra stimuli at <200 ms cycle length (CL), after which they could be restored back to sinus rhythm by direct cardioversion. Bipolar high-density endocardial substrate maps acquired during sinus rhythm confirmed the expected area of low voltage (<1.5 mV) corresponding to the apical LV infarct zone, and endocardial local activation time (LAT) maps from these hearts showed the expected pattern of late activation occurring in the infarct area.

**Figure 7:**
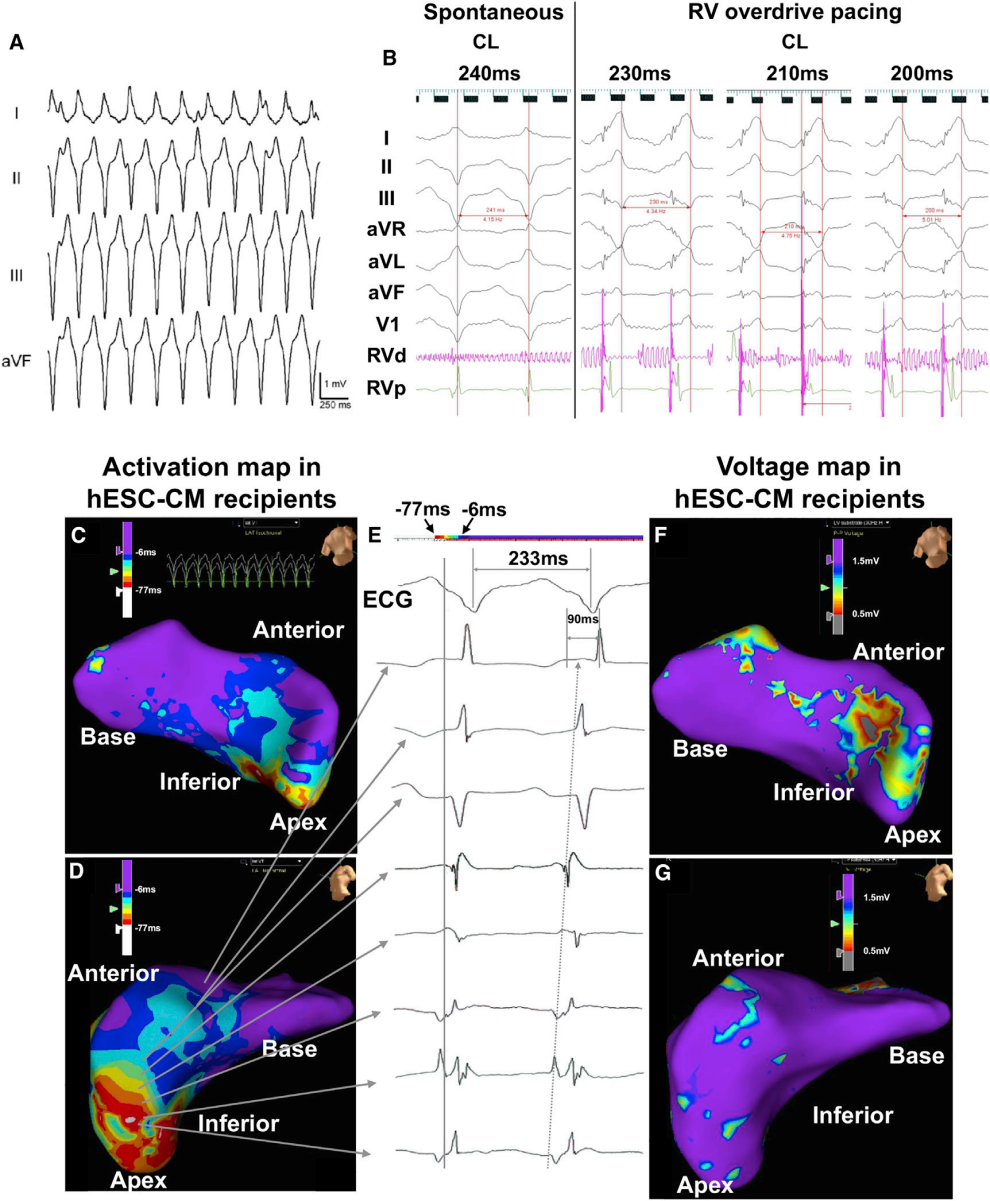
Electrophysiological Studies in hESC-CM Recipients during VT Infarcted pigs underwent EAM and standard clinical electrophysiological studies 10 days after delivery of vehicle or hESC-CMs. (A) Surface ECG recordings from a representative hESC-CM recipient in sustained monomorphic VT with a rate of 257 bpm and a superior axis, consistent with an origin in the apical LV. (B) Surface ECG traces acquired from a representative hESC-CM recipient during overdrive pacing from the right ventricular apex at varying pacing CLs. Note that the QRS complex of the paced beats always shows a fully paced morphology despite pacing at different CL with no evidence of progressive fusion or entrainment. After cessation of pacing maneuvers, the tachycardia continued with variable post-pacing intervals (not shown). (C and D) Septal (C) and anterolateral apical (D) views of an LV endocardial LAT map acquired from an infarcted hESC-CM recipient (P19) during VT displaying focal activation pattern with no evidence of macro-reentry. Areas of earliest activation are marked by white and red, while later-activating areas are depicted in a gradient from orange to yellow to green to blue. (E) Corresponding bipolar electrogram recordings and their respective location on the LV endocardial map of (D). The surface QRS (lead aVF) was used as the timing reference. Note that these local electrograms indicate a relatively wide area of early activation, suggesting a mid-myocardial origin in the apical LV. (F and G) Septal (F) and lateral (G) views of the corresponding LV endocardial voltage map with normal voltage being defined as >1.5 mV, abnormal as 1.5–0.5 mV, and dense scar as <0.5 mV. Areas of low voltage and localized scar are seen in the anterior apical septum. Note that the anterolateral endocardium shows predominantly normal voltages, indicating that the earliest area of VT activation (origin, D) is located in a region with preserved voltages.

By contrast, all three infarcted hESC-CM recipients were already in spontaneous VT at the time of electrophysiological study, although they showed good hemodynamic tolerance throughout the procedure. Two of these animals had sustained monomorphic VT, with P19 exhibiting a superior axis and a rate of 257 bpm (Figure 7A) and P20 exhibiting a superior axis and variable rate of 210–230 bpm. In both of these animals, endocardial LAT maps acquired during VT revealed a focal activation pattern with the site of earliest activation located in the apical LV and occurring approximately 20 ms ahead of the surface ECG (Figures 7C–7E). There was no evidence of macro-reentry either under spontaneous conditions or during entrainment and overdrive pacing protocols. In addition, a voltage map acquired during VT showed a limited area of low voltage located in the inferior apex of the LV (Figures 7F and 7G). Overdrive pacing at varying CLs close to the tachycardia CL resulted in transient overdrive suppression of the VT with manifest fusion and no evidence of entrainment or resetting (Figure 7B). These outcomes are also considered consistent with focal mechanisms rather than macro-reentry.

A third hESC-CM recipient (P21) was found to be in sustained monomorphic VT with an inferior axis and a rate of 261 bpm (rhythm VT1) at the beginning of electrophysiological study, but later spontaneously changed to monomorphic VT with a superior axis and a rate of 242 bpm (rhythm VT2) during the procedure (Figure S5A). The VT1 pattern predominated, and VT2 was only observed transiently. Endocardial LAT maps for VT1 showed a pattern of focal activation originating in the anterior septum at a site later confirmed by correlation of the EAM and 3D LGE-MRI datasets to correspond to the most cranial aspect of the infarct scar (Figure S5B). In this case, pacing resulted in overdrive suppression of the VT1 rhythm with the appearance of a sinus beat, followed by the quick reappearance of VT1 after the cessation of pacing. Multiple pacing maneuvers during either VT1 or VT2 only transiently reverted to sinus rhythm followed by the reappearance of VT1 or VT2 soon after.

While there was no evidence of macro-reentry in the preceding endocardial LAT maps, two infarcted hESC-CM recipients (P20 and P21) were also subjected to epicardial mapping to exclude the possibility of an epicardial reentrant circuit. Again we found no evidence of reentry, but rather only focal activation patterns. Figure S5 demonstrates spontaneous changes in VT morphology and abrupt variations in CL, suggestive of focal automatic behavior and not supportive of a macro-reentrant VT circuit.

For careful correlation of the outcomes of these electrophysiological studies with tissue structure, each heart was harvested at the completion of the experiment, transversely sliced at 5-mm intervals, and used to prepare whole-mount histological sections, which were stained for sarcMHC, human-specific Ku80, and scar tissue. We then used the LGE-MRI dataset and anatomic landmarks to carefully register the 3D EAM with histology with an accuracy of <5 mm. In all three hearts examined, we identified hESC-CM graft tissue at the site of earliest activation in the LAT maps (Figure S6). To further corroborate this correlation, in 2 of 3 hESC-CM recipients (P20 and P21) we delivered radiofrequency ablation lesions in a manner that allowed us to precisely triangulate to the site of earliest activation by histology (including an epicardial ablation mark adjacent to the area of earliest activation and two endocardial marks at a known distance lateral to it). Here again, we unambiguously confirmed that there was hESC-CM graft tissue precisely at the site of earliest activation (Figure S6). Taken collectively, these findings are consistent with graft-related arrhythmias arising via focal mechanisms at the interface between hESC-CM and host myocardium, with no evidence of a macroscopic reentrant circuit.

## Discussion

This study was intended to test the feasibility of intracardiac hESC-CM transplantation in the pig MI model as a critical next step in the preclinical development of these cells. In this work, we successfully demonstrated the stable engraftment of hESC-CMs within the infarct scar of immunosuppressed pigs with minimal cellular rejection and no evidence of teratomas. While we obtained a large degree of remuscularization with surviving myocardial implants approximating a cubic centimeter in volume, porcine hESC-CM recipients exhibited frequent graft-related tachyarrhythmias that were similar in nature but qualitatively worse than those previously reported following the transplantation of PSC-CMs in smaller, faster-rated monkeys (Chong et al., 2014, Shiba et al., 2016). Next, we took advantage of the larger size of the pig heart to perform comprehensive catheter-based electrophysiological investigations and found that the graft-related arrhythmias in hESC-CM recipients arise from focal mechanisms originating at the site of cell implantation, rather than macro-reentry.

Overall, these outcomes in the pig model nicely complement prior work in the field testing hPSC-CMs in rodent and large-animal MI models. In early work, our group and others demonstrated that hESC-CMs can partially remuscularize the infarct scar and mediate improvements in regional and global LV contractile function in infarcted rat and guinea pig hearts (Laflamme et al., 2007, Shiba et al., 2012, van Laake et al., 2008). More recently, the Murry group reported that the transplantation of 0.75–1 × 10^9^ hESC-CMs in infarcted macaque hearts resulted in stable myocardial grafts that occupied ∼10%–40% of the infarct scar and mediated beneficial effects on LVEF (Chong et al., 2014, Liu et al., 2018). Shiba et al. (2016) transplanted 4 × 10^8^ primate iPSC-CMs in infarcted cynomolgus monkey hearts and remuscularized ∼16% of the infarct scar with small improvements in LVEF.

Given that the pig heart is approximately 7- to 8-fold larger than that of these non-human primate hearts, it follows that either a larger cell dose or improved graft survival may be required. Here, we transplanted 1 × 10^9^ hESC-CMs in infarcted porcine hearts and formed grafts that occupied ∼15% of the infarct scar. While a full dose-response study is needed to define the optimal quantity of hESC-CMs in the human-sized pig heart, our findings suggest that cell retention and the absolute volume of surviving myocardial graft in this species is at least comparable with that reported in small monkeys. Prior to commencing this study, we were aware of unpublished reports by multiple investigators indicating that they were unable to obtain stable engraftment after injection of human cardiomyocytes into the pig heart. We speculate that our better outcomes may reflect our comprehensive and strictly monitored immunosuppression regimen (comprising a calcineurin inhibitor, T cell costimulatory blockade, and corticosteroid) and/or use of other interventions previously shown to enhance cardiomyocyte engraftment in rodents (heat-shock of hESC-CMs prior to cryopreservation and their delivery within a pro-survival cocktail) (Laflamme et al., 2007).

Another important outcome in this study was the observation of frequent VTs in infarcted hESC-CM recipients. Interestingly, while graft-related tachyarrhythmias in the pigs were more frequent and more lethal than those reported following PSC-derived cardiomyocyte transplantation in small, faster-rated monkeys (Chong et al., 2014, Shiba et al., 2016), they showed a similar time course. In the present study, VTs first appeared in hESC-CM recipients at a few days after transplantation and peaked in incidence at approximately 10 days post transplantation, with many animals spending >90% of each day in near-continuous VT. VT incidence gradually dissipated over time, such that all animals were in normal sinus rhythm by 28 days post transplantation. Given that a very similar time course for graft-related arrhythmias has now been observed following PSC-CM transplantation in multiple independent models (Chong et al., 2014, Liu et al., 2018, Shiba et al., 2016), this seems like a robust phenomenon and a definite hurdle to successful translation.

Interestingly, while limited by the number of animals enrolled, we detected no obvious correlation between graft size and arrhythmia vulnerability. One would intuitively expect there to be some threshold in graft size below which graft-related arrhythmias would not occur, but a full dose-response study will be required to determine the detailed relationship between graft size and arrhythmia risk. We did not observe any obvious correlation between the input cardiomyocyte purity and arrhythmia incidence, nor was there a correlation between cardiomyocyte purity and either graft or infarct size, suggesting that the presence of non-myocytes did not greatly affect these outcomes.

Given our finding of frequent graft-related arrhythmias in porcine hESC-CM recipients, we used EAM and pacing studies to investigate the mechanistic basis of graft-related VT. Prior to this work, we had hypothesized that reentry would be the predominant mechanism. VTs in post-MI hearts are typically driven by reentry (Benito and Josephson, 2012), and in addition immature hESC-CM graft islands with low graft Cx43 expression and slow conduction velocity (Shiba et al., 2012) seem likely to favor the reentrant phenomenon. In contrast to these assumptions, our findings by telemetric ECG monitoring, EAM, and pacing studies all point toward a focal automatic mechanism rather than reentry. While we cannot definitively exclude micro-reentry (i.e., reentrant phenomenon below the spatial resolution of the available mapping approaches), we found no evidence for a macroscopic reentry circuit by endocardial or epicardial EAM in hESC-CM recipients. By telemetric ECG, VT during initiation had the same QRS morphology as subsequent beats, and we typically observed short bouts of self-terminating VTs that preceded long-lasting VTs. Pacing maneuvers from the right ventricle resulted in overdrive suppression of VT with no evidence of fixed fusion, resetting, or entrainment. Taken collectively, this body of electrophysiological data strongly implicates a focal mechanism rather than reentry, a conclusion that was independently reached by Liu et al. (2018) in the macaque model while this paper was under revision.

If correct, this finding of a focal mechanism raises the intriguing possibility that graft-related arrhythmias may be a graft cell-autonomous phenomenon that can be attenuated by enhancing the electrophysiological phenotype of the input cardiomyocytes. In the present study, the transplanted cell populations were relatively immature and composed of only ∼30% MLC2v^+^ ventricular cardiomyocytes. Interestingly, the surviving graft cardiomyocytes at 2 and 4 weeks post transplantation demonstrated >90% MLC2v^+^ myocytes, an outcome qualitatively similar to that reported in other models (Chong et al., 2014). While this change in immunophenotype post transplantation may reflect preferential retention and/or survival of MLC2v^+^ cells, it seems more likely that initially MLC2v-null cardiomyocytes later go on to express this more mature ventricular marker, given the improvements in other maturation parameters (e.g., ultrastructure, expression of cTnI and caveolin-3) over this same interval. We speculate that this *in vivo* ventricular maturation may also account for the greatly reduced incidence of graft-related arrhythmias at >3 weeks post transplantation.

In future work, we plan to use the pig MI model to test the hypothesis that the transplantation of an initially more homogeneous population of mature, electrically quiescent ventricular cardiomyocytes (devoid of any pacemaker cells) will help attenuate graft-related arrhythmias. In separate unpublished work in the guinea pig MI model, our laboratory has recently found that more mature cardiomyocyte populations are indeed capable of robust engraftment and form grafts with enhanced electrophysiological function by optical mapping (data not shown). The guinea pig does not predict the graft-related arrhythmias observed in large-animal models, but we are currently in the process of scaling up the manufacture of these more mature myocytes to the large cell quantities required for pig transplantation experiments. Of course, we also plan studies in the pig model to determine the effects of conventional anti-arrhythmic drugs on graft-related arrhythmia incidence.

There are several other critical hurdles to the successful development of a PSC-based regenerative therapy that we intend to address using the highly relevant pig transplantation model described here. Given its higher throughput and greater size relative to non-human primates, the pig is well suited to tackle practical issues related to cell dosing, delivery vehicle, and the timing of administration. We also expect the pig to be particularly helpful in validating alternative routes of hESC-CM delivery. While a recent clinical trial demonstrated the feasibility of the direct surgical implantation of PSC derivatives via an open-chest approach (Menasche et al., 2018), a minimally invasive delivery strategy would obviously be eventually preferred. Image-guided transendocardial injection catheters have been used to successfully deliver other candidate cell types to the pig heart (Strauer and Steinhoff, 2011), but they will need to be tested for compatibility with hESC-CMs. Finally and most importantly, we still need to complete a pivotal efficacy study to test the hypothesis that hESC-CM transplantation will mediate durable, beneficial effects on LV contractile function in the pig MI model. One limitation of the present feasibility study was that we deliberately sought to optimize cardiomyocyte engraftment by inducing relatively small infarct scars (∼16% of LV mass). As anticipated, these animals showed a correspondingly small decrement in LVEF, and contractile function typically rebounded in both cell- and vehicle-treated animals by 4 weeks post transplantation. Hence in future work, we intend to test an appropriately powered study involving a larger cohort of animals with bigger infarcts and a longer duration of follow-up to determine whether this regenerative strategy can improve heart function in the pig MI model.

## Experimental Procedures

### Production of hESC-Derived Cardiomyocytes

Undifferentiated hESCs were expanded and differentiated into cardiomyocytes in suspension cultures using stirred-tank bioreactors and the experimental sequence depicted in Figure 1A. See Supplemental Experimental Procedures for detailed cardiomyocyte manufacturing methods.

### Animal Procedures

All animal studies were approved and conducted in accordance with the Animal Care Committee of Sunnybrook Research Institute. Yorkshire pigs (Caughell Farms, ON) underwent MI induction via balloon occlusion of the mid-LAD, followed 3 weeks later by thoracotomy and direct transepicardial injection of hESC-CMs or vehicle. Animals were pharmacologically immunosuppressed with cyclosporine A, methylprednisolone, and abatacept, and underwent telemetric ECG monitoring, serial cardiac MRI, and/or terminal EAM as described in Figure S1 and Supplemental Experimental Procedures.

### Histological Studies

Vehicle and hESC-CM-engrafted hearts were uniformly sliced at 5-mm intervals, and whole-mount transverse sections were subjected to immunohistochemical studies as described in Supplemental Experimental Procedures.

## Author Contributions

R.R. and M.A.L. designed the study, developed the experiments, and wrote the manuscript. R.R. performed all histology. R.R., H.M., and A.L. performed data analysis. A.P.S., S.M., K.M., and K.N. performed EAM studies and subsequent interpretation and analysis. R.R., B.Q., J.B., H.K., J.W., R-K.L., G.A.W., and N.R.G. performed animal procedures. J.B., X.Q., and N.R.G. performed cardiac MRI. T.V.S., J.R., K.M.P., E.T., P.W.Z., and G.K. generated hESC-CMs.

## Supplemental Information

Document S1. Supplemental Experimental Procedures, Figures S1–S6, and Tables S1 and S2

Document S2. Article plus Supplemental Information
