# Supplementary material for: Human Embryonic Stem Cell-Derived Cardiomyocytes Regenerate the Infarcted Pig Heart but Induce Ventricular Tachyarrhythmias
Source: Stem Cell Reports. 2019 May 2;12(5):967–81. doi: 10.1016/j.stemcr.2019.04.005 (PMC6524945; doi:10.1016/j.stemcr.2019.04.005)
Supplement: Document S1. Supplemental Experimental Procedures, Figures S1–S6, and Tables S1 and S2 [file mmc1.pdf]

Stem Cell Reports, Volume *12*

Supplemental Information

Human Embryonic Stem Cell-Derived Cardiomyocytes Regenerate the Infarcted Pig Heart but Induce Ventricular Tachyarrhythmias

Rocco Romagnuolo, Hassan Masoudpour, Andreu Porta-Sánchez, Beiping Qiang, Jennifer Barry, Andrew Laskary, Xiuling Qi, Stéphane Massé, Karl Magtibay, Hiroyuki Kawajiri, Jun Wu, Tamilla Valdman Sadikov, Janet Rothberg, Krishna M. Panchalingam, Emily Titus, Ren-Ke Li, Peter W. Zandstra, Graham A. Wright, Kumaraswamy Nanthakumar, Nilesh R. Ghugre, Gordon Keller, and Michael A. Laflamme

Supplemental Figures

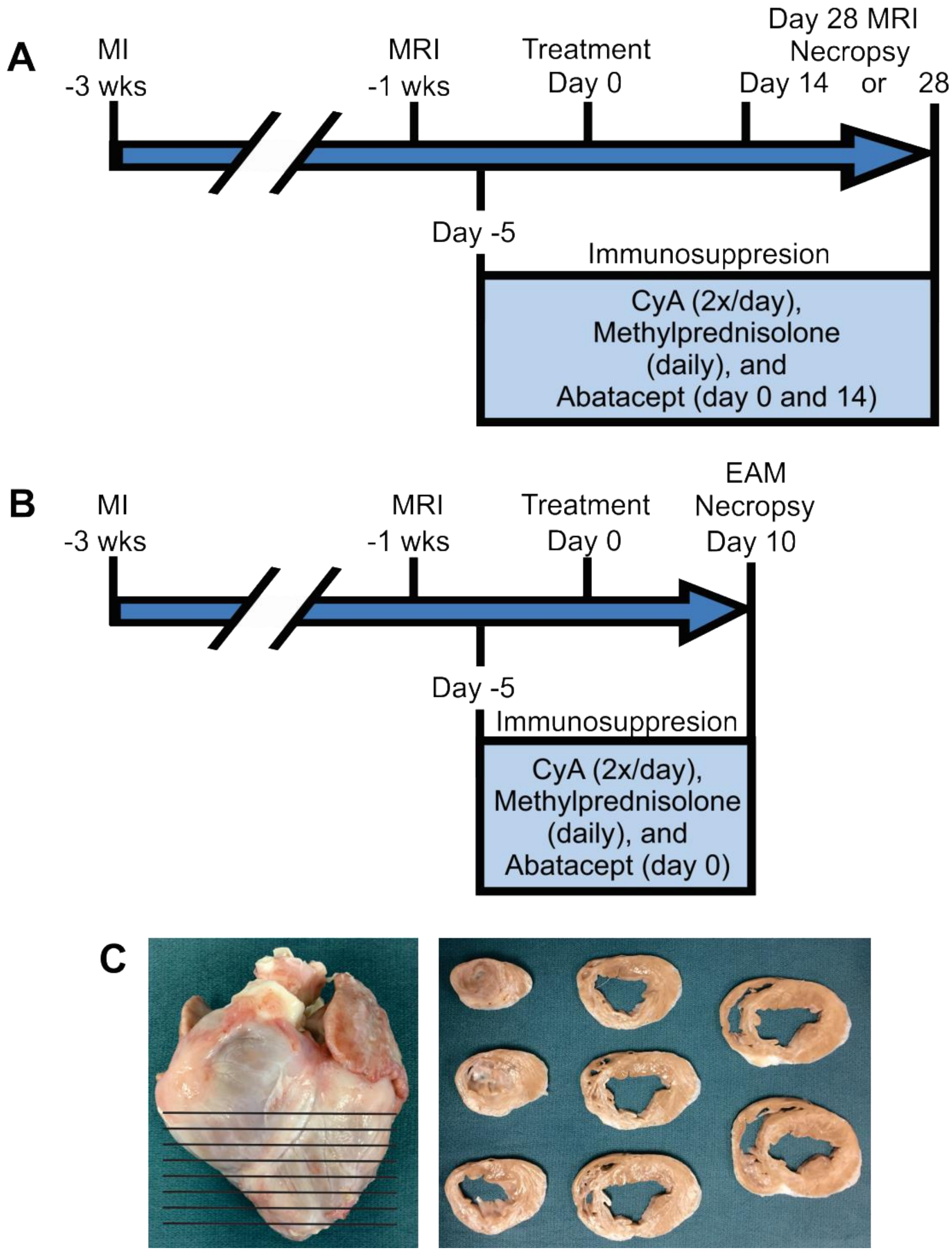

Figure S1. Sequence of experimental animal procedures. Related to Experimental Procedures.
Myocardial infarction (MI) was induced by balloon occlusion 3 weeks prior to direct intramyocardial transplantation of either hESC-CMs or vehicle. **A:** Most pigs were followed for 4 weeks post-transplantation and underwent serial MRI scans at baseline (pre-MI), 1 week prior to transplantation (week -1), and 4-weeks post-transplantation. **B:** A subset of animals were instead subjected to terminal electroanatomical voltage mapping (EAM) and pacing studies at 10 days-post-transplantation. The immunosuppression regime is outlined in **A** and **B**. **C:** Following necropsy, the heart was fixed and sectioned at 5 mm intervals from apex to base, and transverse sections were embedded for whole-mount histology.

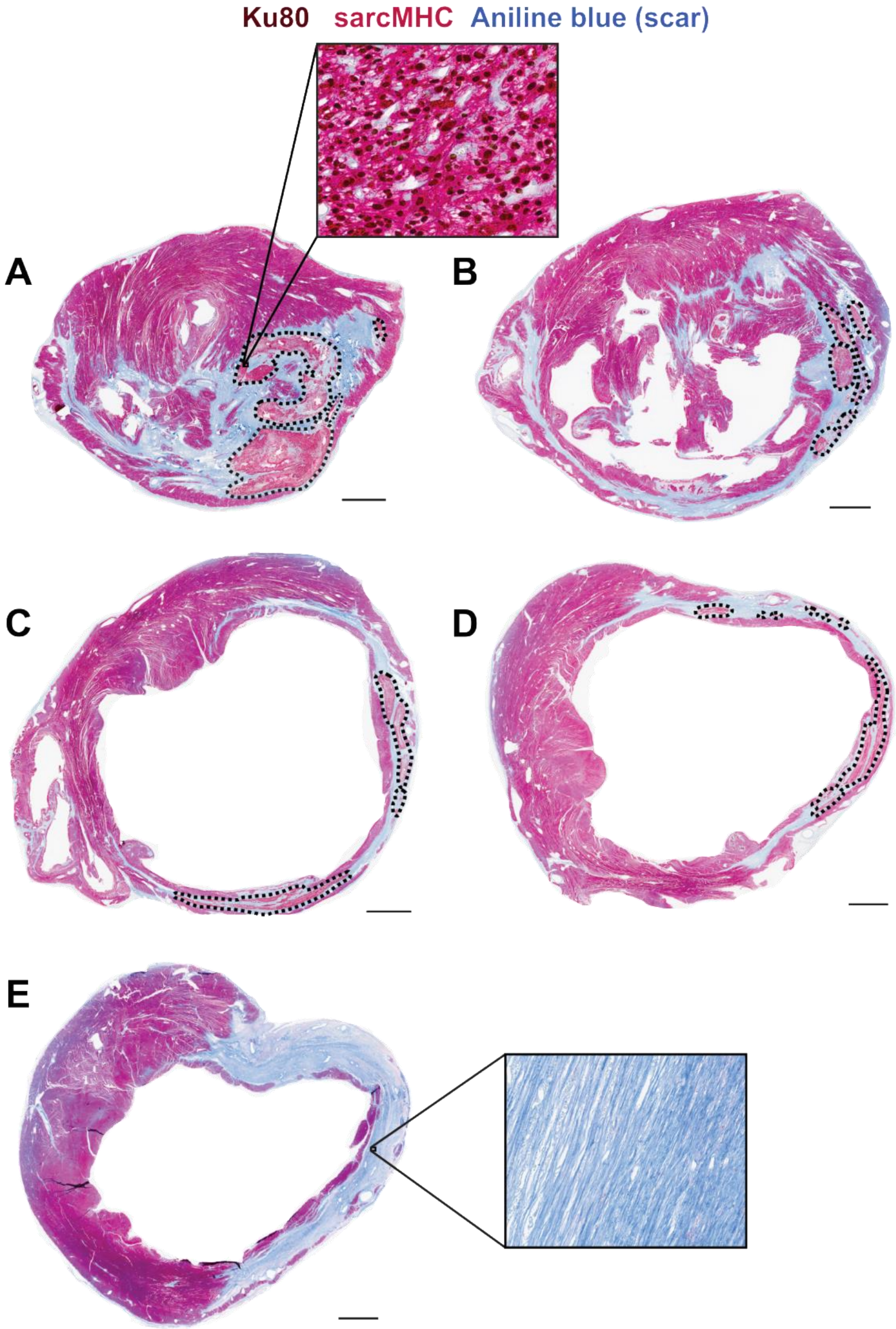

Figure S2. Transverse sections through hearts of representative hESC-CM and vehicle recipients. Related to Figure 2.

A-D: Whole-mount sections of an hESC-CM engrafted heart transversely sliced at 5mm intervals showing abundant human myocardial graft (outlined by dotted lines; A-D). **E:** By contrast, no human-specific Ku80 positive areas were identified in a representative vehicle recipient. Sections from all hearts were stained for sarcomeric myosin heavy chain (red), human-specific Ku80 (brown nuclei), and scar (blue). Scale bar = 5 mm.

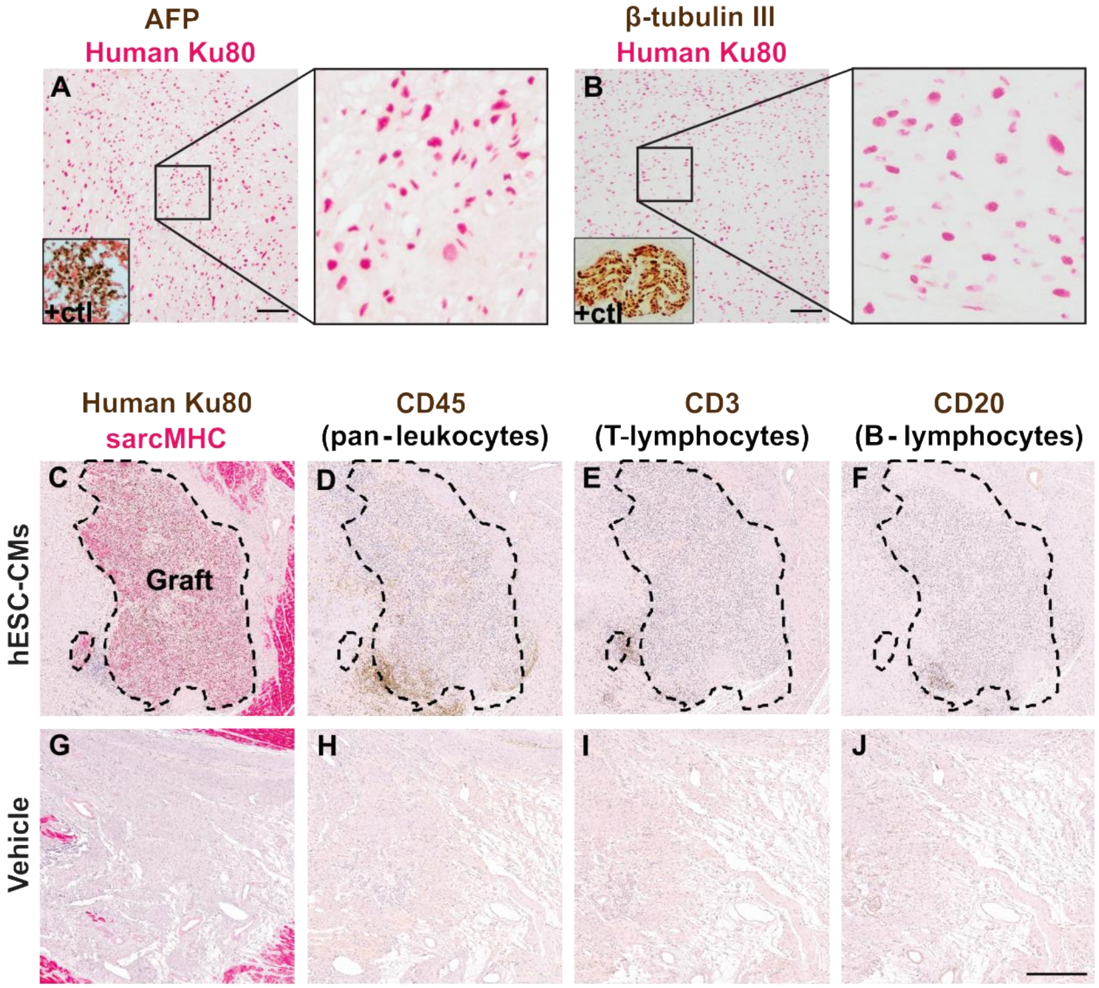

Figure S3. Additional immunohistochemistry to evaluate non-cardiac graft elements and host immune responses. Related to Figures 3 and 4.

A-B: Grafts were uniformly negative for α-1-fetoprotein (AFP, panel **A**) and β-tubulin III (panel **B**), with the insets showing positive controls (day 6 embryoid bodies cultured in 20% FBS or host porcine nerves, respectively). Scale bar = 100 µm. While most hESC-CM graft tissue showed little or no infiltration by host leukocytes, there were occasional grafts that showed evidence of mild cellular rejection. To illustrate this, panels show adjacent histological sections taken from a hESC-CM recipient with a more-than-typical immune response (panels **C-F**) or a vehicle control (panels **G-J**), both at 28-days post-transplantation. **C, G:** Host and graft myocardium were identified by dual-immunostaining for sarcomeric myosin heavy chain (sarcMHC; red) and human-specific Ku80 (brown nuclei). Note the cluster of host mononuclear cells at the lower left-hand corner of the graft in panel **C.** These infiltrates were then evaluated on adjacent histological sections using antibodies against CD45 (pan-leukocytes, **D**, **H**), CD3 (T-lymphocytes, **E**, **I**), and CD20 (B-lymphocytes, **F**, **J**). Scale bar = 500 µm.

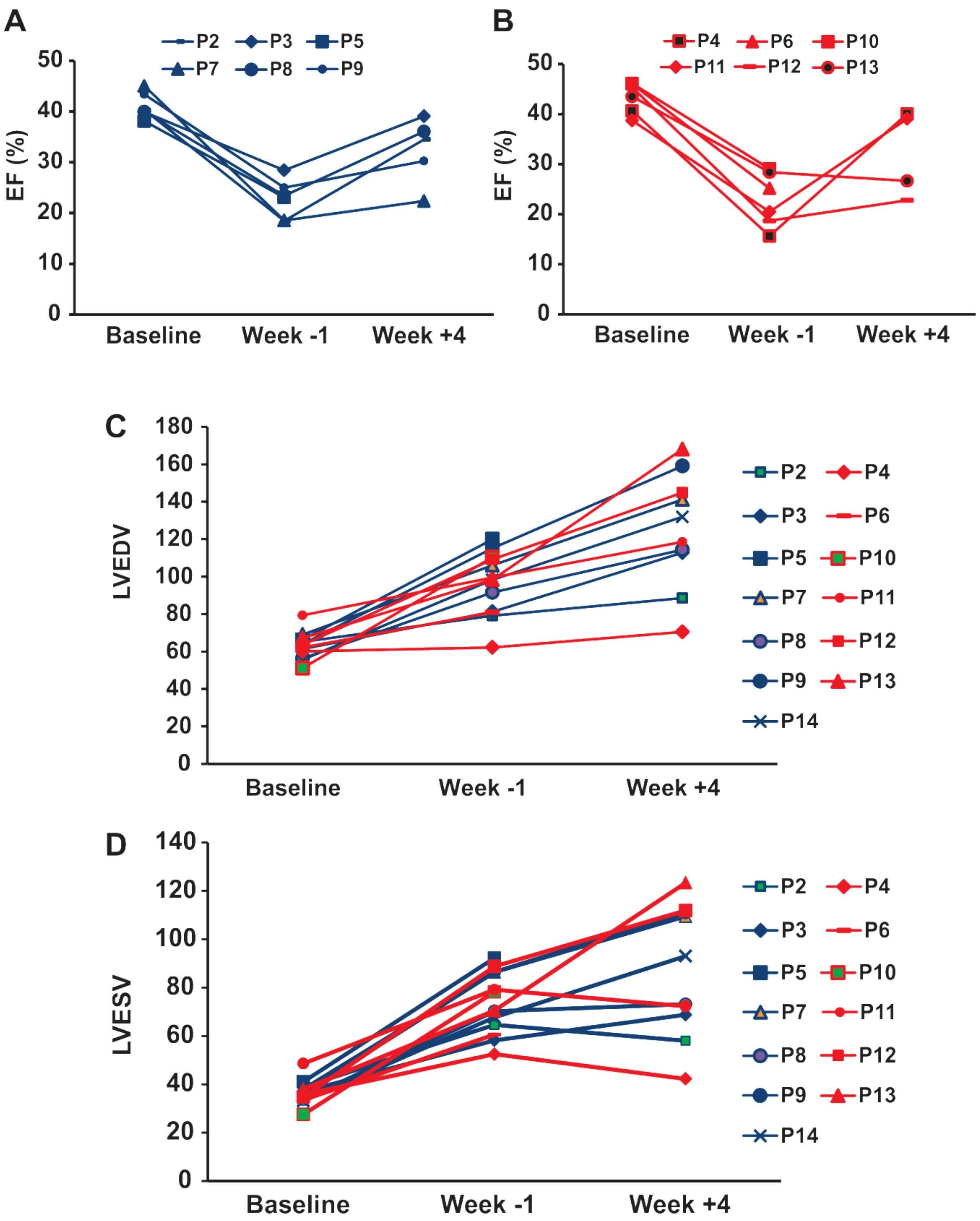

Figure S4. LV dimensions and contractile function determined by cardiac MRI. Related to Figure 5.
A,B: LVEF in infarcted pigs receiving vehicle (**A;** n = 6 pigs) or hESC-CMs (**B;** n = 6 pigs). **C,D**: LVEDV (**C**) and LVESV (**D**) in infarcted pigs receiving vehicle (blue lines; n = 7 pigs) or hESC-CMs (red lines; n = 6 pigs) at baseline, 1-week prior to treatment (week -1), and 4-weeks post treatment (week +4).

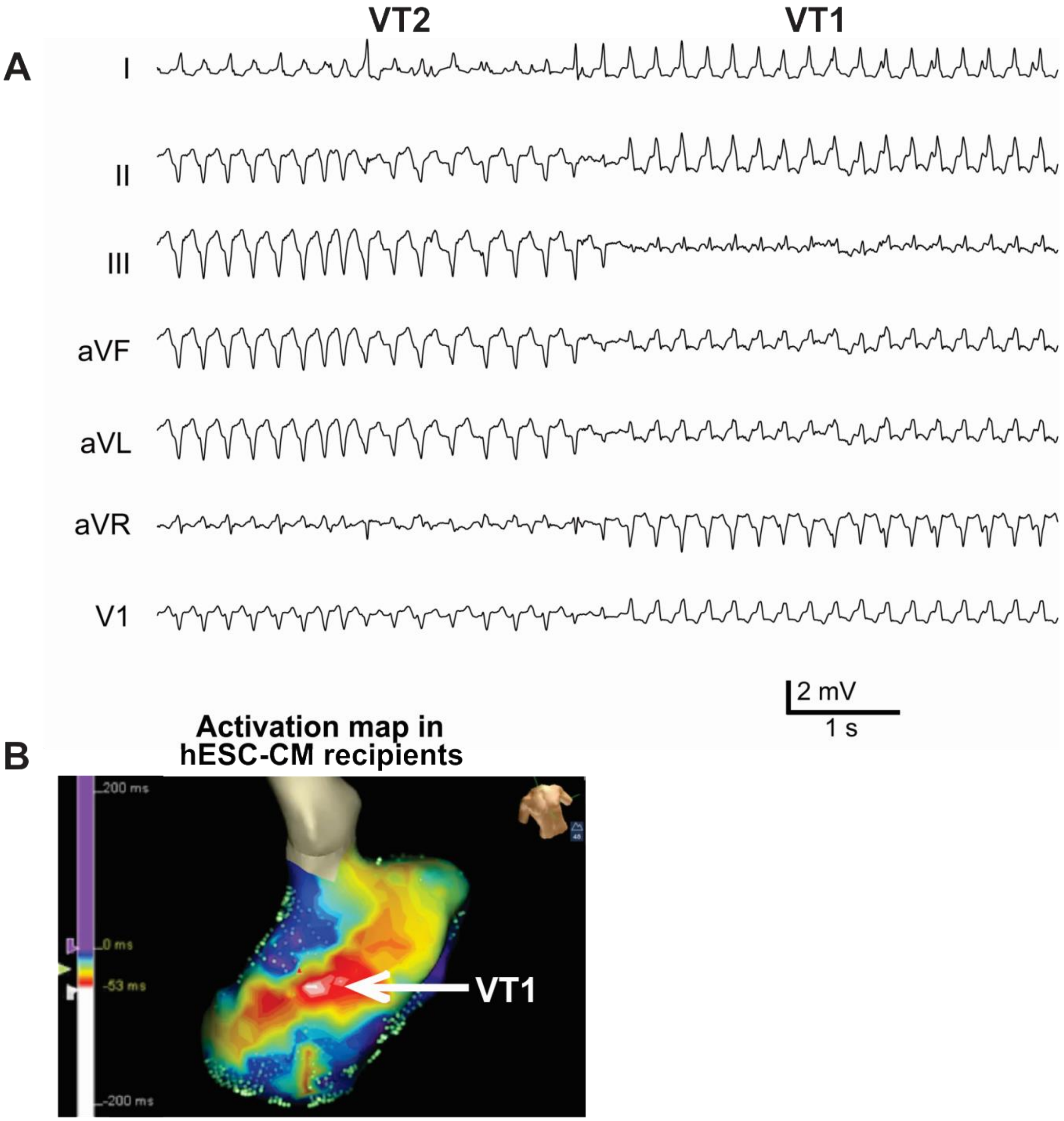

Figure S5. Electrophysiological studies in an hESC-CM recipient with two different VT morphologies. Related to Figure 7.

An infarcted pig (animal P21) underwent EAM and standard clinical electrophysiological studies 10 days following hESC-CM transplantation. **A:** Surface ECG showing monomorphic VT with a superior axis and a rate of 242 bpm (VT2) that showed highly variable activation rates and morphology in transition to an inferior axis and a rate of 261 bpm (VT1). VT1 predominated throughout the procedure. **B:** LAT map acquired during VT1 with VT2 being too transient to fully map. Earliest areas are depicted in white and red and late activated areas are depicted in a gradient from orange to yellow to green to light blue and dark blue.

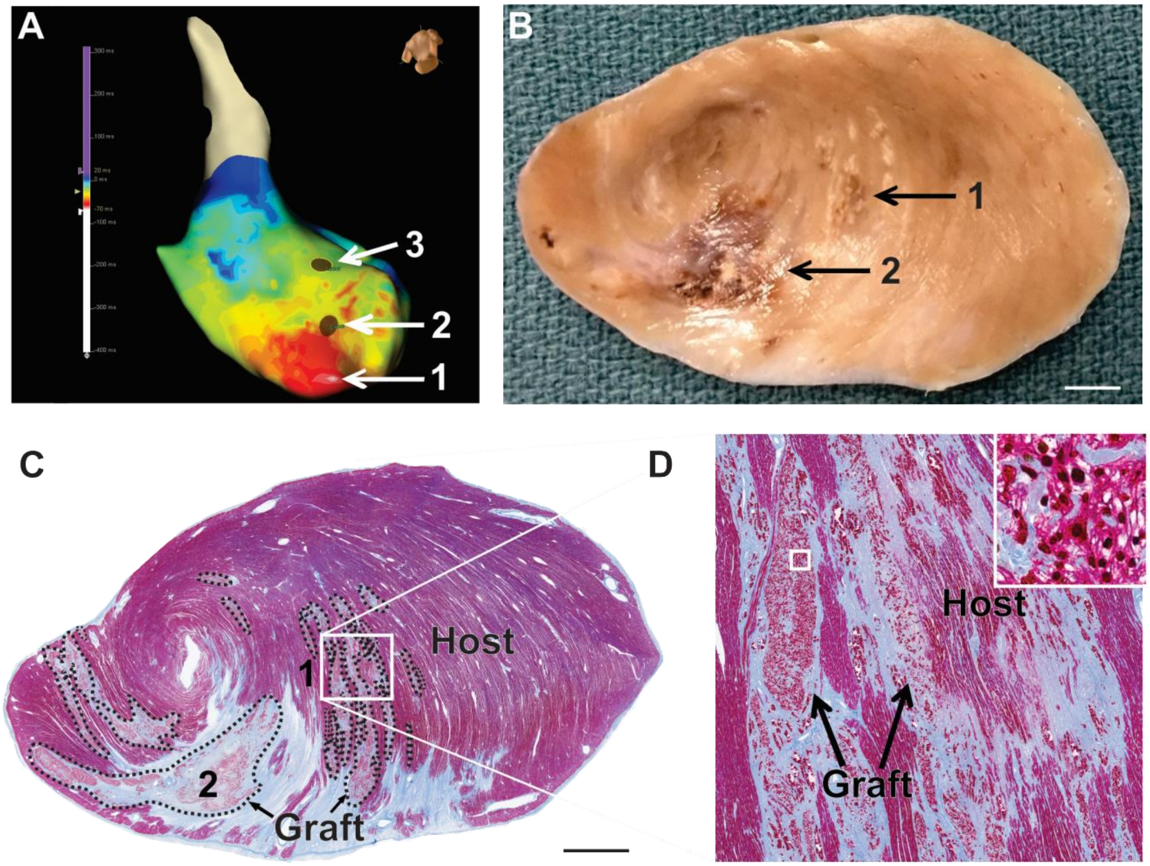

Figure S6. Correlation between EAM and hESC-CM graft location by histology. Related to Figure 7.
A: LAT map from hESC-CM recipient (animal P20) acquired during ventricular tachycardia (VT). Using an ablation catheter, three lesions were applied including one mark on the epicardial surface precisely at the site of earliest activation (denoted as site #1) and two marks on the endocardial surface a known distance lateral to it (sites #2 and 3). **B:** Transverse section of this same heart taken from an apical level corresponding to the site of earliest activation showing grossly discernible ablation marks 1 and 2 (arrows). **C:** Whole-mount histological section corresponding to the image in panel B stained for sarcomeric myosin heavy chain (red), human-specific nuclear marker Ku80 (brown), and scar (blue). Regions of graft tissue located in the scar and border zone are indicated by the dotted lines, along sites of thermal injury and hemorrhage corresponding to ablation marks #1 and 2. **D:** Higher-magnification inset from panel **C** containing a point of contact between host and graft myocardium and representing the site of earliest endocardial activation. Scale bar = 5mm.

Table S1. Summary of pigs receiving hESC-CMs. Related to Figures 2, 3, 4, and 6.

ID	Cell type	ESC-CM purity (cTnT%)	ESC-CM purity (MLC2v%)	Post-Thaw viability	Graft size (% of infarct	VT burden (total hrs)	Graft purity (%cTnT)
**P1**	HES-2	47.1	18.6	92.2	N.D	N.D*	64.2
**P4**	HES-2	81.4	8.0	81.4	29.1	187.8	52.3
**P6**	ESI-17	86.0	68.3	85.4	8.4	254.0	86.3
**P10**	ESI-17	87.1	38.5	81	8.2	123.2	90.6
**P11**	ESI-17	86.7	31.4	72.5	0.1	69.8	N.D
**P12**	ESI-17	87.0	17.1	69.8	17.1	54.3	94.3
**P13**	ESI-17	88.4	41	79.5	7.6	293.9	93.3
**P19**	ESI-17	88.8	34.5	N.D	15.4	N.D**	94.5
**P20**	ESI-17	84.0	41.1	77.2	22.4	N.D**	91.7
**P21**	ESI-17	82.4	18.2	87.3	28.6	N.D**	95.2
	**Avg ±SEM**	**81.9±3.9**	**31.7±5.5**	**80.7±2.4**	**15.2±3.4**	**163.8±40**	**84.8±5.2**

N.D = Not Determined; *P1 was not fitted with DSI telemetry device. At the time of sacrifice, he was found to be in VT; **P19-P21 were used for EAM experiments and only kept to day 10 post-transplantation where they were all in sustained VT.

Table S2: Summary of MRI data on either vehicle or hESC-CMs recipients. Related to Figure 5.

ID	Treatment	Endpoint	Infarct size (%LV)- MRI Week -1	Infarct size (%LV)- MRI Week +4	EF% Baseline	EF% Week -1	EF% Week +4
**P2**	Vehicle	4-week	11.2	7.7	40.3	18.5	34.5
**P3**	Vehicle	4-week	11.5	5.8	39.9	28.4	39.1
**P5**	Vehicle	Sacrificed day 14	19.6	N.D	38.1	23.1	N.D
**P7**	Vehicle	4-week	21.4	N.D	45.0	18.5	22.3
**P8**	Vehicle	4-week	15.2	13.3	40.0	23.4	36.1
**P9**	Vehicle	4-week	21.3	13.0	43.4	25.0	30.2
**P14**	Vehicle	4-week	18.9	13.7	40.4	31.1	29.5
**P1**	HES-2 CM	2-week	16.6	10.3	N.D	35.3	N.D
**P4**	HES-2 CM	4-week	10.8	9.0	40.6	15.7	40.0
**P6**	ESI-17 CM	Died at day 16	12.6	N.D	46.0	25.2	N.D
**P10**	ESI-17 CM	Sacrificed day 13	19.3	N.D	46.1	29.1	N.D
**P11**	ESI-17 CM	4-week	13.0	12.5	38.7	20.5	39.0
**P12**	ESI-17 CM	4-week	20.2	10.2	45.4	18.7	22.8
**P13**	ESI-17 CM	4-week	16.6	14.4	43.5	28.4	26.7
**P15**	Vehicle	EAVM-day 10	14.5	N.D	42.1	26.5	N.D
**P16**	Vehicle	EAVM-day 10	15.6	N.D	42.4	25.3	N.D
**P17**	Vehicle	EAVM-day 10	N.D	N.D	N.D	N.D	N.D
**P18**	Vehicle	EAVM-day 10	N.D	N.D	N.D	N.D	N.D
**P19**	ESI-17 CM	EAVM-day 10	14.5	N.D	34.5	27.0	N.D
**P20**	ESI-17 CM	EAVM-day 10	10.6	N.D	48.0	31.3	N.D
**P21**	ESI-17 CM	EAVM-day 10	15.8	N.D	43.5	27.8	N.D

SUPPLEMENTAL EXPERIMENTAL PROCEDURES

Production of hESC-derived cardiomyocytes

The scaled expansion of undifferentiated hESCs and their subsequent differentiation into cardiomyocytes was performed by the Centre for Commercialization of Regenerative Medicine (CCRM, Toronto, ON, Canada) using methods adapted from a previously reported stirred-tank bioreactor system (Chen et al., 2015; Prowse et al., 2014). While the vast majority of transplantation studies were conducted using cardiomyocytes generated using the GMP-pedigree ESI-17 hESC line (BioTime, Alameda, CA, USA) (Chen et al., 2015), two pigs (P1 and P4) received cardiomyocytes derived from HES-2 hESC line (BioTime, WiCell, Madison, WI, USA) in an initial pilot study. hESCs from both lines were expanded in the undifferentiated state using mTESR medium (StemCell Technologies, Vancouver, BC, Canada), then guided into cardiomyocytes using the differentiated protocol depicted in **Figure 1A** (Chong et al., 2014). For this, hESC aggregates in either 125 mL or 1L bioreactors were transferred into complete StemPro (Thermo Fisher, Waltham, MA, USA) cardiomyocyte differentiation media containing L-glutamine (2 mM, Thermo Fisher, Waltham, MA, USA), transferrin (150 µg/mL, Sigma-Aldrich, St. Lois, MO, USA), monothioglycerol (MTG; 50µg/mL, Sigma-Aldrich, St. Lois, MO, USA), ascorbic acid (50 mg/mL, Sigma-Aldrich, St. Lois, MO, USA) and ROCK inhibitor Y-27632 (RI; 10 µM, Tocris Bioscience, Oakville, ON, Canada) and then were serially treated with activin A (6ng/mL, R&D, Minneapolis, MN, USA), bone morphogenetic protein-4 (10 ng/mL, R&D, Minneapolis, MN, USA) and basic fibroblast growth factor (5ng/mL, R&D, Minneapolis, MN, USA) for mesoderm induction. Two days post-induction, media was replaced with iWP2 (2 µM, Tocris Bioscience, Oakville, ON, Canada) and ROCK inhibitor (10 µM) in StemPro cardiomyocyte differentiation media for two days, followed by complete media exchange with StemPro cardiomyocyte differentiation media every two days thereafter until harvesting. One day prior to harvesting, on day 16 post-induction, cell aggregates were transiently heat shocked (42° C for 30 minutes) in StemPro cardiomyocyte differentiation media (lacking MTG and ascorbic acid) to improve graft cell survival as previously reported (Laflamme et al., 2007; Laflamme et al., 2005). Twenty-four hours following heat shock, cell aggregates were dispersed enzymatically to single cells with collagenase II (1g/L in HBSS, Worthington Biochemical Corporation, Lakewood, NJ, USA) for 2 hours at 37°C on an orbital shaker, pelleted, resuspended in TrypLE (Life Technologies, Carlsbad, CA, USA) with DNase I (10 µg/mL, EMD Millipore, Etobicoke, ON, Canada), then cryopreserved as previously described (Xu et al., 2011). On the day of transplantation, cells were thawed at 37°C and cell counts and post-thaw viability was determined using the nucleocounter NC-200 automated cell counter (Chemometec, Gydevang, Denmark). The cells were then washed with RPMI-1640 media, and suspended in 3.0 mL of a previously reported pro-survival cocktail (Laflamme et al., 2007) consisting of growth factor-reduced Matrigel (~60% v/v), supplemented with cyclosporine A (200 nM, Sandimmune, Novartis, Mississauga, ON, Canada) and pinacidil (50 µM, Sigma-Aldrich, St. Lois, MO, USA). Cell viability post-thaw was routinely assessed with acridine orange/DAPI staining and quantification with a Nucleocounter NC-200 automated cell counter (ChemoMetec A/S, Allerod, Denmark) (**Table S1**). Grafts at either 2- or 4-weeks post-transplantation were routinely stained with caspase-3 (Cell Signaling Technology, Danvers, MA, USA) to determine the number of apoptotic cells (<0.1% of graft cells were caspase-3 positive).

Animal procedures

All animal studies were approved and conducted in accordance with the Animal Care Committee of Sunnybrook Research Institute. For all procedures, 20-30 kg male Yorkshire pigs (Caughell Farms, Fingal, ON, Canada) were fasted overnight and then anesthetized by intramuscular administration of a cocktail consisting of atropine (0.05mg/kg) and ketamine (33mg/kg), followed by maintenance with 5% inhaled isoflurane. The sequence of experimental animal procedures is depicted in **Figure S1**.

<u>Myocardial infarction:</u> MI induction was performed as previously reported (Ghugre et al., 2011). In brief, animals received a pre-operative bolus of amiodarone (75 mg), lidocaine (20 mg bolus, 3 mg/kg/hour infusion), and heparin (100 IU/kg iv). Under X-ray fluoroscopic guidance (Veradius C-arm System, Philips Healthcare, Markham, ON, Canada) with iodinated contrast, complete occlusion of the mid left anterior descending coronary artery was performed for 90 minutes via inflation of a percutaneous balloon dilation catheter (Sprinter Legend Balloon Catheter, Medtronic, Minneapolis, MN, USA), followed by reperfusion. Pigs showing infarct sizes less than 10% of LV mass at their initial scan pre-transplantation were excluded from the study (n=4 animals).

<u>Immunosuppression:</u> To facilitate the intravenous administration of immunosuppressive drugs and routine blood sampling, all animals (including control and hESC-CMs recipients) underwent placement of an indwelling vascular access port (Access Technologies, Skokie, IL) in the external jugular vein at 2 weeks post-MI (Henderson et al., 2003). All pigs (vehicle and hESC-CM recipients) were treated with an immunosuppression regimen including

Orencia (Abatacept CTLA4 immunoglobulin, Bristol-Myers Squibb, Mississauga, ON, Canada), given at 12.5 mg/kg on day of hESC-CM transplantation and every 2 weeks thereafter); methylprednisolone (Pfizer, Mississauga, ON, Canada), given as 250 mg on day of hESC-CM transplantation followed by a taper to 125 mg per day over two weeks and then 125 mg daily maintenance thereafter); and cyclosporine A (Neoral, Novartis, Mississauga, ON, Canada), given as 10-16 mg/kg PO twice per day to achieve trough concentrations of 250 µg/L, administered from 5 days prior to hESC-CM transplantation daily until sacrifice. Blood was drawn and cyclosporine A was measured 1-2 times per week to ensure adequate trough levels (Toronto General Hospital Laboratory Medicine and Pathology Department).

Thoracotomy and cell implantation: hESC-CMs were directly injected into the infarct scar using a transepicardial delivery approach adapted from prior work in the non-human primate model (Chong et al., 2014). On day 20 post-MI, pigs were anesthetized and underwent left thoracotomy as previously described (McCall et al., 2012). The fourth intercostal space was exposed and opened adjacent to the lower rib, and the space was enlarged under direct vision using a self-retaining rib retractor. After opening the pericardium anteriorly, the apex and the anterior LV are gently exposed using warm saline-wet gauze placed beneath the heart. hESC-CMs in pro-survival cocktail or vehicle alone were then directly injected into the infarct zone using a manually curved 27G needle via 12 injections of 250 µL each. Before closing the chest, a telemetric ECG sensor was implanted as described below.

Telemetric ECG: All animals were continuously monitored from the time of cell transplantation until sacrifice via non-invasive telemetric ECG (Ponemah Physiology Platform, Data Sciences International (DSI), St. Paul, MN, USA). After completion of the thoracotomy, M01 telemetry devices (DSI) were installed with the leads placed within the chest wall adjacent to the base and apex of the heart. Electrocardiograms (ECG) were collected from the implanted device from all animals and telemetrically recorded continuously using Ponemah software (DSI). ECG traces were evaluated with Data Insights analysis software (DSI) and verified manually to determine all incidences of VT, defined as a run of 4 or more premature ventricular complexes (PVCs). Heart rate was measured at two points (12 am and 12pm) on days 1, 10 and 26 for vehicle and hESC-CMs recipients with averages and standard error of the mean being reported.

Cardiac MRI: Parameters including LV dimensions, LV ejection fraction (LVEF) and infarct size were assessed using the gold-standard technique of cardiac MRI. MRI scans were performed at baseline (i.e. prior to MI), at 2-weeks post-MI and at 4-weeks post-transplantation (corresponding to 7-weeks post-MI) using methods as previously reported (Ghugre et al., 2011). In brief, MRI scans were acquired using a 3T MR750 scanner (GE Healthcare, Markham, ON, Canada) and following three planes localization, multiple slices (5 mm thick) through the myocardium were obtained using steady-state free precession sequence in cine mode (FIESTA, GE Healthcare, Markham, ON, Canada) for global LV function. Contrast injection of Gadolinium-DTPA (0.2mg/Kg) was used to quantify infarction/scar size with a T1-weighted IR-FGRE sequence; late gadolinium enhancement (LGE) imaging was performed at 8-10 min post contrast injection. As determined by LGE-MRI, infarcts sizes ranged between 1.2-21.4% of LV mass, and animals showing infarct sizes <10% were excluded from the study (n=4). Regional wall thickness was determined as an average of segments 7 and 8 corresponding to the mid ventricular anterior and anteroseptal myocardium, respectively, as per AHA guideline recommendations (Cerqueira et al., 2002). These segments corresponded to the site of injection for vehicle and hESC-CM transplantation (i.e. the core region of the infarct). Offline analysis of MRI data was carried out using CVI42 software (Circle Imaging, Calgary, AB, Canada).

Electroanatomical mapping: Animals underwent terminal EAM mapping at 10 days post-thoracotomy and intra-cardiac implantation of hESC-CMs or vehicle. Endocardial mapping of the LV was performed via a transaortic retrograde approach using the Abbott Precision™ Research version mapping system and an HD16 grid™ catheter (Abbott Medical, Minneapolis, MN, USA) with 16 1mm-diameter electrodes (4mm center to center electrode spacing arranged in a fixed spaced array). All electrograms were acquired with filtering at 0.5-300Hz allowing for unipolar and bipolar data analysis. A quadripolar catheter was placed in the inferior vena cava and right ventricle (RV) apex as the unipolar reference. Acquisition of electrograms and projection onto the anatomical surfaces was performed with a threshold of 7mm of internal projection and 7mm interpolation. Field scaling was applied for all maps with low voltage areas defined as <1.5mV (Tung et al., 2016). Annotation of timing of bipolar electrograms was taken based on the maximum negative dV/dt of the bipolar signal as described previously (Cantwell et al., 2015). Electroanatomical maps were then registered with the MRI volumes offline using the aorta and the LV surface as fiducial points for better anatomical correlation with the location of the infarct. We also applied radiofrequency (RF) energy to "mark" the tissue at known distances relative to the site of earliest activation to allow for an even more precise correlation between histology and EAM in a subset of animals. For this, RF energy (30

Watts, 30 seconds, temperature controlled to 65ºC) was delivered with a non-irrigated FlexAbility™ Abbott ablation catheter.

In two hESC-CM recipients, epicardial mapping was also performed to rule out the possibility of an epicardial reentrant circuit. For this procedure, we used the same Abbott Precision™ mapping system and gained access via the subxiphoid approach as described previously (Sosa et al., 1996).

After the completion of EAM studies, we used standard clinical electrophysiological maneuvers to either: 1) assess arrhythmia vulnerability in infarcted vehicle controls, all of which were in normal sinus rhythm, or 2) help elucidate the mechanistic basis of spontaneous VT in infarcted hESC-CM recipients, all of which were already in VT at the time of the procedure. To assess VT inducibility in the former experimental group, we applied programmed ventricular stimulation consisting of a pacing train (at either 400 or 500ms cycle length (CL)) followed by the application of up to three extra-stimuli coupled to 190ms. In the case of hESC-CM recipients, overdrive pacing and entrainment of VT were performed from the RV outflow tract at varying CLs. RV pacing was used in P19, 20 and 21, while P20 was also paced at a site close to the focus of earliest activation.

Histological studies

All hearts were fixed with 10% neutral buffered formalin, transversely sectioned at 5 mm intervals on a commercial slicer (Berkel 827A-PLUS, Bonner Springs, KS, USA), processed and paraffin-embedded for histological analyses. Six-micrometer-thick sections were then immunostained using previously reported methods (Laflamme et al., 2007) and primary antibodies directed against cardiac antigens including sarcomeric myosin heavy chain (Developmental Studies Hybridoma Bank (DSHB), Iowa City, IA, USA: cat# MF 20), cardiac troponin T (DSHB: cat# CT3), α-actinin (clone EP2529Y; Abcam, Cambridge, UK: cat# ab68167), titin (DSHB; cat# 9 D10), myosin light chain 2V (Abcam: cat# ab79935), myosin light chain 2A (Sigma-Aldrich, St. Louis, MO, USA: cat# HPA013331), cardiac troponin I (abcam: cat# ab47003), slow skeletal troponin I (Novus Biologicals, Oakville, ON, CA: cat# NBP1-56641), caveolin-3 (Abcam: cat# ab2912), pan-cadherins (Sigma-Aldrich: cat# C3678) and connexin 43 (Abcam: cat# ab11370). Graft cell origin was rigorously confirmed by dual-immunolabeling with a human-specific antibody against the nuclear marker Ku80 (Allard et al., 2014; Weinberger et al., 2016) (Cell Signaling Technology: cat# 2180). To evaluate for the presence of non-cardiac graft elements, we also immunostained with human-specific antibodies against the following cell types: endothelium (CD31/PECAM, Agilent, Santa Clara, CA, USA: cat# M082301-2), epithelium (pan-cytokeratin cocktail AE1/AE3, Agilent: cat# M351501-2), and fibroblasts (clone TE-7, Millipore Sigma, Etobicoke, ON, Canada: cat# CBL271). Host endothelial cells were identified using a species specific antibody against von Willebrand factor (Cloud-Clone Corp., Katy, TX, USA: cat# PAA833Po01), and graft cell proliferation was assessed using a human specific antibody against the nuclear antigen Ki-67 (Agilent, Santa Clara, CA, USA: cat# M724029-2) and phospho-histone H3 (Ser10) (Cell Signaling Technology, Danvers, MA, USA: cat# 9701). H&E, β-tubulin III (Sigma-Aldrich: cat# T8578) and α-1-fetoprotein (Agilent: A0008) were used to detect the presence of any teratomas. CD20 (Biocare Medical, Pacheco, CA, USA: cat#3004), CD3 (Agilent: cat# A0452), and CD45 (Bio-Rad, Mississauga, ON, Canada: cat# MCA1447) were used to detect B-lymphocytes, T-lymphocytes, and pan-leukocytes respectively. Secondary antibodies were either Alexa-conjugated (Thermo Fisher, Waltham, MA, USA: cat# A28175, A27034, A11037, A11032, A28181, A27040) for confocal immunofluorescence or biotinylated (Vector Labs, Burlingame, CA, USA: cat# BA-9200 and BA-1000) for brightfield detection (ABC kit followed by alkaline phosphatase/Vector Red or HRP/DAB, Vector Labs, Burlingame, CA, USA). Scar and graft size was either determined by picrosirius red (Polysciences Inc., Warrington, PA, USA) or aniline blue staining (25g/L in 2% acetic acid) from two whole mount sections originating at 5 and 15mm from apex and quantified using ImageJ software (National Institutes of Health, Bethesda, MD). To suppress autofluorescence seen in tissues analyzed with confocal microscopy, after secondary antibody incubation and washing, the tissue was incubated with 10mM culpric sulfate in 50 mM ammonium acetate buffer (pH 5) for 5 minutes, rinsed with PBS, incubated with Hoechst 33342 Fluorescent Stain (Thermo Fisher, Waltham, MA, USA) and then mounted with ProLong Diamond Antifade Mountant (Thermo Fisher).

Immunofluorescence microscopy was performed using a Zeiss LSM700 inverted confocal microscope with either a FLUAR 10x/0.50 NA, Plan-Apochromat 20x/0.8 NA, Plan-Apochromat 40x/1.4 NA oil immersion, or Plan-Apochromat 63x/1.4 NA oil immersion objective. Images were acquired using the LSM Zen 2012 acquisition software and processed with Corel Draw Graphics Suite 2017.

Electron Microscopy (EM)

Graft from whole mount paraffin embedded blocks from P10 (2-weeks post-transplantation) and P13 (4-weeks post-transplantation) was cored out and processed for EM. Tissue pieces were deparaffinized in xylene, rehydrated in a

series of alcohol and water. The pieces were treated with 2% osmium tetroxide for one hour, rinsed, and dehydrated in alcohol series. A 1:1 mixture of propylene oxide and epon was added for one hour, placed in pure epon solution for 2 hours, embedded in labelled molds and placed in a 70°C oven overnight. One micron sections were cut on glass knives and stained with 1% toluidine blue. Thin sections 60-90nm were cut on a diamond knife (DiATOME, Hatfield, PA, USA) and placed on copper grids. The grids are stained in a saturated solution of aqueous uranyl acetate (2-5%) for one hour at room temperature followed by 12 minutes in lead citrate with a grid stick used for staining. The digital images were taken on a JEOL TEM at 80KV (JEM-1230; JEOL, Peabody, MA, USA).

Statistics

Data are depicted as the mean ± standard error of the mean (SEM). Comparisons were performed using two-tailed Student t-test assuming unequal variance. The significance threshold was set at $p<0.05$.

SUPPLEMENTAL REFERENCES

Allard, J., Li, K., Lopez, X.M., Blanchard, S., Barbot, P., Rorive, S., Decaestecker, C., Pochet, R., Bohl, D., Lepore, A.C., *et al.* (2014). Immunohistochemical toolkit for tracking and quantifying xenotransplanted human stem cells. Regen Med *9*, 437-452.

Cantwell, C.D., Roney, C.H., Ng, F.S., Siggers, J.H., Sherwin, S.J., and Peters, N.S. (2015). Techniques for automated local activation time annotation and conduction velocity estimation in cardiac mapping. Comput Biol Med *65*, 229-242.

Cerqueira, M.D., Weissman, N.J., Dilsizian, V., Jacobs, A.K., Kaul, S., Laskey, W.K., Pennell, D.J., Rumberger, J.A., Ryan, T., Verani, M.S., *et al.* (2002). Standardized myocardial segmentation and nomenclature for tomographic imaging of the heart. A statement for healthcare professionals from the Cardiac Imaging Committee of the Council on Clinical Cardiology of the American Heart Association. Circulation *105*, 539-542.

Chen, V.C., Ye, J., Shukla, P., Hua, G., Chen, D., Lin, Z., Liu, J.C., Chai, J., Gold, J., Wu, J., *et al.* (2015). Development of a scalable suspension culture for cardiac differentiation from human pluripotent stem cells. Stem Cell Res *15*, 365-375.

Chong, J.J., Yang, X., Don, C.W., Minami, E., Liu, Y.W., Weyers, J.J., Mahoney, W.M., Van Biber, B., Palpant, N.J., Gantz, J.A., *et al.* (2014). Human embryonic-stem-cell-derived cardiomyocytes regenerate non-human primate hearts. Nature *510*, 273-277.

Ghugre, N.R., Ramanan, V., Pop, M., Yang, Y., Barry, J., Qiang, B., Connelly, K.A., Dick, A.J., and Wright, G.A. (2011). Quantitative tracking of edema, hemorrhage, and microvascular obstruction in subacute myocardial infarction in a porcine model by MRI. Magn Reson Med *66*, 1129-1141.

Henderson, K.K., Mokelke, E.A., Turk, J.R., Rector, R.S., Laughlin, M.H., and Sturek, M. (2003). Maintaining patency and asepsis of vascular access ports in Yucatan miniature swine. Contemp Top Lab Anim Sci *42*, 28-32.

Laflamme, M.A., Chen, K.Y., Naumova, A.V., Muskheli, V., Fugate, J.A., Dupras, S.K., Reinecke, H., Xu, C., Hassanipour, M., Police, S., *et al.* (2007). Cardiomyocytes derived from human embryonic stem cells in pro-survival factors enhance function of infarcted rat hearts. Nat Biotechnol *25*, 1015-1024.

Laflamme, M.A., Gold, J., Xu, C., Hassanipour, M., Rosler, E., Police, S., Muskheli, V., and Murry, C.E. (2005). Formation of human myocardium in the rat heart from human embryonic stem cells. Am J Pathol *167*, 663-671.

McCall, F.C., Telukuntla, K.S., Karantalis, V., Suncion, V.Y., Heldman, A.W., Mushtaq, M., Williams, A.R., and Hare, J.M. (2012). Myocardial infarction and intramyocardial injection models in swine. Nat Protoc *7*, 1479-1496.

Prowse, A.B., Timmins, N.E., Yau, T.M., Li, R.K., Weisel, R.D., Keller, G., and Zandstra, P.W. (2014). Transforming the promise of pluripotent stem cell-derived cardiomyocytes to a therapy: challenges and solutions for clinical trials. Can J Cardiol *30*, 1335-1349.

Sosa, E., Scanavacca, M., d'Avila, A., and Pilleggi, F. (1996). A new technique to perform epicardial mapping in the electrophysiology laboratory. J Cardiovasc Electrophysiol *7*, 531-536.

Tung, R., Kim, S., Yagishita, D., Vaseghi, M., Ennis, D.B., Ouadah, S., Ajijola, O.A., Bradfield, J.S., Mahapatra, S., Finn, P., *et al.* (2016). Scar voltage threshold determination using ex vivo magnetic resonance imaging integration in

a porcine infarct model: Influence of interelectrode distances and three-dimensional spatial effects of scar. Heart Rhythm *13*, 1993-2002.

Weinberger, F., Breckwoldt, K., Pecha, S., Kelly, A., Geertz, B., Starbatty, J., Yorgan, T., Cheng, K.H., Lessmann, K., Stolen, T., *et al.* (2016). Cardiac repair in guinea pigs with human engineered heart tissue from induced pluripotent stem cells. Sci Transl Med *8*, 363ra148.

Xu, C., Police, S., Hassanipour, M., Li, Y., Chen, Y., Priest, C., O'Sullivan, C., Laflamme, M.A., Zhu, W.Z., Van Biber, B., *et al.* (2011). Efficient generation and cryopreservation of cardiomyocytes derived from human embryonic stem cells. Regenerative Medicine *6*, 53-66.
