# Supplementary material for: Human Embryonic Stem Cell-Derived Cardiomyocytes Regenerate the Infarcted Pig Heart but Induce Ventricular Tachyarrhythmias
Source: Stem Cell Reports. 2019 May 2;12(5):967–81. doi: 10.1016/j.stemcr.2019.04.005 (PMC6524945; doi:10.1016/j.stemcr.2019.04.005)
Supplement: Document S2. Article plus Supplemental Information [file mmc2.pdf]

Stem Cell Reports
Article

OPEN ACCESS

Human Embryonic Stem Cell-Derived Cardiomyocytes Regenerate the Infarcted Pig Heart but Induce Ventricular Tachyarrhythmias

Rocco Romagnuolo,[1] Hassan Masoudpour,[1] Andreu Porta-Sánchez,[2] Beiping Qiang,[1] Jennifer Barry,[3] Andrew Laskary,[1] Xiuling Qi,[3] Stéphane Massé,[2] Karl Magtibay,[2] Hiroyuki Kawajiri,[4] Jun Wu,[4] Tamilla Valdman Sadikov,[1] Janet Rothberg,[5] Krishna M. Panchalingam,[5] Emily Titus,[5] Ren-Ke Li,[2,4,6] Peter W. Zandstra,[5,6] Graham A. Wright,[3,6] Kumaraswamy Nanthakumar,[2,6] Nilesh R. Ghugre,[3,6] Gordon Keller,[1,6] and Michael A. Laflamme[1,2,6,*]

[1]McEwen Stem Cell Institute, University Health Network, Toronto, ON M5G 1L7, Canada
[2]Peter Munk Cardiac Centre, University Health Network, Toronto, ON M5G 2N2, Canada
[3]Schulich Heart Research Program, Sunnybrook Health Sciences Centre, Toronto, ON M4N 3M5, Canada
[4]Toronto General Hospital Research Institute, University Health Network, Toronto, ON M5G 1L7, Canada
[5]Centre for Commercialization of Regenerative Medicine, Toronto, ON M5G 1M1, Canada
[6]University of Toronto, Toronto, ON M5G 1L7, Canada
*Correspondence: michael.laflamme@uhnresearch.ca
https://doi.org/10.1016/j.stemcr.2019.04.005

SUMMARY

Human embryonic stem cell-derived cardiomyocytes (hESC-CMs) show considerable promise for regenerating injured hearts, and we therefore tested their capacity to stably engraft in a translationally relevant preclinical model, the infarcted pig heart. Transplantation of immature hESC-CMs resulted in substantial myocardial implants within the infarct scar that matured over time, formed vascular networks with the host, and evoked minimal cellular rejection. While arrhythmias were rare in infarcted pigs receiving vehicle alone, hESC-CM recipients experienced frequent monomorphic ventricular tachycardia before reverting back to normal sinus rhythm by 4 weeks post transplantation. Electroanatomical mapping and pacing studies implicated focal mechanisms, rather than macro-reentry, for these graft-related tachyarrhythmias as evidenced by an abnormal centrifugal pattern with earliest electrical activation in histologically confirmed graft tissue. These findings demonstrate the suitability of the pig model for the preclinical development of a hESC-based cardiac therapy and provide new insights into the mechanistic basis of electrical instability following hESC-CM transplantation.

INTRODUCTION

Following myocardial infarction (MI), necrotic cardiomyocytes are replaced with non-contractile scar tissue, often initiating heart failure. Currently available treatment options for post-MI heart failure include drugs that slow disease progression but do not reverse damage, mechanical circulatory support with complications including thrombosis, infection and the need for an external power supply, and cardiac transplantation limited by the inadequate supply of donor hearts. This situation has driven intense recent interest in the development of alternative cell-based approaches to achieve cardiac repair. The transplantation of various adult stem cell types has been reported to improve left ventricular (LV) contractile function, but beneficial effects appear to be modest and attributable to indirect mechanisms rather than the generation of new cardiomyocytes (Romagnuolo and Laflamme, 2017). By comparison, cardiomyocytes derived from pluripotent stem cells (PSCs) show stable engraftment in multiple MI models, repopulating the infarct scar with electromechanically-integrated new muscle (Caspi et al., 2007; Chong et al., 2014; Laflamme et al., 2007; Shiba et al., 2012, 2014, 2016; van Laake et al., 2008). In an initial proof-of-concept study, our group showed that the transplantation of human embryonic stem cell-derived cardiomyocytes (hESC-CMs) in a rat MI model mediates the partial remuscularization of the infarct scar and has beneficial effects on regional and global LV contractile function (Laflamme et al., 2007). Later, we used a guinea pig MI model and a fluorescent graft-autonomous reporter of graft activation to show that hESC-CM grafts are capable of electromechanical integration and synchronous activation with host myocardium during systole (Shiba et al., 2012).

There have also been more recent efforts to test hESC-CMs and related PSC derivatives in large-animal MI models. Primate ESC-derived multipotent cardiovascular progenitors have been shown to differentiate into multiple cardiac lineages including ventricular myocytes following allotransplantation into infarcted non-human primates (Blin et al., 2010). The Murry laboratory described the successful engraftment of committed cardiomyocytes from hESCs in the infarcted hearts of small macaques (Chong et al., 2014). In the latter study, the authors observed an impressive degree of remuscularization following hESC-CM transplantation, as well as histological evidence of graft cardiomyocyte maturation over time. However, hESC-CM recipients exhibited transient, non-lethal ventricular tachyarrhythmias (VTs) that were

This is an open access article under the CC BY license (http://creativecommons.org/licenses/by/4.0/).

not observed in infarcted monkeys receiving vehicle alone. More recently, Shiba et al. (2016) described qualitatively similar results following the allotransplantation of primate induced PSC-derived cardiomyocytes (iPSC-CMs) in cynomolgus monkeys.

While the field has learned a tremendous amount from the work in the preceding animal models, we reasoned that efforts to develop and translate a safe, effective PSC-based cell therapy would greatly benefit from additional preclinical testing in the infarcted pig heart. The limitations of rodent MI models are widely recognized, and even the aforementioned transplantation work in nonhuman primate models involved relatively small species with substantially different cardiac structure and physiology from humans. *Macaca nemestrina* and *Macaca fascicularis* typically have body weights of ~8 kg and ~3 kg, respectively; and both species exhibit sinus heart rates that exceed those of humans (*Macaca nemestrina*, ~120 beats per minute [bpm]; *Macaca fascicularis*, ~165 bpm; humans, ~60–100 bpm) (Chong et al., 2014; Kato et al., 2014; Malinow et al., 1977; Shiba et al., 2016). Heart rate is a particularly relevant parameter to consider when evaluating electrophysiological outcomes following cell transplantation because recipient species with rapid rates could affect the electromechanical function of implanted human cardiomyocytes and/or mask graft-related arrhythmias that would otherwise occur in slower-rated humans. By comparison, the pig heart's weight-to-body ratio is nearly identical to that of an adult human (~5 g/kg) (Hughes, 1986). The pig heart also has a cardiac structure, sinus rate (~90 bpm), and contractile function that closely resemble that of an adult human (Lelovas et al., 2014). Relative to the primate, the pig also provides significantly greater throughput and reduced experimental costs, and its larger size facilitates better imaging and makes it more amenable to interventions used in adult humans (e.g., catheter-based electroanatomical mapping [EAM]). Given these practical considerations, the pig has been routinely used for the late preclinical testing of novel cardiac interventions.

With this in mind, we hypothesized that hESC-CM transplantation into the infarcted hearts of suitably immunosuppressed pigs would result in their stable engraftment and the partial remuscularization of the infarct scar with outcomes comparable with that seen in other smaller, preclinical models. While this was a feasibility study with primarily histological endpoints, we also examined the functional consequences of hESC-CM transplantation, including LV dimensions and contractile function using cardiac magnetic resonance imaging (MRI), as well as electrophysiological behavior by telemetric electrocardiography (ECG) monitoring and catheter-based EAM and pacing studies.

RESULTS

Scaled Production of hESC-CMs

To generate the requisite number of hESC-CMs for transplantation studies in the pig heart, we employed a highly scalable stirred-tank bioreactor system to expand undifferentiated hESCs and differentiate them into cardiomyocytes (Prowse et al., 2014). Figure 1A depicts the protocol used to induce cardiac differentiation, which was applied to suspension cultures in either 125-mL or 1-L bioreactors. For an initial pilot transplantation study (n = 2 pigs; animal identification numbers P1 and P4), we generated hESC-CMs using the HES-2 hESC line, which resulted in populations with a mean cardiomyocyte purity of 64.3% ± 17.2% and ventricular myocyte purity of 13.3% ± 5.3%, as determined by flow cytometry for the pan-cardiomyocyte and ventricular markers cardiac troponin T (cTnT) and myosin light chain-2v (MLC2v), respectively. For subsequent transplantation experiments, we switched to the ESI-17 hESC line (Crook et al., 2007), which was originally derived under good manufacturing practice conditions but was here differentiated under research-grade standards. ESI-17 hESC-CM populations had an average cTnT purity of 86.3% ± 0.8% and MLC2v purity of 36.3% ± 5.7% (Table S1). For transplantation studies using cardiomyocytes from both lines (mean cTnT purity of 81.9% ± 3.9%), hESC-CMs were cryopreserved prior to transplantation and then thawed at high viability (80.7% ± 2.4% live cells).

hESC-CMs Form Stable Grafts in Injured Pig Hearts

Figure S1 depicts the sequence of experimental procedures employed in the pig MI model. In brief, to test the hypothesis that hESC-CMs will stably engraft in the infarcted pig heart, we induced MIs in adult pigs by 90-min occlusion of the mid-left anterior descending (LAD) coronary artery via percutaneous balloon dilation catheter, followed by reperfusion. At 3 weeks post MI, pigs underwent a small left lateral thoracotomy and direct transepicardial delivery of either 1×10^9 hESC-CMs in pro-survival cocktail (n = 7) or vehicle alone (pro-survival cocktail alone; n = 7). To prevent graft cell rejection, we pharmacologically immunosuppressed all animals 5 days prior to transplantation until euthanasia at either 2 or 4 weeks post transplantation. Details regarding the specific cell populations implanted in each animal are listed in Table S1.

All recipient hearts were then transversely sectioned at uniform intervals (Figure S1C) and subjected to histomorphometry to assess the extent of the infarct scar, as well as the size, composition, and distribution of the resulting graft. Infarct size (as determined by aniline blue

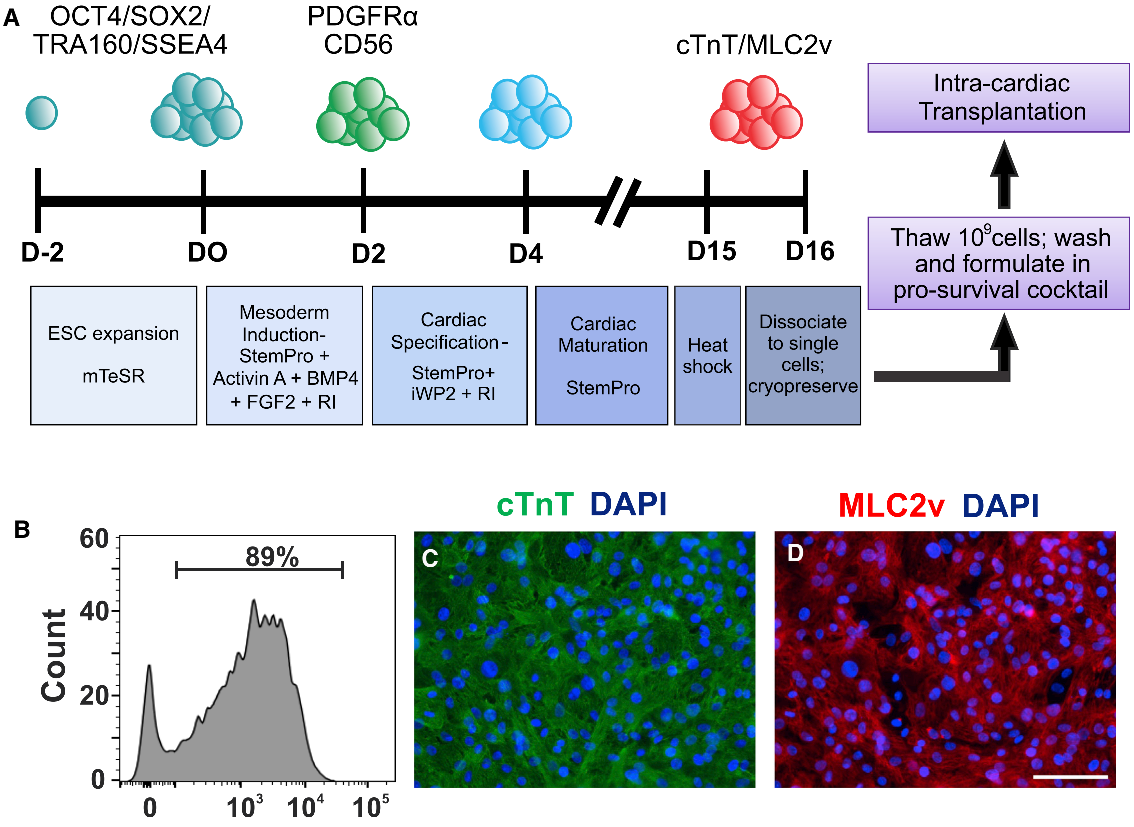

Figure 1. Generation and Phenotype of hESC-CMs for Transplantation Studies

(A) Protocol for hESC-CM production in stirred-tank bioreactors. After expansion in the undifferentiated state, hESC aggregates were differentiated in suspension culture using a staged differentiation protocol. Cell populations were routinely monitored by flow cytometry for markers of pluripotency (OCT4, SOX2, TRA160, and SSEA-4), mesodermal progenitors (PDGFRα and CD56), and committed cardiomyocytes (cardiac troponin T [cTnT] and MLC2v). hESC-CM cultures were transiently heat-shocked after 15 days of *in vitro* differentiation, dispersed to single cells on day 16, and cryopreserved. Immediately prior to transplantation, cells were thawed, washed, and resuspended in a pro-survival cocktail.

(B) cTnT flow cytometry plot for a representative preparation of ESI-17 hESC-CMs.

(C and D) Immunostaining for cTnT (C; green) and MLC2v (D; red) with a nuclear stain (DAPI; blue) on representative fields of ESI-17 hESC-CMs.

Scale bar, 100 μm.

staining on two whole-mount sections taken at 5 and 15 mm from the apex) was comparable in both cell recipients and vehicle controls (34.0% ± 5.8% versus 25.6% ± 6.6% of LV area at 4 weeks post MI, respectively; p = 0.35). hESC-CM recipients showed graft myocardium that occupied a mean of 15.2% ± 3.4% of the scar area (Table S1). hESC-CM graft was composed of irregularly contoured islands of myocardial tissue distributed widely throughout the scar that immunostained with the cardiac marker sarcomeric myosin heavy chain (sarcMHC) and the human-specific nuclear marker Ku80 (Figures 2A and S2). Individual graft implants ranged from a few mm^2 to >90 mm^2 in size. A comparison of hESC-CM graft structure at 2 versus 4 weeks post transplantation indicates that grafts at the later time point have greater sarcomeric content, organization, and alignment as determined by α-actinin staining (Figures 2B and 2D) and electron microscopy (Figures 2C and 2E). These findings are consistent with the *in vivo* graft maturation noted in prior transplantation work in smaller recipient species (Chong et al., 2014; Laflamme et al., 2005).

The immunophenotype of graft tissue (from cardiomyocytes derived from HES-2 and ESI-17 lines) was examined using a wide variety of cardiac and non-cardiac markers. In addition to expressing sarcMHC and α-actinin, hESC-CM graft tissue immunostained strongly for expected sarcomeric markers including cTnT, titin, MLC2v, and MLC2a (Figures 3A–3D). Interestingly, although our cardiac differentiation protocol was expected to yield an admixture of cardiac subtypes (Protze et al., 2016) and only approximately one-third

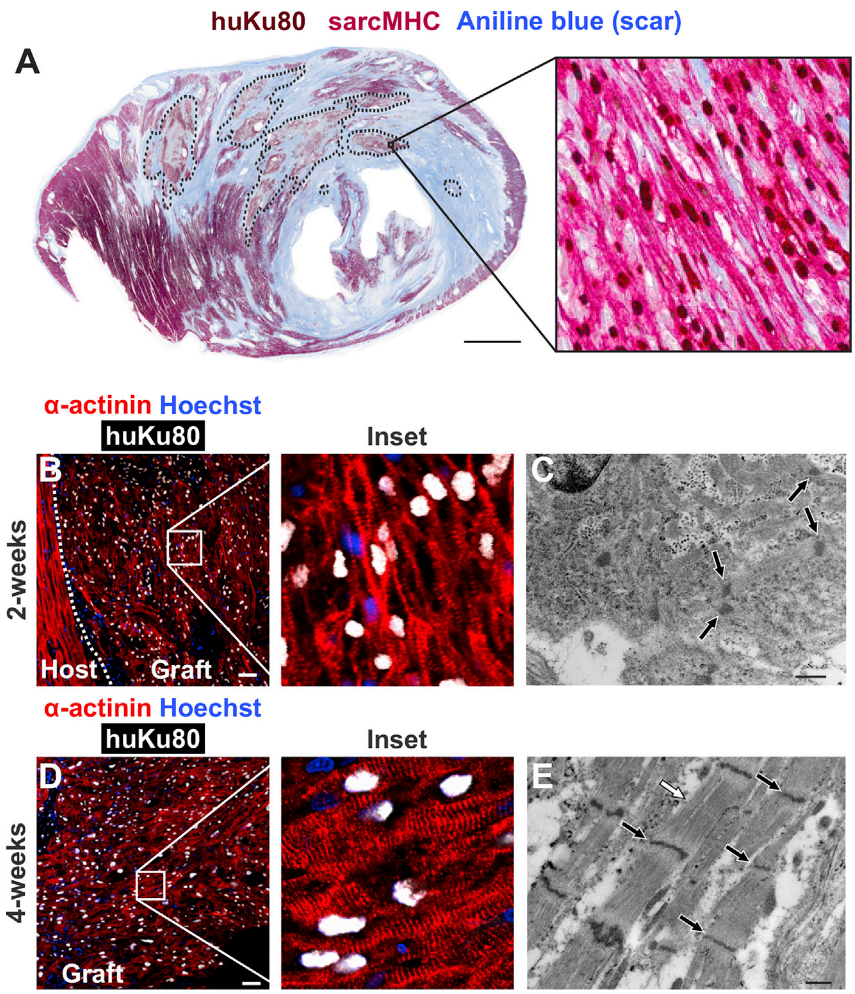

Figure 2. hESC-CMs Partially Remuscularize the Infarct Scar and Mature over Time

Engrafted hearts were transversely sectioned from apex to base, then whole-mount sections were analyzed by immunohistochemistry to identify host and graft myocardium and their relationship to the scar.

(A) Representative cross-section through hESC-CM recipient heart at 14 days post transplantation showing substantial remuscularization of the infarct scar (blue) by human myocardium (dotted lines). Both human and graft myocardium expressed sarcomeric myosin heavy chain (sarcMHC, red), and grafts cells were identified by immunostaining for human-specific Ku80 (brown nuclei). Scale bar, 5 mm.

(B–E) Graft myocardium showed evidence of structural maturation by immunohistochemistry (B and D) and ultrastructure (C and E) with increasing sarcomeric organization and alignment from 2 to 4 weeks post transplantation (B and C versus D and E, respectively). (B and D) White, human-specific Ku80; red, α-actinin; blur, nuclear marker (Hoechst). Scale bar, 50 μm. (C and E) Black arrows, Z-lines; white arrow, M-bands. Scale bar, 0.5 μm.

of cells expressed ML2Cv prior to transplantation (Table S1), the vast majority of the surviving graft cardiomyocytes were MLC2v positive (>90%), and only rarely scattered individual MLC2a-positive graft cells were identified (Figures 3C and 3D). Consistent with prior transplantation studies in other species (Chong et al., 2014; Laflamme et al., 2005), we found that hESC-CM graft tissue at both the 2- and 4-week time points expressed relatively low levels of the cardiac gap junction protein connexin-43 (Cx43). Cx43 immunoreactivity was largely limited to occasional areas at the periphery of the graft or near points of host-graft contact in the border zone (Figures 3E and 3G). By contrast, hESC-CM grafts at both time points strongly expressed the myocardial adherens junction protein N-cadherin (Figures 3F and 3H). We found additional histological evidence of graft maturation in 4-week-old versus 2-week-old graft tissue, with the former demonstrating increased expression of cardiac troponin I, stronger expression and more efficient sarcomeric organization of slow-skeletal troponin I (ssTnI), and increased expression and enhanced subcellular localization of the T-tubule-associated protein caveolin-3 (Figures 3I–3N). There was also a trend toward increased sarcomere length, although this did not reach statistical significance (Figure 3O).

No teratomas were identified in hESC-CM recipients by routine stains (H&E), and the graft cells were uniformly negative for tested endodermal (α-fetoprotein$^+$) or neuronal (β−tubulin III$^+$) markers (Figures S3A and S3B). Grafts were probed with other cell-type-specific antibodies to detect the presence of various non-cardiac elements. In brief, greater than 80% of the human nuclei could be accounted for by the cardiac marker cTnT, but small fractions of endothelial (5.1% ± 2.4% CD31$^+$), fibroblastic (14.3% ± 7.1% TE-7$^+$), and epithelial (0.5% ± 0.2% pan-cytokeratin$^+$) graft cells (5.1% ± 2.4%) were identified (Figures 4A–4E; from n = 6 engrafted hearts analyzed). Moreover, if the two initial animals receiving HES-2-derived populations of lower cardiomyocyte purity are excluded from the analysis, graft composition in the remaining ESI-17 recipients (n = 4) improves to 91.1% ± 1.8% cardiomyocytes, 3.1% ± 1.0% fibroblasts, 0.16% ± 0.12% epithelial cells, and 1.6% ± 0.9%

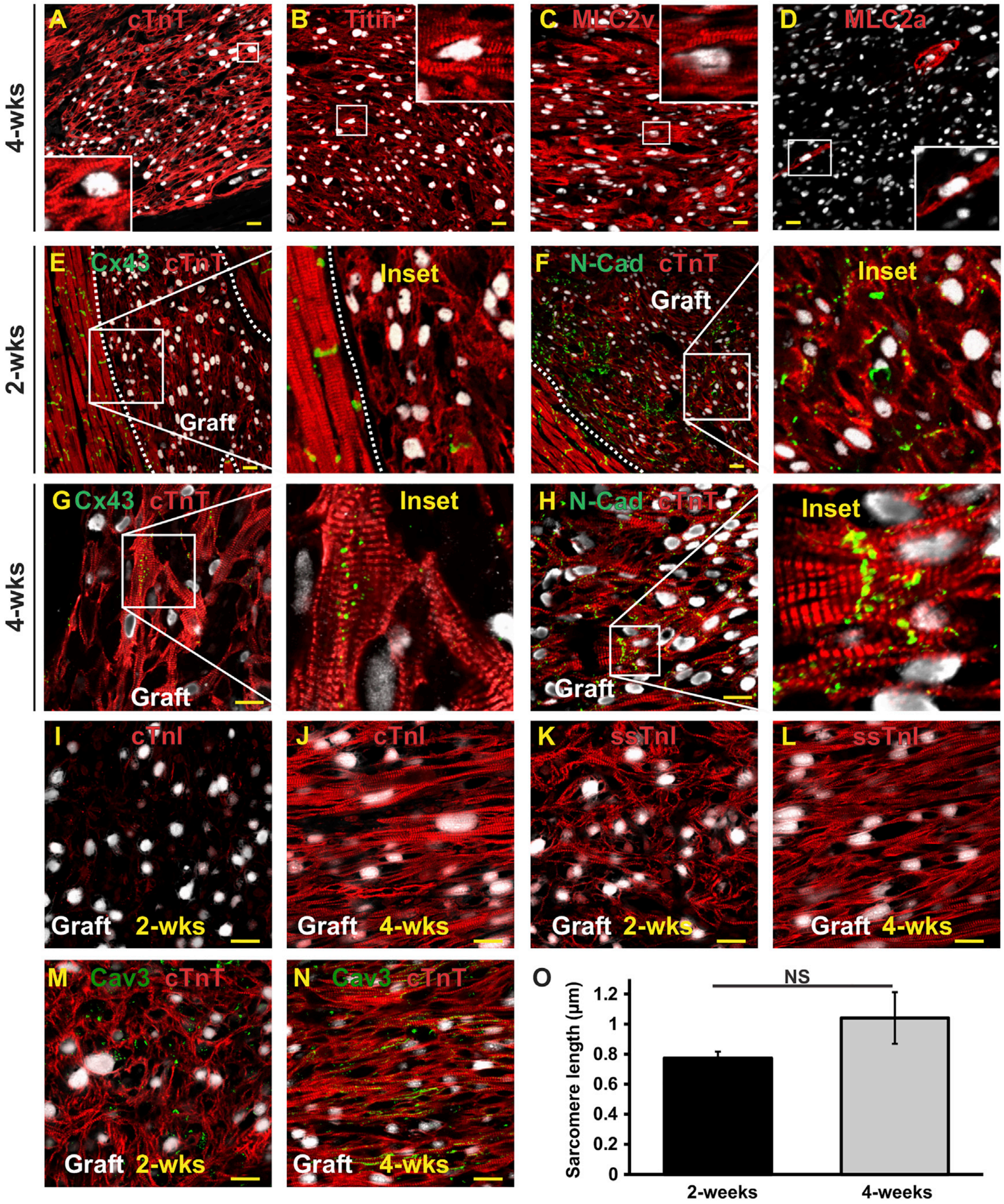

Figure 3. hESC-CM Graft Tissue Expresses Expected Cardiac Markers

(A–N) Confocal immunofluorescence of engrafted hearts at either 4 weeks (A–D, G–J, L, and N) or 2 weeks (E, F, I, K, and M) post transplantation. In all images, graft cells were identified by dual-labeling for human-specific Ku80 (white nuclei). (A–D) hESC-CM graft myocardium was uniformly positive for cardiac troponin T (cTnT; A) and titin (B), largely positive for MLC2v (C), and included only scattered MLC2a-positive cells (D). (E–G) Graft myocardium exhibited patchy and low-level expression of the gap junction protein connexin-43 (Cx43, green), but expressed abundant N-cadherin (N-Cad, green) (E and F at 2 weeks and G and H at 4 weeks). (I–N) Graft myocardium showed evidence of structural maturation over time (2 versus 4 weeks post transplantation) as identified by better organized sarcomeres and enhanced expression of maturation markers including cardiac troponin I (cTnI; I and J), slow-skeletal troponin I (ssTnI; K and L), and the T-tubule-associated protein caveolin-3 (Cav3; M and N). Scale bars in all images, 20 µm.

(O) Comparison of sarcomere length from graft at 2 or 4 weeks post transplantation (n = 3 pigs per time point).

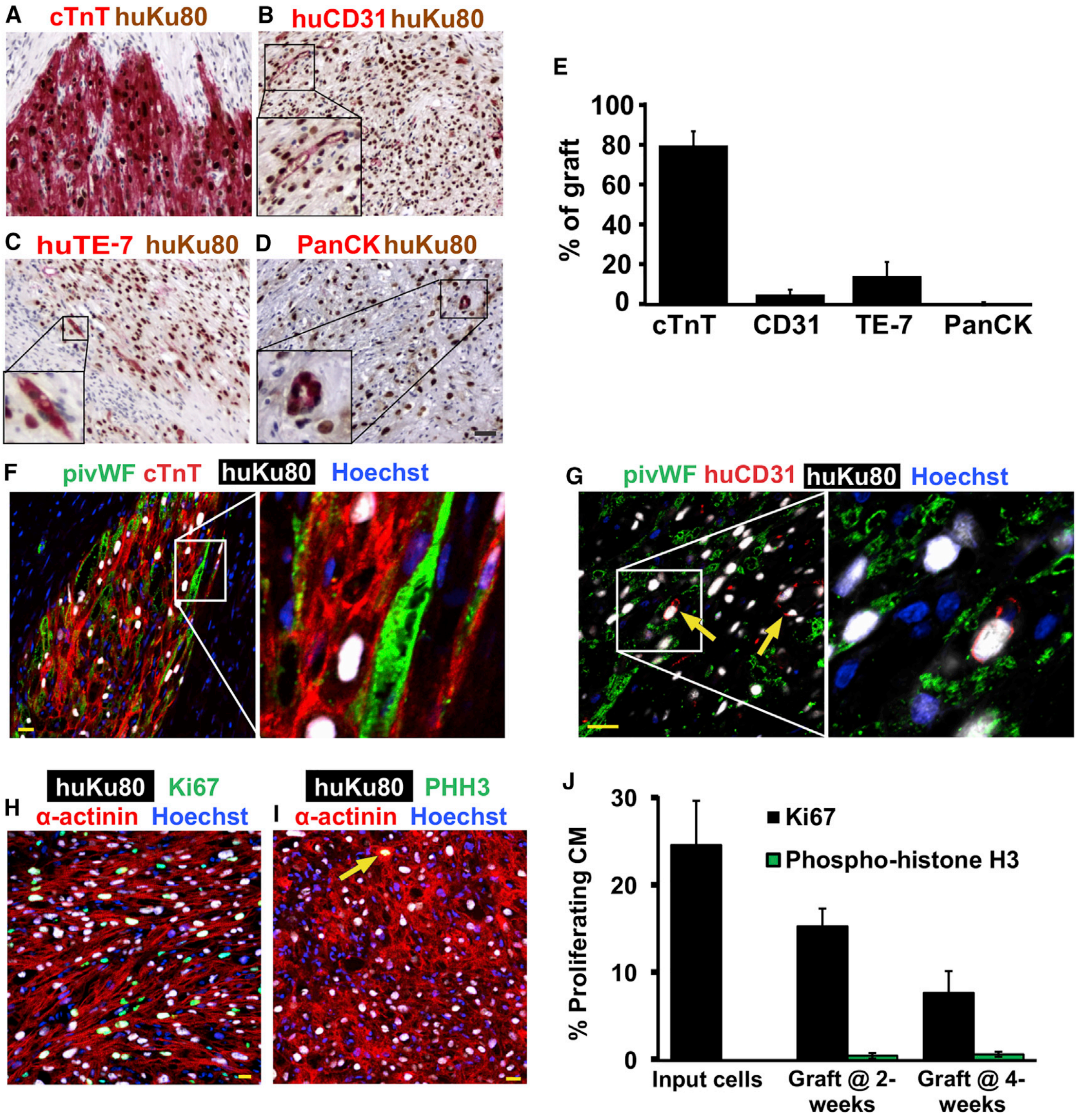

Figure 4. Composition and Proliferation of hESC-CM Grafts

(A–D) To evaluate the cardiomyocyte purity and non-myocyte composition of the grafts, histological sections were dual-labeled for human-specific Ku80 (brown nuclei) and cell-type-specific markers (red) including cardiomyocytes (cTnT; A), human-specific endothelial cells (CD31; B), human-specific fibroblasts (TE-7; C), and epithelial cells (PanCK, pan-cytokeratin; D). Scale bar, 50 μm.

(E) Fraction of Ku80-positive human graft cells immunostaining positively for each of the preceding cell-type-specific markers. n = 6.

(F and G) Host-derived microvessels within the myocardial grafts were identified by confocal immunofluorescence for pig-specific von Willebrand factor (pivWF; green), human-specific Ku80 (white), and (F) cTnT (red) or (G) human-specific CD31 (huCD31; red). Nuclear marker, Hoechst (blue). Arrow indicates a CD31-positive human endothelial cell. Scale bar, 20 μm.

(H–J) For determination of proliferation of graft cells, histological sections were triple-labeled for human-specific Ku80 (white), α-actinin (red), or cTnT (red) and either Ki-67 (H; green) or phospho-histone H3 (PHH3; green) (I). Arrow represents a cTnT, Ku80, and PHH3 triple-positive cell. Scale bar, 20 μm. (J) shows the percentage of graft cardiomyocytes that are either Ki-67$^+$ or PHH3$^+$ at the time of transplantation and at 2 or 4 weeks post transplantation. n = 3 for input cells and n = 6 for graft at 2 and 4 weeks.

endothelial cells. Importantly, we found no obvious correlation between input cardiomyocyte purity and either graft or infarct size by histomorphometry (R^2 values of 0.59 and 0.01, respectively; data not shown).

Because the limited quantity of graft capillary endothelium identified by human anti-CD31 immunohistochemistry seemed unlikely to support these large graft implants, we also immunostained engrafted hearts with an antibody

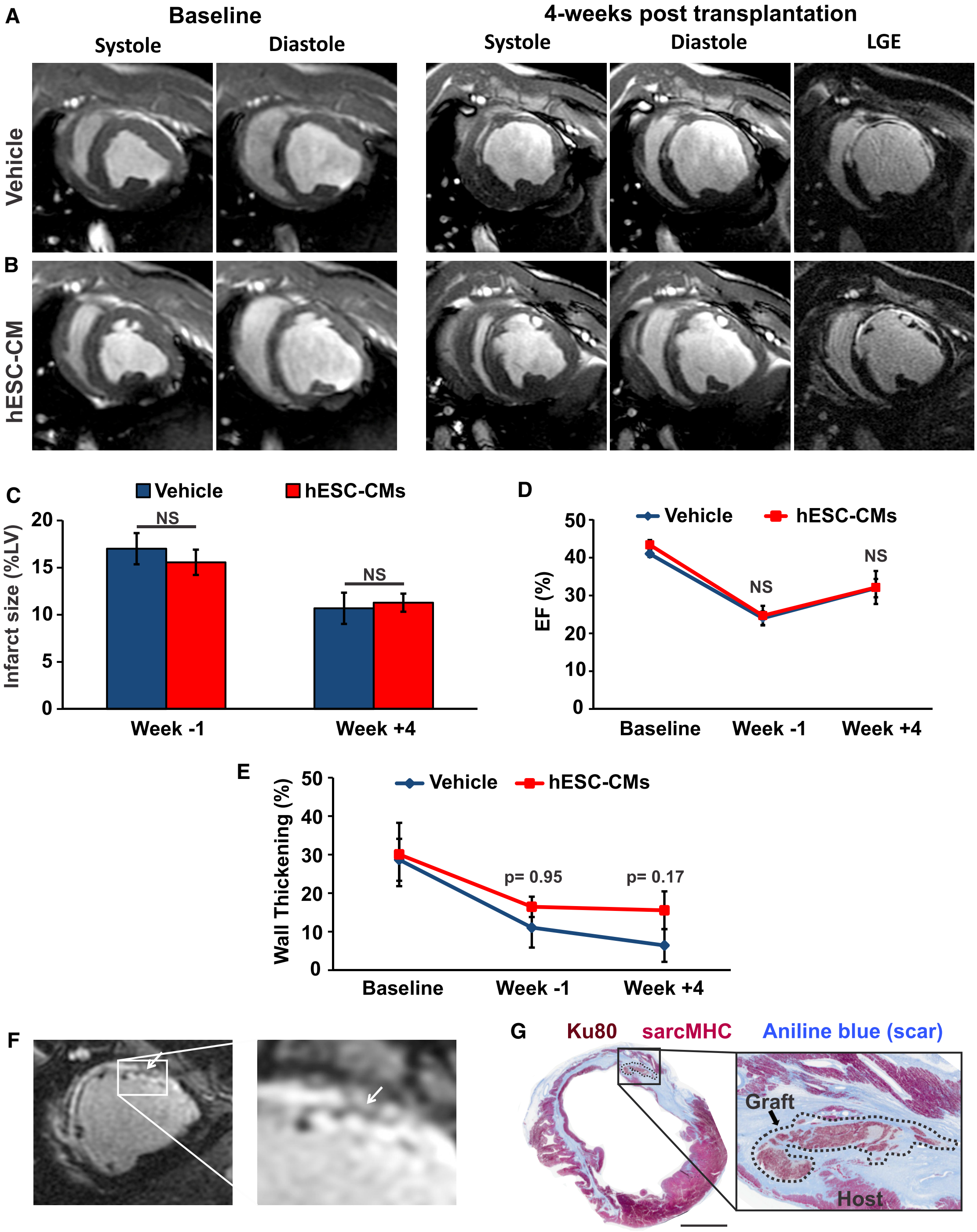

Figure 5. Infarct Size, LV Contractile Function, and Graft Geometry by Cardiac MRI in hESC-CM versus Vehicle Recipients

Infarcted pigs receiving either hESC-CMs or vehicle underwent MRI scans at baseline (prior to MI), 1 week prior to transplantation (2 weeks post MI), and 4 weeks post transplantation (7 weeks post MI).

(A and B) Short-axis slices from representative vehicle (A) or hESC-CM (B) recipients acquired during systole and diastole at either baseline or 4 weeks post transplantation. Rightmost panels also show LGE-MR images to highlight hyperenhanced scar tissue.

(C) Infarct size by LGE-MRI in infarcted pigs receiving hESC-CMs versus vehicle.

(D) LVEF in infarcted pigs receiving hESC-CMs versus vehicle.

(legend continued on next page)

against porcine endothelial cells (pig-specific anti-von Willebrand factor [pivWF]). This immunostaining confirmed a high density of porcine microvessels within the graft (Figures 4F and 4G), indicating a brisk host-derived angiogenic response that likely promotes graft survival and maturation. Of note, the employed anti-piVWF antibody does not cross-react with human cells as evidenced by the lack of overlapping staining seen in histological sections triple-stained with anti-piVWF, a second species-specific antibody against human CD31, and anti-human Ku80 (Figure 4G).

To evaluate the proliferative capacity of hESC-CM graft tissue (from cardiomyocytes derived from HES-2 and ESI-17 lines), we performed dual immunolabeling with human-specific Ku80 and either the pan-cell-cycle marker Ki-67 (Figure 4H) or the mitosis marker phospho-histone H3 (PHH3, Figure 4I). We observed a readily detectable but declining fraction of Ki-67$^+$ graft cardiomyocytes at all time points (24.7% at the time of transplantation, 15.4% at 2 weeks, and 7.8% at 4 weeks post transplantation), as well as rare individual PHH3$^+$ graft cardiomyocytes (Figure 4J). The fraction of Ki-67$^+$ non-myocytes at 4 weeks post transplantation was found to be 6.0% ± 0.8%. To screen for graft cell apoptosis, we also routinely stained sections with an antibody against active caspase-3, but found only vanishingly rare positive cells (<0.1%), suggesting that ongoing graft cell death is not greatly limiting (data not shown).

Finally, we screened for evidence of cellular immune rejection in the hearts of hESC-CM recipients by performing immunohistochemistry with porcine-specific antibodies against pan-leukocyte (anti-CD45), T-lymphocyte (anti-CD3), and B-lymphocyte (anti-CD20) markers. In summary, most graft areas showed either no reaction or sparse infiltration at the graft periphery by host mononuclear cells that were composed almost exclusively of CD3$^+$ T lymphocytes (Figures S3C–S3J). If this immunostaining pattern was graded using criteria applied to human heart allografts (Stewart et al., 2005), it would be best classified as mild cellular rejection, implying adequate immunosuppression with no change in treatment required.

LV Structure and Contractile Function in hESC-CM Recipients by MRI

While this was a feasibility experiment that was neither powered nor designed to demonstrate statistically robust salutary effects on contractile function (especially given relatively small infarcts expected to produce only modest reduction in left ventricular ejection fraction [LVEF]), we nonetheless evaluated infarcted hESC-CM and vehicle recipients by serial myocardial late gadolinium enhancement (LGE) MRI. MRI scans were performed at baseline, 2 weeks post MI, and 4 weeks post transplantation (corresponding to 7 weeks post MI) (see Figures 5A and 5B for representative images). Table S2 details the MRI outcomes by individual animals. As expected, infarct scar size was comparable in both experimental groups prior to cell transplantation (15.6% ± 1.3% in hESC-CM versus 17.0% ± 1.7% in vehicle recipients at 2 weeks post MI; $p = 0.51$), and both groups demonstrated similar reductions in scar size over time (11.3% ± 1.0% in hESC-CM versus 10.7% ± 1.7% in vehicle recipients at 4 weeks post transplantation; $p = 0.77$) (Figure 5C). There was no evidence of an effect of cell transplantation on LV dimensions or global contractile function, and we found no significant differences in LVEF, LV systolic volume, or LV diastolic volume between hESC-CM and vehicle recipients at the 4-week time point or by pairwise comparisons from −1 to +4 weeks post transplantation (Figures 5D and S4A–S4D). On the other hand, while regional wall thickening in the infarcted segment was similar in both experimental groups prior to transplantation ($p = 0.95$), there was a suggestion of improved *regional* myocardial contraction in hESC-CM recipients, as evidenced by a non-significant ($p = 0.17$) increase in dynamic wall thickening of the hESC-CM implanted segment versus vehicle (Figure 5E).

Given the centimeter-scale grafts detected by histology, we hypothesized that larger hESC-CM implants would be visible within the hyperenhanced scar tissue by LGE-MRI. To test this, we used 3D renderings and anatomic landmarks to correlate short-axis LGE-MRI images with whole-mount, transverse histological sections of the engrafted left ventricle taken at the corresponding level. By this approach, we identified numerous instances in which there were islands of non-enhancing tissue with a unique geometry located within the infarct scar that nicely matched with the histologically confirmed graft (Figures 5F and 5G). While there is always some degree of heterogeneity, we did not find similar large non-enhancing regions in infarcted hearts with vehicle alone.

hESC-CM Recipients Exhibit Abundant VT

Infarcted pigs receiving hESC-CMs (n = 6) or vehicle (n = 7) were continuously monitored by telemetric ECG for

(E) Regional wall thickening in infarcted pigs receiving hESC-CMs versus vehicle. The number (n) for (C) to (E) can be found in Table S2.
(F) LGE-MRI images showing strands of non-enhancing tissue identified within the hyperenhanced infarct scar of a representative hESC-CM recipient at 4 weeks post transplantation.
(G) Histological section corresponding to the MR images depicted in (F). Sections were stained to identify sarcMHC (red), human-specific Ku80 (brown), and scar (blue), confirming the presence of human myocardial implants with a geometry matching the strands identified by LGE-MRI. Scale bar, 1 mm.

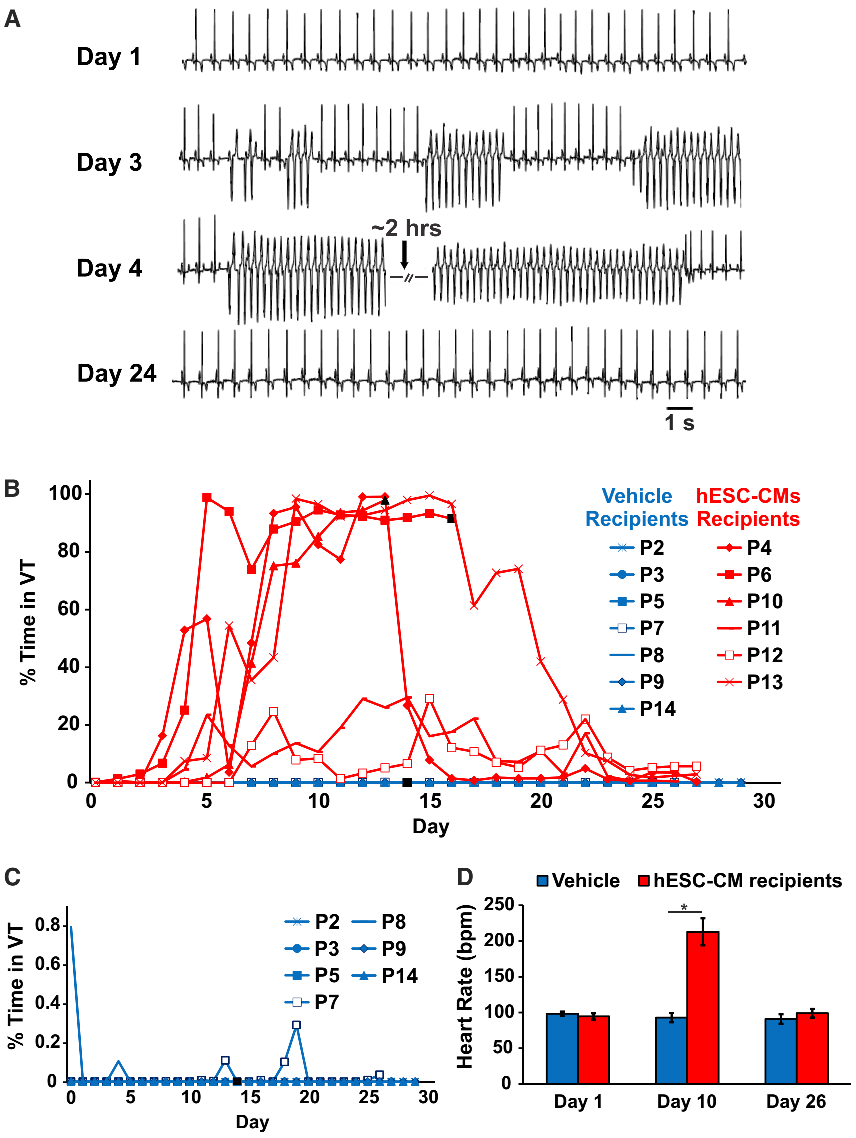

Figure 6. hESC-CM Transplantation Results in Ventricular Tachyarrhythmias

Infarcted animals receiving either hESC-CM or vehicle were continuously monitored by telemetric ECG for spontaneous arrhythmias.

(A) Representative ECG traces from a hESC-CM recipient acquired on days 1, 3, 4, and 24 post transplantation.

(B) Plot depicting the fraction of each day spent in VT in vehicle (blue; n = 7) and hESC-CM (red; n = 6) recipients by time point post transplantation. Pigs that either died or were sacrificed prematurely (P5, P6, and P10) are indicated with a black terminal symbol.

(C) By comparison, vehicle recipients showed either no VTs or rare episodes lasting only a few seconds in duration, so a different ordinate axis is required to visualize VT incidence in these animals.

(D) Mean heart rate in hESC-CM versus vehicle recipients on days 1, 10, and 26 post transplantation. n = 3 for vehicle and hESC-CM recipients. *$p < 0.01$.

spontaneous arrhythmias from the time of thoracotomy until euthanasia. One hESC-CM recipient (P1) was not fitted with a telemetric device but was confirmed to be in sustained VT at the time of euthanasia (2 weeks post thoracotomy). hESC-CM recipients were all initially in normal sinus rhythm, but this group was distinguished by frequent bouts of monomorphic VT that emerged approximately 3–4 days after transplantation and peaked in incidence at approximately 10 days post transplantation (Figures 6A and 6B). By comparison, infarcted pigs receiving vehicle alone showed remarkable electrical stability with rare bouts of VT detected in only 3 of 7 animals, and these events lasted only a few seconds in duration (Figure 6C). During VT, hESC-CM recipients typically showed heart rates in excess of 220 bpm (Figure 6D), and individual VT episodes ranged in duration from a few seconds to >1 h. Indeed, during the window of peak incidence, 4 of 6 hESC-CM recipients were observed to spend an excess of 80% of each day in near-continuous VT. Consistent with prior reports of transient arrhythmogenesis following the transplantation of PSC-derived cardiomyocytes in small monkeys (Chong et al., 2014; Shiba et al., 2016), the incidence of these graft-related arrhythmias gradually decreased over time, such that all hESC-CM recipients returned to normal sinus rhythm by 4 weeks post transplantation. Two hESC-CM recipients (animals P11 and P12) exhibited somewhat less frequent arrhythmias throughout (with a peak VT incidence of ~30% of each 24-h recording period at 2 weeks post transplantation). One of these animals (P11) had the smallest amount of hESC-CM graft by histomorphometry, but the other (P12) had one of the largest grafts (Table S1). Although limited by the relatively small number of animals

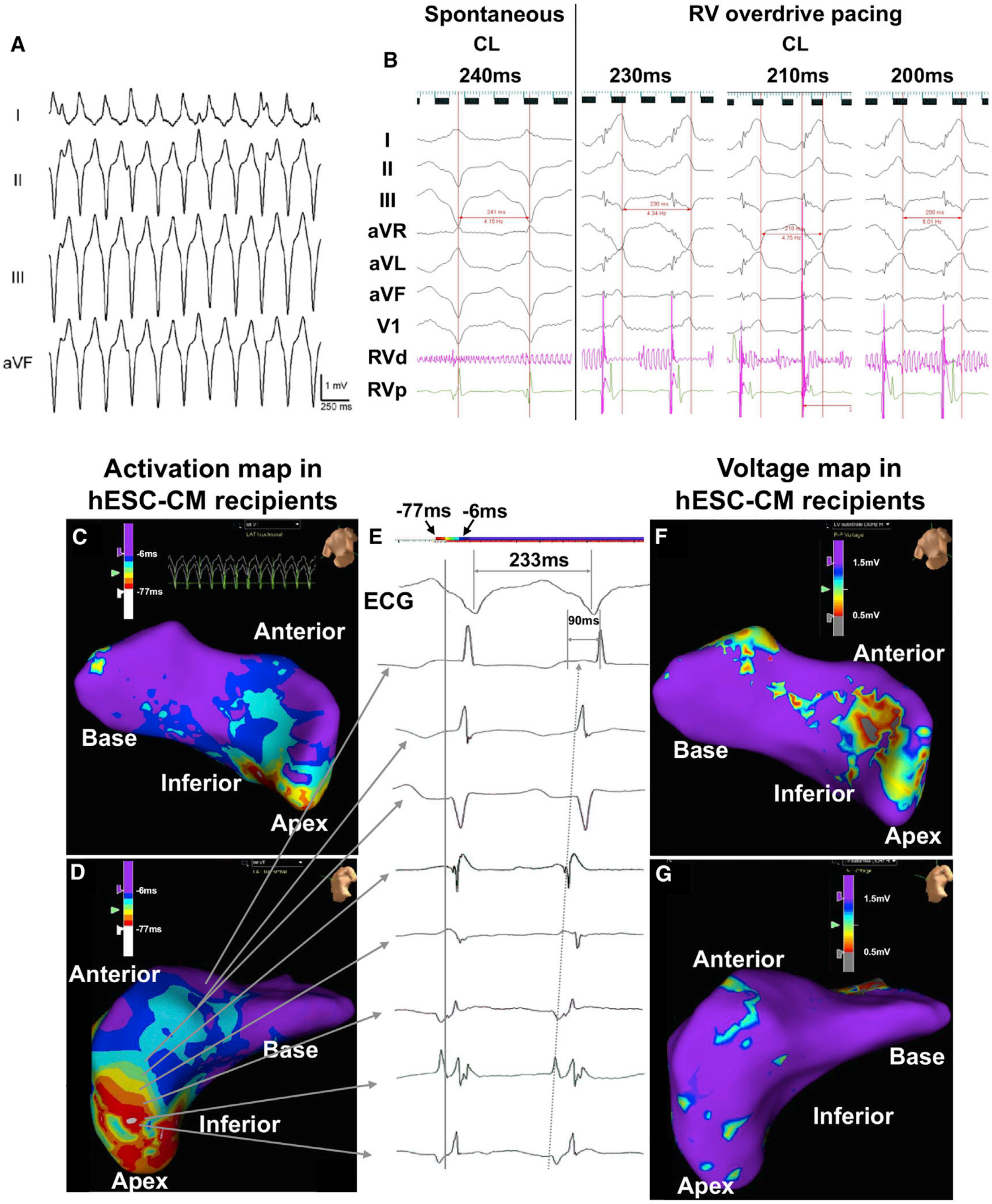

Figure 7. Electrophysiological Studies in hESC-CM Recipients during VT

Infarcted pigs underwent EAM and standard clinical electrophysiological studies 10 days after delivery of vehicle or hESC-CMs.

(A) Surface ECG recordings from a representative hESC-CM recipient in sustained monomorphic VT with a rate of 257 bpm and a superior axis, consistent with an origin in the apical LV.

(B) Surface ECG traces acquired from a representative hESC-CM recipient during overdrive pacing from the right ventricular apex at varying pacing CLs. Note that the QRS complex of the paced beats always shows a fully paced morphology despite pacing at different CL with no evidence of progressive fusion or entrainment. After cessation of pacing maneuvers, the tachycardia continued with variable post-pacing intervals (not shown).

(C and D) Septal (C) and anterolateral apical (D) views of an LV endocardial LAT map acquired from an infarcted hESC-CM recipient (P19) during VT displaying focal activation pattern with no evidence of macro-reentry. Areas of earliest activation are marked by white and red, while later-activating areas are depicted in a gradient from orange to yellow to green to blue.

(legend continued on next page)

enrolled, we found no definite correlation between arrhythmia burden (i.e., total number of hours spent in VT) and either graft size ($R^2 < 0.01$) or cardiomyocyte purity ($R^2 = 0.03$) (data not shown).

While animals with the preceding graft-related arrhythmias were generally stable from a clinical veterinary standpoint despite no administration of anti-arrhythmic agents or defibrillation, 2 of 7 hESC-CM recipients were lost during the arrhythmogenic period (see Table S2). Cell recipient P6 died on day 16 with sustained VT that degenerated into ventricular fibrillation, while P10 was euthanized on day 13 as per veterinary advice due to complications of heart failure (large infarct) and sustained VT. By comparison, 1 of 7 vehicle recipients (P5) had to be prematurely euthanized due to decompensated heart failure at day 14 (an outcome similar to that of cell recipient P10, which had a comparably sized infarct).

VT in hESC-CM Recipients Driven by Focal Mechanism

To investigate the mechanistic basis of these graft-related tachyarrhythmias, we performed catheter-based EAM and standard clinical pacing studies in infarcted pigs 10 days after intracardiac transplantation of hESC-CMs (n = 3) or vehicle (n = 4 animals). This 10-day time point was selected because it corresponded to the peak incidence of VT during the preceding telemetric ECG studies (Figure 6B). Consistent with our previous observations, infarcted vehicle recipients did not show spontaneous VT during EAM, nor were they induced to sustained monomorphic VT by challenge with programmed ventricular stimulation. While resistant to VT induction, vehicle recipients could be induced to ventricular fibrillation during programmed ventricular stimulation with coupling of three extra stimuli at <200 ms cycle length (CL), after which they could be restored back to sinus rhythm by direct cardioversion. Bipolar high-density endocardial substrate maps acquired during sinus rhythm confirmed the expected area of low voltage (<1.5 mV) corresponding to the apical LV infarct zone, and endocardial local activation time (LAT) maps from these hearts showed the expected pattern of late activation occurring in the infarct area.

By contrast, all three infarcted hESC-CM recipients were already in spontaneous VT at the time of electrophysiological study, although they showed good hemodynamic tolerance throughout the procedure. Two of these animals had sustained monomorphic VT, with P19 exhibiting a superior axis and a rate of 257 bpm (Figure 7A) and P20 exhibiting a superior axis and variable rate of 210–230 bpm. In both of these animals, endocardial LAT maps acquired during VT revealed a focal activation pattern with the site of earliest activation located in the apical LV and occurring approximately 20 ms ahead of the surface ECG (Figures 7C–7E). There was no evidence of macro-reentry either under spontaneous conditions or during entrainment and overdrive pacing protocols. In addition, a voltage map acquired during VT showed a limited area of low voltage located in the inferior apex of the LV (Figures 7F and 7G). Overdrive pacing at varying CLs close to the tachycardia CL resulted in transient overdrive suppression of the VT with manifest fusion and no evidence of entrainment or resetting (Figure 7B). These outcomes are also considered consistent with focal mechanisms rather than macro-reentry.

A third hESC-CM recipient (P21) was found to be in sustained monomorphic VT with an inferior axis and a rate of 261 bpm (rhythm VT1) at the beginning of electrophysiological study, but later spontaneously changed to monomorphic VT with a superior axis and a rate of 242 bpm (rhythm VT2) during the procedure (Figure S5A). The VT1 pattern predominated, and VT2 was only observed transiently. Endocardial LAT maps for VT1 showed a pattern of focal activation originating in the anterior septum at a site later confirmed by correlation of the EAM and 3D LGE-MRI datasets to correspond to the most cranial aspect of the infarct scar (Figure S5B). In this case, pacing resulted in overdrive suppression of the VT1 rhythm with the appearance of a sinus beat, followed by the quick reappearance of VT1 after the cessation of pacing. Multiple pacing maneuvers during either VT1 or VT2 only transiently reverted to sinus rhythm followed by the reappearance of VT1 or VT2 soon after.

While there was no evidence of macro-reentry in the preceding endocardial LAT maps, two infarcted hESC-CM recipients (P20 and P21) were also subjected to epicardial mapping to exclude the possibility of an epicardial reentrant circuit. Again we found no evidence of reentry, but rather only focal activation patterns. Figure S5 demonstrates spontaneous changes in VT morphology and abrupt variations in CL, suggestive of focal automatic behavior and not supportive of a macro-reentrant VT circuit.

(E) Corresponding bipolar electrogram recordings and their respective location on the LV endocardial map of (D). The surface QRS (lead aVF) was used as the timing reference. Note that these local electrograms indicate a relatively wide area of early activation, suggesting a mid-myocardial origin in the apical LV.
(F and G) Septal (F) and lateral (G) views of the corresponding LV endocardial voltage map with normal voltage being defined as >1.5 mV, abnormal as 1.5–0.5 mV, and dense scar as <0.5 mV. Areas of low voltage and localized scar are seen in the anterior apical septum. Note that the anterolateral endocardium shows predominantly normal voltages, indicating that the earliest area of VT activation (origin, D) is located in a region with preserved voltages.

For careful correlation of the outcomes of these electrophysiological studies with tissue structure, each heart was harvested at the completion of the experiment, transversely sliced at 5-mm intervals, and used to prepare whole-mount histological sections, which were stained for sarcMHC, human-specific Ku80, and scar tissue. We then used the LGE-MRI dataset and anatomic landmarks to carefully register the 3D EAM with histology with an accuracy of <5 mm. In all three hearts examined, we identified hESC-CM graft tissue at the site of earliest activation in the LAT maps (Figure S6). To further corroborate this correlation, in 2 of 3 hESC-CM recipients (P20 and P21) we delivered radiofrequency ablation lesions in a manner that allowed us to precisely triangulate to the site of earliest activation by histology (including an epicardial ablation mark adjacent to the area of earliest activation and two endocardial marks at a known distance lateral to it). Here again, we unambiguously confirmed that there was hESC-CM graft tissue precisely at the site of earliest activation (Figure S6). Taken collectively, these findings are consistent with graft-related arrhythmias arising via focal mechanisms at the interface between hESC-CM and host myocardium, with no evidence of a macroscopic reentrant circuit.

DISCUSSION

This study was intended to test the feasibility of intracardiac hESC-CM transplantation in the pig MI model as a critical next step in the preclinical development of these cells. In this work, we successfully demonstrated the stable engraftment of hESC-CMs within the infarct scar of immunosuppressed pigs with minimal cellular rejection and no evidence of teratomas. While we obtained a large degree of remuscularization with surviving myocardial implants approximating a cubic centimeter in volume, porcine hESC-CM recipients exhibited frequent graft-related tachyarrhythmias that were similar in nature but qualitatively worse than those previously reported following the transplantation of PSC-CMs in smaller, faster-rated monkeys (Chong et al., 2014; Shiba et al., 2016). Next, we took advantage of the larger size of the pig heart to perform comprehensive catheter-based electrophysiological investigations and found that the graft-related arrhythmias in hESC-CM recipients arise from focal mechanisms originating at the site of cell implantation, rather than macro-reentry.

Overall, these outcomes in the pig model nicely complement prior work in the field testing hPSC-CMs in rodent and large-animal MI models. In early work, our group and others demonstrated that hESC-CMs can partially remuscularize the infarct scar and mediate improvements in regional and global LV contractile function in infarcted rat and guinea pig hearts (Laflamme et al., 2007; Shiba et al., 2012; van Laake et al., 2008). More recently, the Murry group reported that the transplantation of 0.75–1 $\times$ 10^9 hESC-CMs in infarcted macaque hearts resulted in stable myocardial grafts that occupied ~10%–40% of the infarct scar and mediated beneficial effects on LVEF (Chong et al., 2014; Liu et al., 2018). Shiba et al. (2016) transplanted 4 $\times$ 10^8 primate iPSC-CMs in infarcted cynomolgus monkey hearts and remuscularized ~16% of the infarct scar with small improvements in LVEF.

Given that the pig heart is approximately 7- to 8-fold larger than that of these non-human primate hearts, it follows that either a larger cell dose or improved graft survival may be required. Here, we transplanted 1 $\times$ 10^9 hESC-CMs in infarcted porcine hearts and formed grafts that occupied ~15% of the infarct scar. While a full dose-response study is needed to define the optimal quantity of hESC-CMs in the human-sized pig heart, our findings suggest that cell retention and the absolute volume of surviving myocardial graft in this species is at least comparable with that reported in small monkeys. Prior to commencing this study, we were aware of unpublished reports by multiple investigators indicating that they were unable to obtain stable engraftment after injection of human cardiomyocytes into the pig heart. We speculate that our better outcomes may reflect our comprehensive and strictly monitored immunosuppression regimen (comprising a calcineurin inhibitor, T cell costimulatory blockade, and corticosteroid) and/or use of other interventions previously shown to enhance cardiomyocyte engraftment in rodents (heat-shock of hESC-CMs prior to cryopreservation and their delivery within a pro-survival cocktail) (Laflamme et al., 2007).

Another important outcome in this study was the observation of frequent VTs in infarcted hESC-CM recipients. Interestingly, while graft-related tachyarrhythmias in the pigs were more frequent and more lethal than those reported following PSC-derived cardiomyocyte transplantation in small, faster-rated monkeys (Chong et al., 2014; Shiba et al., 2016), they showed a similar time course. In the present study, VTs first appeared in hESC-CM recipients at a few days after transplantation and peaked in incidence at approximately 10 days post transplantation, with many animals spending >90% of each day in near-continuous VT. VT incidence gradually dissipated over time, such that all animals were in normal sinus rhythm by 28 days post transplantation. Given that a very similar time course for graft-related arrhythmias has now been observed following PSC-CM transplantation in multiple independent models (Chong et al., 2014; Liu et al., 2018; Shiba et al., 2016), this seems like a robust phenomenon and a definite hurdle to successful translation.

Interestingly, while limited by the number of animals enrolled, we detected no obvious correlation between graft size and arrhythmia vulnerability. One would intuitively

expect there to be some threshold in graft size below which graft-related arrhythmias would not occur, but a full dose-response study will be required to determine the detailed relationship between graft size and arrhythmia risk. We did not observe any obvious correlation between the input cardiomyocyte purity and arrhythmia incidence, nor was there a correlation between cardiomyocyte purity and either graft or infarct size, suggesting that the presence of non-myocytes did not greatly affect these outcomes.

Given our finding of frequent graft-related arrhythmias in porcine hESC-CM recipients, we used EAM and pacing studies to investigate the mechanistic basis of graft-related VT. Prior to this work, we had hypothesized that reentry would be the predominant mechanism. VTs in post-MI hearts are typically driven by reentry (Benito and Josephson, 2012), and in addition immature hESC-CM graft islands with low graft Cx43 expression and slow conduction velocity (Shiba et al., 2012) seem likely to favor the reentrant phenomenon. In contrast to these assumptions, our findings by telemetric ECG monitoring, EAM, and pacing studies all point toward a focal automatic mechanism rather than reentry. While we cannot definitively exclude micro-reentry (i.e., reentrant phenomenon below the spatial resolution of the available mapping approaches), we found no evidence for a macroscopic reentry circuit by endocardial or epicardial EAM in hESC-CM recipients. By telemetric ECG, VT during initiation had the same QRS morphology as subsequent beats, and we typically observed short bouts of self-terminating VTs that preceded long-lasting VTs. Pacing maneuvers from the right ventricle resulted in overdrive suppression of VT with no evidence of fixed fusion, resetting, or entrainment. Taken collectively, this body of electrophysiological data strongly implicates a focal mechanism rather than reentry, a conclusion that was independently reached by Liu et al. (2018) in the macaque model while this paper was under revision.

If correct, this finding of a focal mechanism raises the intriguing possibility that graft-related arrhythmias may be a graft cell-autonomous phenomenon that can be attenuated by enhancing the electrophysiological phenotype of the input cardiomyocytes. In the present study, the transplanted cell populations were relatively immature and composed of only ~30% MLC2v$^+$ ventricular cardiomyocytes. Interestingly, the surviving graft cardiomyocytes at 2 and 4 weeks post transplantation demonstrated >90% MLC2v$^+$ myocytes, an outcome qualitatively similar to that reported in other models (Chong et al., 2014). While this change in immunophenotype post transplantation may reflect preferential retention and/or survival of MLC2v$^+$ cells, it seems more likely that initially MLC2v-null cardiomyocytes later go on to express this more mature ventricular marker, given the improvements in other maturation parameters (e.g., ultrastructure, expression of cTnI and caveolin-3) over this same interval. We speculate that this *in vivo* ventricular maturation may also account for the greatly reduced incidence of graft-related arrhythmias at >3 weeks post transplantation.

In future work, we plan to use the pig MI model to test the hypothesis that the transplantation of an initially more homogeneous population of mature, electrically quiescent ventricular cardiomyocytes (devoid of any pacemaker cells) will help attenuate graft-related arrhythmias. In separate unpublished work in the guinea pig MI model, our laboratory has recently found that more mature cardiomyocyte populations are indeed capable of robust engraftment and form grafts with enhanced electrophysiological function by optical mapping (data not shown). The guinea pig does not predict the graft-related arrhythmias observed in large-animal models, but we are currently in the process of scaling up the manufacture of these more mature myocytes to the large cell quantities required for pig transplantation experiments. Of course, we also plan studies in the pig model to determine the effects of conventional anti-arrhythmic drugs on graft-related arrhythmia incidence.

There are several other critical hurdles to the successful development of a PSC-based regenerative therapy that we intend to address using the highly relevant pig transplantation model described here. Given its higher throughput and greater size relative to non-human primates, the pig is well suited to tackle practical issues related to cell dosing, delivery vehicle, and the timing of administration. We also expect the pig to be particularly helpful in validating alternative routes of hESC-CM delivery. While a recent clinical trial demonstrated the feasibility of the direct surgical implantation of PSC derivatives via an open-chest approach (Menasche et al., 2018), a minimally invasive delivery strategy would obviously be eventually preferred. Image-guided transendocardial injection catheters have been used to successfully deliver other candidate cell types to the pig heart (Strauer and Steinhoff, 2011), but they will need to be tested for compatibility with hESC-CMs. Finally and most importantly, we still need to complete a pivotal efficacy study to test the hypothesis that hESC-CM transplantation will mediate durable, beneficial effects on LV contractile function in the pig MI model. One limitation of the present feasibility study was that we deliberately sought to optimize cardiomyocyte engraftment by inducing relatively small infarct scars (~16% of LV mass). As anticipated, these animals showed a correspondingly small decrement in LVEF, and contractile function typically rebounded in both cell- and vehicle-treated animals by 4 weeks post transplantation. Hence in future work, we intend to test an appropriately powered study involving a larger cohort of animals with bigger infarcts and a longer duration of follow-up to determine whether this regenerative strategy can improve heart function in the pig MI model.

EXPERIMENTAL PROCEDURES

Production of hESC-Derived Cardiomyocytes

Undifferentiated hESCs were expanded and differentiated into cardiomyocytes in suspension cultures using stirred-tank bioreactors and the experimental sequence depicted in Figure 1A. See Supplemental Experimental Procedures for detailed cardiomyocyte manufacturing methods.

Animal Procedures

All animal studies were approved and conducted in accordance with the Animal Care Committee of Sunnybrook Research Institute. Yorkshire pigs (Caughell Farms, ON) underwent MI induction via balloon occlusion of the mid-LAD, followed 3 weeks later by thoracotomy and direct transepicardial injection of hESC-CMs or vehicle. Animals were pharmacologically immunosuppressed with cyclosporine A, methylprednisolone, and abatacept, and underwent telemetric ECG monitoring, serial cardiac MRI, and/or terminal EAM as described in Figure S1 and Supplemental Experimental Procedures.

Histological Studies

Vehicle and hESC-CM-engrafted hearts were uniformly sliced at 5-mm intervals, and whole-mount transverse sections were subjected to immunohistochemical studies as described in Supplemental Experimental Procedures.

SUPPLEMENTAL INFORMATION

Supplemental Information can be found online at https://doi.org/10.1016/j.stemcr.2019.04.005.

AUTHOR CONTRIBUTIONS

R.R. and M.A.L. designed the study, developed the experiments, and wrote the manuscript. R.R. performed all histology. R.R., H.M., and A.L. performed data analysis. A.P.S., S.M., K.M., and K.N. performed EAM studies and subsequent interpretation and analysis. R.R., B.Q., J.B., H.K., J.W., R-K.L., G.A.W., and N.R.G. performed animal procedures. J.B., X.Q., and N.R.G. performed cardiac MRI. T.V.S., J.R., K.M.P., E.T., P.W.Z., and G.K. generated hESC-CMs.

ACKNOWLEDGMENTS

This work was supported by funding from an Ontario Institute for Regenerative Medicine Disease Team award, the Technion-UHN International Center for Cardiovascular Innovation, the McEwen Stem Cell Institute, the Toronto General Hospital Research Institute, the Peter Munk Cardiac Centre, the John R. Evans Leaders Fund/Canada Foundation for Innovation, and the University of Toronto's Medicine by Design initiative, which receives funding from the Canada First Research Excellence Fund. We would also like to thank Lucy Andrighetti for her expertise in EM experiments. M.A.L. is a founding investigator, consultant, and equity holder in BlueRock Therapeutics. G.K. is a scientific founder, consultant, and equity holder in BlueRock Therapeutics.

Received: June 11, 2018
Revised: April 4, 2019
Accepted: April 5, 2019
Published: May 2, 2019

REFERENCES

Benito, B., and Josephson, M.E. (2012). Ventricular tachycardia in coronary artery disease. Rev. Esp. Cardiol. (Engl. Ed.) *65*, 939–955.

Blin, G., Nury, D., Stefanovic, S., Neri, T., Guillevic, O., Brinon, B., Bellamy, V., Rucker-Martin, C., Barbry, P., Bel, A., et al. (2010). A purified population of multipotent cardiovascular progenitors derived from primate pluripotent stem cells engrafts in postmyocardial infarcted nonhuman primates. J. Clin. Invest. *120*, 1125–1139.

Caspi, O., Huber, I., Kehat, I., Habib, M., Arbel, G., Gepstein, A., Yankelson, L., Aronson, D., Beyar, R., and Gepstein, L. (2007). Transplantation of human embryonic stem cell-derived cardiomyocytes improves myocardial performance in infarcted rat hearts. J. Am. Coll. Cardiol. *50*, 1884–1893.

Chong, J.J., Yang, X., Don, C.W., Minami, E., Liu, Y.W., Weyers, J.J., Mahoney, W.M., Van Biber, B., Palpant, N.J., Gantz, J.A., et al. (2014). Human embryonic-stem-cell-derived cardiomyocytes regenerate non-human primate hearts. Nature *510*, 273–277.

Crook, J.M., Peura, T.T., Kravets, L., Bosman, A.G., Buzzard, J.J., Horne, R., Hentze, H., Dunn, N.R., Zweigerdt, R., Chua, F., et al. (2007). The generation of six clinical-grade human embryonic stem cell lines. Cell Stem Cell *1*, 490–494.

Hughes, H.C. (1986). Swine in cardiovascular research. Lab. Anim. Sci. *36*, 348–350.

Kato, A., Tang, N., Borries, C., Papakyrikos, A.M., Hinde, K., Miller, E., Kunimatsu, Y., Hirasaki, E., Shimizu, D., and Smith, T.M. (2014). Intra- and interspecific variation in macaque molar enamel thickness. Am. J. Phys. Anthropol. *155*, 447–459.

Laflamme, M.A., Chen, K.Y., Naumova, A.V., Muskheli, V., Fugate, J.A., Dupras, S.K., Reinecke, H., Xu, C., Hassanipour, M., Police, S., et al. (2007). Cardiomyocytes derived from human embryonic stem cells in pro-survival factors enhance function of infarcted rat hearts. Nat. Biotechnol. *25*, 1015–1024.

Laflamme, M.A., Gold, J., Xu, C., Hassanipour, M., Rosler, E., Police, S., Muskheli, V., and Murry, C.E. (2005). Formation of human myocardium in the rat heart from human embryonic stem cells. Am. J. Pathol. *167*, 663–671.

Lelovas, P.P., Kostomitsopoulos, N.G., and Xanthos, T.T. (2014). A comparative anatomic and physiologic overview of the porcine heart. J. Am. Assoc. Lab. Anim. Sci. *53*, 432–438.

Liu, Y.W., Chen, B., Yang, X., Fugate, J.A., Kalucki, F.A., Futakuchi-Tsuchida, A., Couture, L., Vogel, K.W., Astley, C.A., Baldessari, A., et al. (2018). Human embryonic stem cell-derived cardiomyocytes restore function in infarcted hearts of non-human primates. Nat. Biotechnol. *36*, 597–605.

Malinow, M.R., Hill, J.D., and Ochsner, A.J., 3rd. (1977). Heart rate in caged *Macaca fascicularis*. Effects of short-term physical exercise. J. Med. Primatol. *6*, 69–75.

Menasche, P., Vanneaux, V., Hagege, A., Bel, A., Cholley, B., Parouchev, A., Cacciapuoti, I., Al-Daccak, R., Benhamouda, N., Blons, H., et al. (2018). Transplantation of human embryonic stem cell-derived cardiovascular progenitors for severe ischemic left ventricular dysfunction. J. Am. Coll. Cardiol. *71*, 429–438.

Protze, S.I., Liu, J., Nussinovitch, U., Ohana, L., Backx, P.H., Gepstein, L., and Keller, G.M. (2016). Sinoatrial node cardiomyocytes derived from human pluripotent cells function as a biological pacemaker. Nat. Biotechnol. *35*, 56–68.

Prowse, A.B., Timmins, N.E., Yau, T.M., Li, R.K., Weisel, R.D., Keller, G., and Zandstra, P.W. (2014). Transforming the promise of pluripotent stem cell-derived cardiomyocytes to a therapy: challenges and solutions for clinical trials. Can. J. Cardiol. *30*, 1335–1349.

Romagnuolo, R., and Laflamme, M.A. (2017). Programming cells for cardiac repair. Curr. Opin. Biotechnol. *47*, 43–50.

Shiba, Y., Fernandes, S., Zhu, W.Z., Filice, D., Muskheli, V., Kim, J., Palpant, N.J., Gantz, J., Moyes, K.W., Reinecke, H., et al. (2012). Human ES-cell-derived cardiomyocytes electrically couple and suppress arrhythmias in injured hearts. Nature *489*, 322–325.

Shiba, Y., Filice, D., Fernandes, S., Minami, E., Dupras, S.K., Biber, B.V., Trinh, P., Hirota, Y., Gold, J.D., Viswanathan, M., et al. (2014). Electrical integration of human embryonic stem cell-derived cardiomyocytes in a Guinea pig chronic infarct model. J. Cardiovasc. Pharmacol. Ther. *19*, 368–381.

Shiba, Y., Gomibuchi, T., Seto, T., Wada, Y., Ichimura, H., Tanaka, Y., Ogasawara, T., Okada, K., Shiba, N., Sakamoto, K., et al. (2016). Allogeneic transplantation of iPS cell-derived cardiomyocytes regenerates primate hearts. Nature *538*, 388–391.

Stewart, S., Winters, G.L., Fishbein, M.C., Tazelaar, H.D., Kobashigawa, J., Abrams, J., Andersen, C.B., Angelini, A., Berry, G.J., Burke, M.M., et al. (2005). Revision of the 1990 working formulation for the standardization of nomenclature in the diagnosis of heart rejection. J. Heart Lung Transplant. *24*, 1710–1720.

Strauer, B.E., and Steinhoff, G. (2011). 10 years of intracoronary and intramyocardial bone marrow stem cell therapy of the heart: from the methodological origin to clinical practice. J. Am. Coll. Cardiol. *58*, 1095–1104.

van Laake, L.W., Passier, R., Doevendans, P.A., and Mummery, C.L. (2008). Human embryonic stem cell-derived cardiomyocytes and cardiac repair in rodents. Circ. Res. *102*, 1008–1010.

Stem Cell Reports, Volume *12*

Supplemental Information

Human Embryonic Stem Cell-Derived Cardiomyocytes Regenerate the Infarcted Pig Heart but Induce Ventricular Tachyarrhythmias

Rocco Romagnuolo, Hassan Masoudpour, Andreu Porta-Sánchez, Beiping Qiang, Jennifer Barry, Andrew Laskary, Xiuling Qi, Stéphane Massé, Karl Magtibay, Hiroyuki Kawajiri, Jun Wu, Tamilla Valdman Sadikov, Janet Rothberg, Krishna M. Panchalingam, Emily Titus, Ren-Ke Li, Peter W. Zandstra, Graham A. Wright, Kumaraswamy Nanthakumar, Nilesh R. Ghugre, Gordon Keller, and Michael A. Laflamme

Supplemental Figures

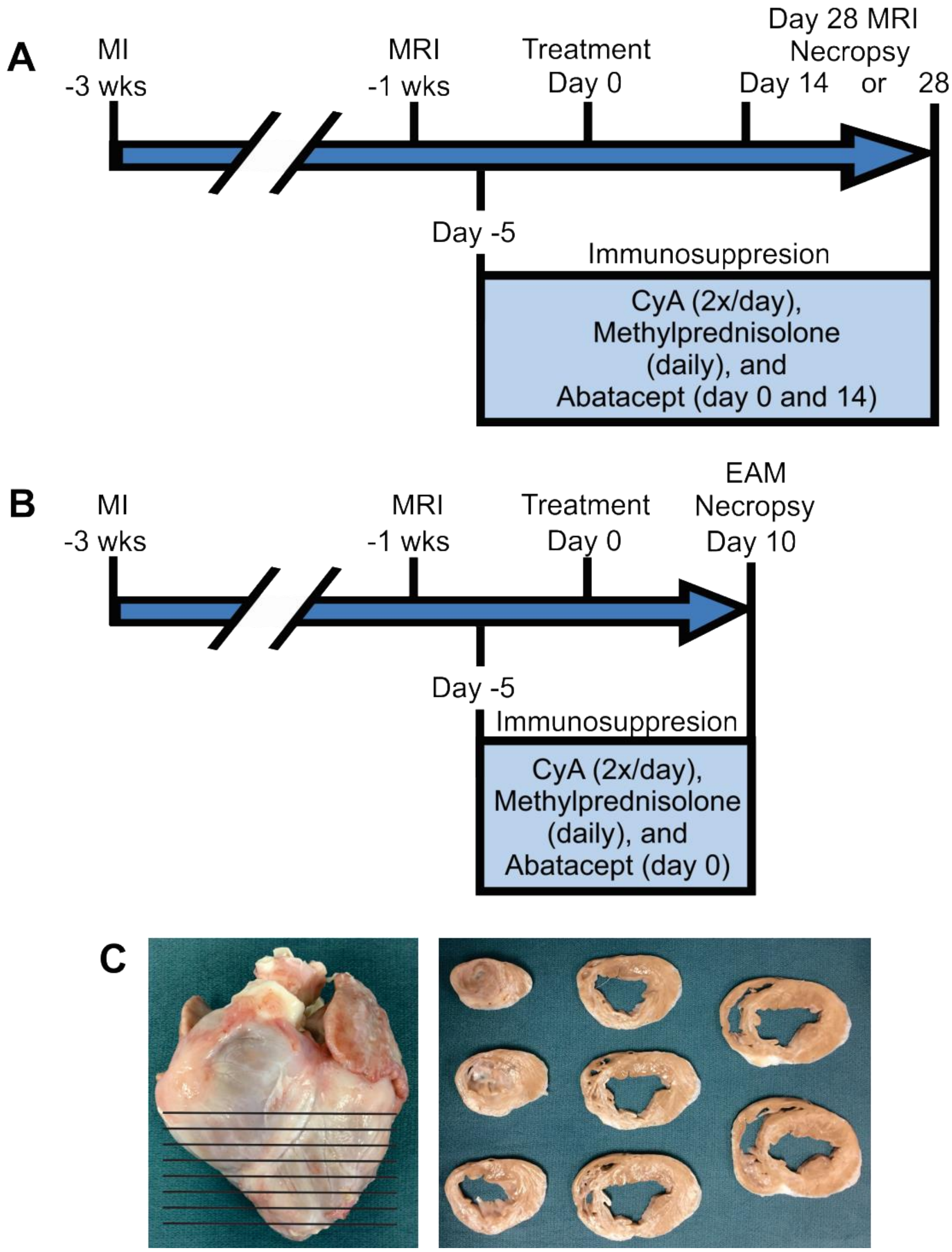

Figure S1. Sequence of experimental animal procedures. Related to Experimental Procedures.
Myocardial infarction (MI) was induced by balloon occlusion 3 weeks prior to direct intramyocardial transplantation of either hESC-CMs or vehicle. **A:** Most pigs were followed for 4 weeks post-transplantation and underwent serial MRI scans at baseline (pre-MI), 1 week prior to transplantation (week -1), and 4-weeks post-transplantation. **B:** A subset of animals were instead subjected to terminal electroanatomical voltage mapping (EAM) and pacing studies at 10 days-post-transplantation. The immunosuppression regime is outlined in **A** and **B**. **C:** Following necropsy, the heart was fixed and sectioned at 5 mm intervals from apex to base, and transverse sections were embedded for whole-mount histology.

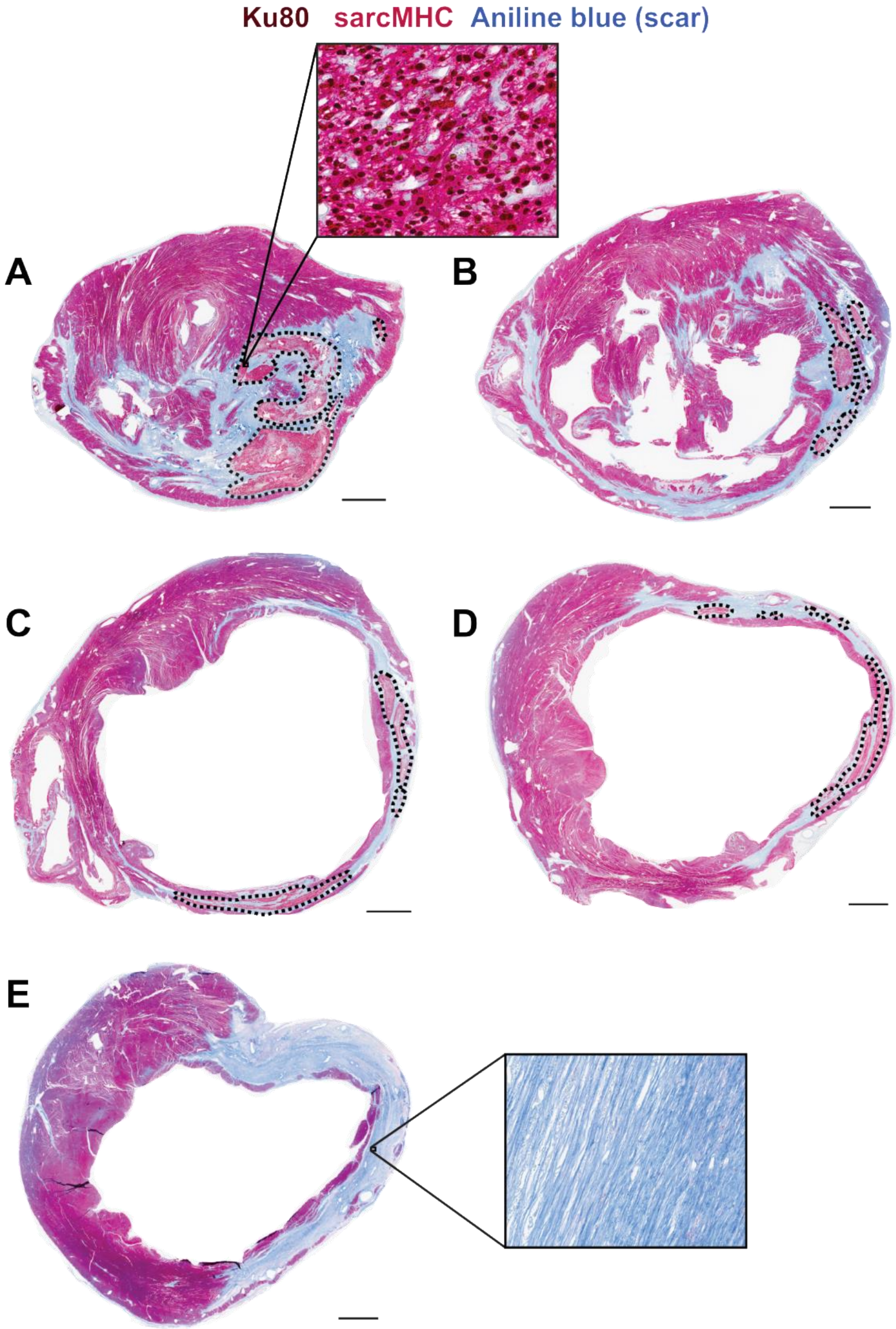

Figure S2. Transverse sections through hearts of representative hESC-CM and vehicle recipients. Related to Figure 2.

A-D: Whole-mount sections of an hESC-CM engrafted heart transversely sliced at 5mm intervals showing abundant human myocardial graft (outlined by dotted lines; A-D). **E:** By contrast, no human-specific Ku80 positive areas were identified in a representative vehicle recipient. Sections from all hearts were stained for sarcomeric myosin heavy chain (red), human-specific Ku80 (brown nuclei), and scar (blue). Scale bar = 5 mm.

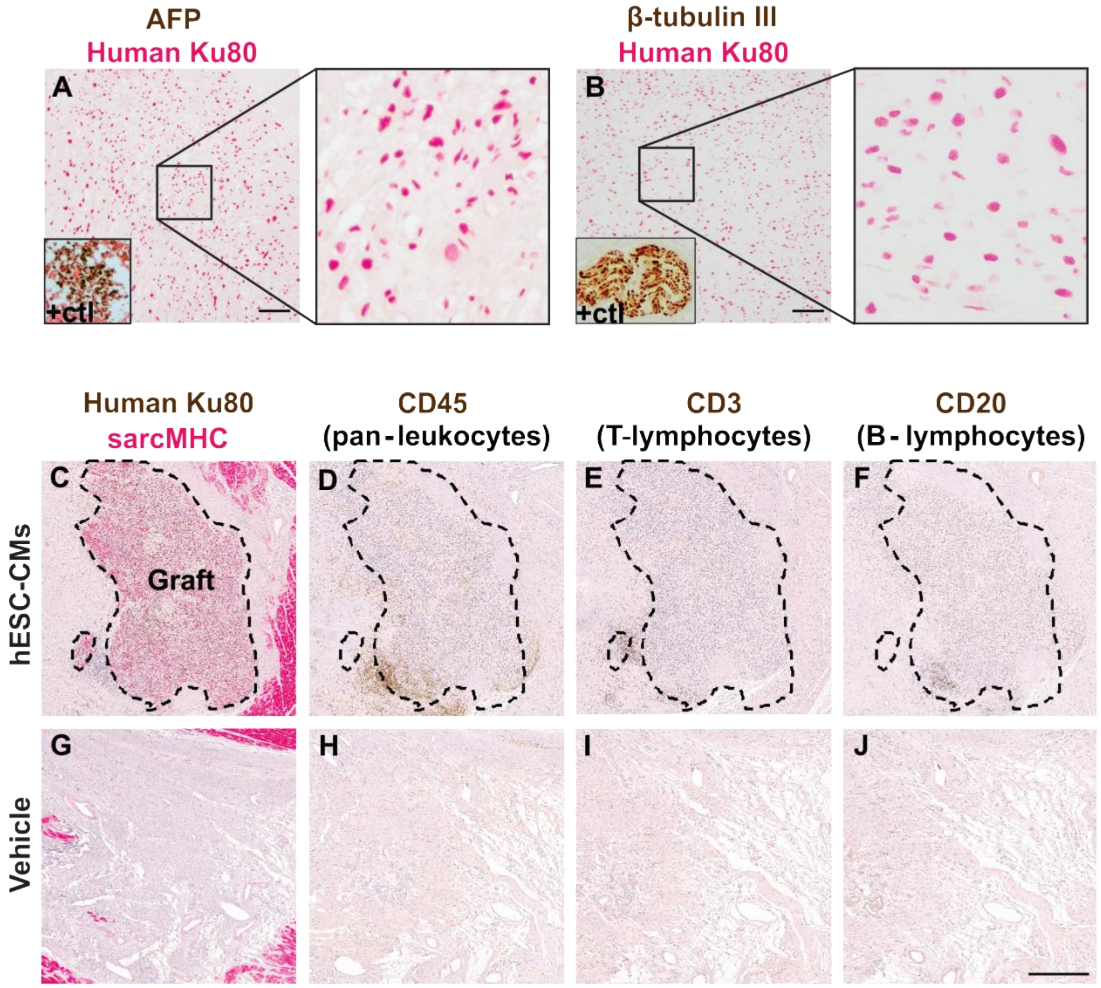

Figure S3. Additional immunohistochemistry to evaluate non-cardiac graft elements and host immune responses. Related to Figures 3 and 4.

A-B: Grafts were uniformly negative for α-1-fetoprotein (AFP, panel **A**) and β-tubulin III (panel **B**), with the insets showing positive controls (day 6 embryoid bodies cultured in 20% FBS or host porcine nerves, respectively). Scale bar = 100 µm. While most hESC-CM graft tissue showed little or no infiltration by host leukocytes, there were occasional grafts that showed evidence of mild cellular rejection. To illustrate this, panels show adjacent histological sections taken from a hESC-CM recipient with a more-than-typical immune response (panels **C-F**) or a vehicle control (panels **G-J**), both at 28-days post-transplantation. **C, G:** Host and graft myocardium were identified by dual-immunostaining for sarcomeric myosin heavy chain (sarcMHC; red) and human-specific Ku80 (brown nuclei). Note the cluster of host mononuclear cells at the lower left-hand corner of the graft in panel **C.** These infiltrates were then evaluated on adjacent histological sections using antibodies against CD45 (pan-leukocytes, **D**, **H**), CD3 (T-lymphocytes, **E**, **I**), and CD20 (B-lymphocytes, **F**, **J**). Scale bar = 500 µm.

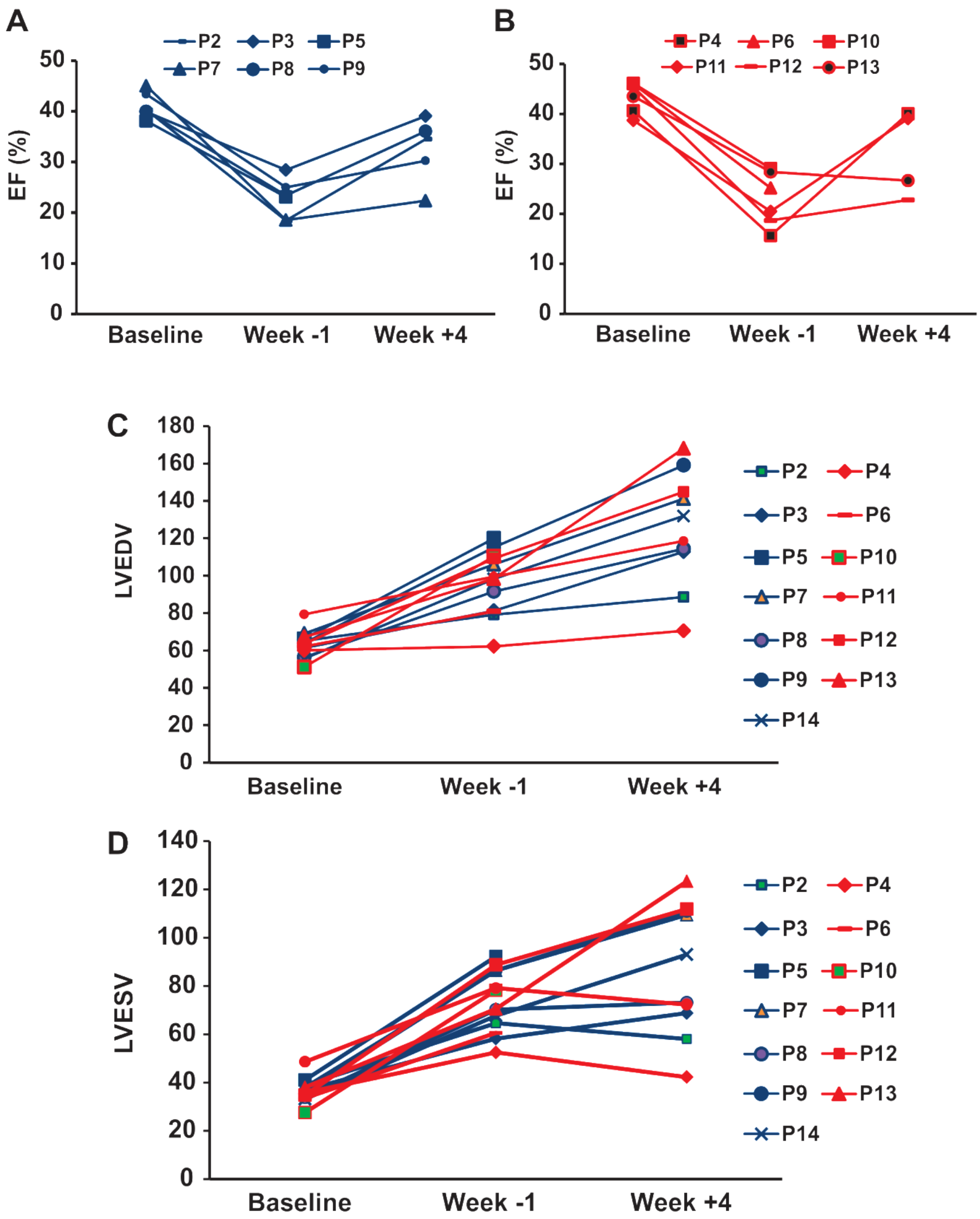

Figure S4. LV dimensions and contractile function determined by cardiac MRI. Related to Figure 5.
A,B: LVEF in infarcted pigs receiving vehicle (**A;** n = 6 pigs) or hESC-CMs (**B;** n = 6 pigs). **C,D**: LVEDV (**C**) and LVESV (**D**) in infarcted pigs receiving vehicle (blue lines; n = 7 pigs) or hESC-CMs (red lines; n = 6 pigs) at baseline, 1-week prior to treatment (week -1), and 4-weeks post treatment (week +4).

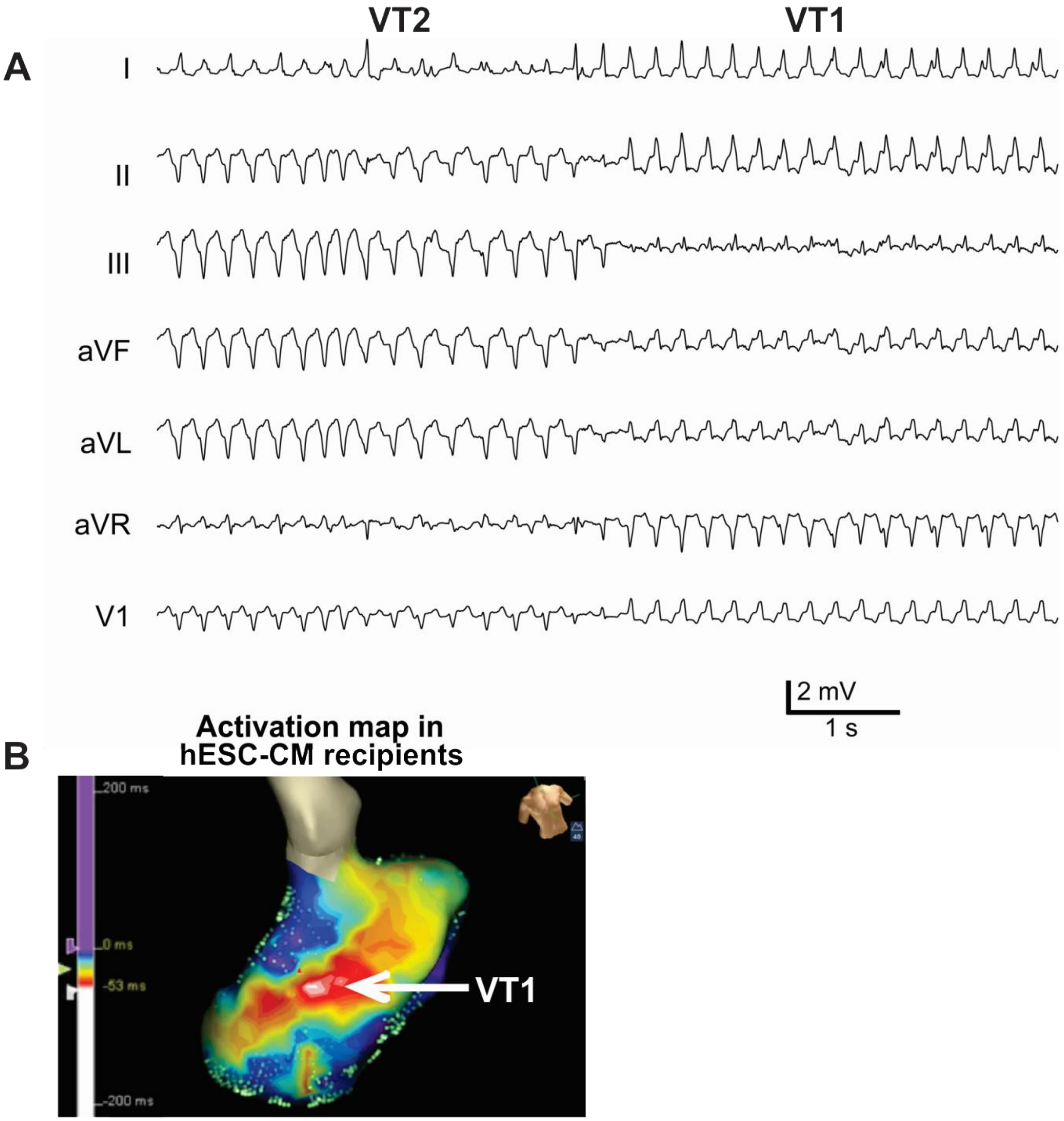

Figure S5. Electrophysiological studies in an hESC-CM recipient with two different VT morphologies. Related to Figure 7.
An infarcted pig (animal P21) underwent EAM and standard clinical electrophysiological studies 10 days following hESC-CM transplantation. **A:** Surface ECG showing monomorphic VT with a superior axis and a rate of 242 bpm (VT2) that showed highly variable activation rates and morphology in transition to an inferior axis and a rate of 261 bpm (VT1). VT1 predominated throughout the procedure. **B:** LAT map acquired during VT1 with VT2 being too transient to fully map. Earliest areas are depicted in white and red and late activated areas are depicted in a gradient from orange to yellow to green to light blue and dark blue.

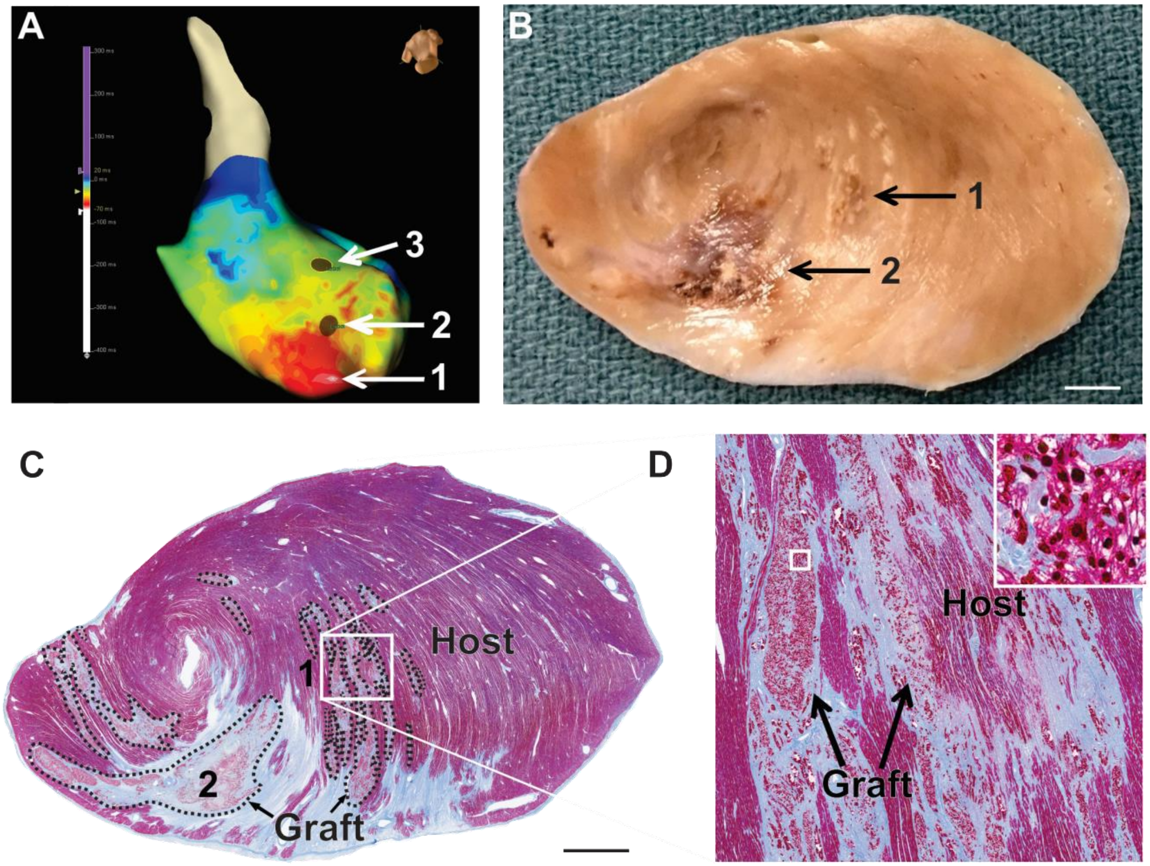

Figure S6. Correlation between EAM and hESC-CM graft location by histology. Related to Figure 7.
A: LAT map from hESC-CM recipient (animal P20) acquired during ventricular tachycardia (VT). Using an ablation catheter, three lesions were applied including one mark on the epicardial surface precisely at the site of earliest activation (denoted as site #1) and two marks on the endocardial surface a known distance lateral to it (sites #2 and 3). **B:** Transverse section of this same heart taken from an apical level corresponding to the site of earliest activation showing grossly discernible ablation marks 1 and 2 (arrows). **C:** Whole-mount histological section corresponding to the image in panel B stained for sarcomeric myosin heavy chain (red), human-specific nuclear marker Ku80 (brown), and scar (blue). Regions of graft tissue located in the scar and border zone are indicated by the dotted lines, along sites of thermal injury and hemorrhage corresponding to ablation marks #1 and 2. **D:** Higher-magnification inset from panel **C** containing a point of contact between host and graft myocardium and representing the site of earliest endocardial activation. Scale bar = 5mm.

Table S1. Summary of pigs receiving hESC-CMs. Related to Figures 2, 3, 4, and 6.

ID	Cell type	ESC-CM purity (cTnT%)	ESC-CM purity (MLC2v%)	Post-Thaw viability	Graft size (% of infarct	VT burden (total hrs)	Graft purity (%cTnT)
**P1**	HES-2	47.1	18.6	92.2	N.D	N.D*	64.2
**P4**	HES-2	81.4	8.0	81.4	29.1	187.8	52.3
**P6**	ESI-17	86.0	68.3	85.4	8.4	254.0	86.3
**P10**	ESI-17	87.1	38.5	81	8.2	123.2	90.6
**P11**	ESI-17	86.7	31.4	72.5	0.1	69.8	N.D
**P12**	ESI-17	87.0	17.1	69.8	17.1	54.3	94.3
**P13**	ESI-17	88.4	41	79.5	7.6	293.9	93.3
**P19**	ESI-17	88.8	34.5	N.D	15.4	N.D**	94.5
**P20**	ESI-17	84.0	41.1	77.2	22.4	N.D**	91.7
**P21**	ESI-17	82.4	18.2	87.3	28.6	N.D**	95.2
	**Avg ±SEM**	**81.9±3.9**	**31.7±5.5**	**80.7±2.4**	**15.2±3.4**	**163.8±40**	**84.8±5.2**

N.D = Not Determined; *P1 was not fitted with DSI telemetry device. At the time of sacrifice, he was found to be in VT; **P19-P21 were used for EAM experiments and only kept to day 10 post-transplantation where they were all in sustained VT.

Table S2: Summary of MRI data on either vehicle or hESC-CMs recipients. Related to Figure 5.

ID	Treatment	Endpoint	Infarct size (%LV)- MRI Week -1	Infarct size (%LV)- MRI Week +4	EF% Baseline	EF% Week - 1	EF% Week +4
**P2**	Vehicle	4-week	11.2	7.7	40.3	18.5	34.5
**P3**	Vehicle	4-week	11.5	5.8	39.9	28.4	39.1
**P5**	Vehicle	Sacrificed day 14	19.6	N.D	38.1	23.1	N.D
**P7**	Vehicle	4-week	21.4	N.D	45.0	18.5	22.3
**P8**	Vehicle	4-week	15.2	13.3	40.0	23.4	36.1
**P9**	Vehicle	4-week	21.3	13.0	43.4	25.0	30.2
**P14**	Vehicle	4-week	18.9	13.7	40.4	31.1	29.5
**P1**	HES-2 CM	2-week	16.6	10.3	N.D	35.3	N.D
**P4**	HES-2 CM	4-week	10.8	9.0	40.6	15.7	40.0
**P6**	ESI-17 CM	Died at day 16	12.6	N.D	46.0	25.2	N.D
**P10**	ESI-17 CM	Sacrificed day 13	19.3	N.D	46.1	29.1	N.D
**P11**	ESI-17 CM	4-week	13.0	12.5	38.7	20.5	39.0
**P12**	ESI-17 CM	4-week	20.2	10.2	45.4	18.7	22.8
**P13**	ESI-17 CM	4-week	16.6	14.4	43.5	28.4	26.7
**P15**	Vehicle	EAVM-day 10	14.5	N.D	42.1	26.5	N.D
**P16**	Vehicle	EAVM-day 10	15.6	N.D	42.4	25.3	N.D
**P17**	Vehicle	EAVM-day 10	N.D	N.D	N.D	N.D	N.D
**P18**	Vehicle	EAVM-day 10	N.D	N.D	N.D	N.D	N.D
**P19**	ESI-17 CM	EAVM-day 10	14.5	N.D	34.5	27.0	N.D
**P20**	ESI-17 CM	EAVM-day 10	10.6	N.D	48.0	31.3	N.D
**P21**	ESI-17 CM	EAVM-day 10	15.8	N.D	43.5	27.8	N.D

SUPPLEMENTAL EXPERIMENTAL PROCEDURES

Production of hESC-derived cardiomyocytes

The scaled expansion of undifferentiated hESCs and their subsequent differentiation into cardiomyocytes was performed by the Centre for Commercialization of Regenerative Medicine (CCRM, Toronto, ON, Canada) using methods adapted from a previously reported stirred-tank bioreactor system (Chen et al., 2015; Prowse et al., 2014). While the vast majority of transplantation studies were conducted using cardiomyocytes generated using the GMP-pedigree ESI-17 hESC line (BioTime, Alameda, CA, USA) (Chen et al., 2015), two pigs (P1 and P4) received cardiomyocytes derived from HES-2 hESC line (BioTime, WiCell, Madison, WI, USA) in an initial pilot study. hESCs from both lines were expanded in the undifferentiated state using mTESR medium (StemCell Technologies, Vancouver, BC, Canada), then guided into cardiomyocytes using the differentiated protocol depicted in **Figure 1A** (Chong et al., 2014). For this, hESC aggregates in either 125 mL or 1L bioreactors were transferred into complete StemPro (Thermo Fisher, Waltham, MA, USA) cardiomyocyte differentiation media containing L-glutamine (2 mM, Thermo Fisher, Waltham, MA, USA), transferrin (150 µg/mL, Sigma-Aldrich, St. Lois, MO, USA), monothioglycerol (MTG; 50µg/mL, Sigma-Aldrich, St. Lois, MO, USA), ascorbic acid (50 mg/mL, Sigma-Aldrich, St. Lois, MO, USA) and ROCK inhibitor Y-27632 (RI; 10 µM, Tocris Bioscience, Oakville, ON, Canada) and then were serially treated with activin A (6ng/mL, R&D, Minneapolis, MN, USA), bone morphogenetic protein-4 (10 ng/mL, R&D, Minneapolis, MN, USA) and basic fibroblast growth factor (5ng/mL, R&D, Minneapolis, MN, USA) for mesoderm induction. Two days post-induction, media was replaced with iWP2 (2 µM, Tocris Bioscience, Oakville, ON, Canada) and ROCK inhibitor (10 µM) in StemPro cardiomyocyte differentiation media for two days, followed by complete media exchange with StemPro cardiomyocyte differentiation media every two days thereafter until harvesting. One day prior to harvesting, on day 16 post-induction, cell aggregates were transiently heat shocked (42° C for 30 minutes) in StemPro cardiomyocyte differentiation media (lacking MTG and ascorbic acid) to improve graft cell survival as previously reported (Laflamme et al., 2007; Laflamme et al., 2005). Twenty-four hours following heat shock, cell aggregates were dispersed enzymatically to single cells with collagenase II (1g/L in HBSS, Worthington Biochemical Corporation, Lakewood, NJ, USA) for 2 hours at 37°C on an orbital shaker, pelleted, resuspended in TrypLE (Life Technologies, Carlsbad, CA, USA) with DNase I (10 µg/mL, EMD Millipore, Etobicoke, ON, Canada), then cryopreserved as previously described (Xu et al., 2011). On the day of transplantation, cells were thawed at 37°C and cell counts and post-thaw viability was determined using the nucleocounter NC-200 automated cell counter (Chemometec, Gydevang, Denmark). The cells were then washed with RPMI-1640 media, and suspended in 3.0 mL of a previously reported pro-survival cocktail (Laflamme et al., 2007) consisting of growth factor-reduced Matrigel (~60% v/v), supplemented with cyclosporine A (200 nM, Sandimmune, Novartis, Mississauga, ON, Canada) and pinacidil (50 µM, Sigma-Aldrich, St. Lois, MO, USA). Cell viability post-thaw was routinely assessed with acridine orange/DAPI staining and quantification with a Nucleocounter NC-200 automated cell counter (ChemoMetec A/S, Allerod, Denmark) (**Table S1**). Grafts at either 2- or 4-weeks post-transplantation were routinely stained with caspase-3 (Cell Signaling Technology, Danvers, MA, USA) to determine the number of apoptotic cells (<0.1% of graft cells were caspase-3 positive).

Animal procedures

All animal studies were approved and conducted in accordance with the Animal Care Committee of Sunnybrook Research Institute. For all procedures, 20-30 kg male Yorkshire pigs (Caughell Farms, Fingal, ON, Canada) were fasted overnight and then anesthetized by intramuscular administration of a cocktail consisting of atropine (0.05mg/kg) and ketamine (33mg/kg), followed by maintenance with 5% inhaled isoflurane. The sequence of experimental animal procedures is depicted in **Figure S1**.

<u>Myocardial infarction:</u> MI induction was performed as previously reported (Ghugre et al., 2011). In brief, animals received a pre-operative bolus of amiodarone (75 mg), lidocaine (20 mg bolus, 3 mg/kg/hour infusion), and heparin (100 IU/kg iv). Under X-ray fluoroscopic guidance (Veradius C-arm System, Philips Healthcare, Markham, ON, Canada) with iodinated contrast, complete occlusion of the mid left anterior descending coronary artery was performed for 90 minutes via inflation of a percutaneous balloon dilation catheter (Sprinter Legend Balloon Catheter, Medtronic, Minneapolis, MN, USA), followed by reperfusion. Pigs showing infarct sizes less than 10% of LV mass at their initial scan pre-transplantation were excluded from the study (n=4 animals).

<u>Immunosuppression:</u> To facilitate the intravenous administration of immunosuppressive drugs and routine blood sampling, all animals (including control and hESC-CMs recipients) underwent placement of an indwelling vascular access port (Access Technologies, Skokie, IL) in the external jugular vein at 2 weeks post-MI (Henderson et al., 2003). All pigs (vehicle and hESC-CM recipients) were treated with an immunosuppression regimen including

Orencia (Abatacept CTLA4 immunoglobulin, Bristol-Myers Squibb, Mississauga, ON, Canada), given at 12.5 mg/kg on day of hESC-CM transplantation and every 2 weeks thereafter); methylprednisolone (Pfizer, Mississauga, ON, Canada), given as 250 mg on day of hESC-CM transplantation followed by a taper to 125 mg per day over two weeks and then 125 mg daily maintenance thereafter); and cyclosporine A (Neoral, Novartis, Mississauga, ON, Canada), given as 10-16 mg/kg PO twice per day to achieve trough concentrations of 250 µg/L, administered from 5 days prior to hESC-CM transplantation daily until sacrifice. Blood was drawn and cyclosporine A was measured 1-2 times per week to ensure adequate trough levels (Toronto General Hospital Laboratory Medicine and Pathology Department).

Thoracotomy and cell implantation: hESC-CMs were directly injected into the infarct scar using a transepicardial delivery approach adapted from prior work in the non-human primate model (Chong et al., 2014). On day 20 post-MI, pigs were anesthetized and underwent left thoracotomy as previously described (McCall et al., 2012). The fourth intercostal space was exposed and opened adjacent to the lower rib, and the space was enlarged under direct vision using a self-retaining rib retractor. After opening the pericardium anteriorly, the apex and the anterior LV are gently exposed using warm saline-wet gauze placed beneath the heart. hESC-CMs in pro-survival cocktail or vehicle alone were then directly injected into the infarct zone using a manually curved 27G needle via 12 injections of 250 µL each. Before closing the chest, a telemetric ECG sensor was implanted as described below.

Telemetric ECG: All animals were continuously monitored from the time of cell transplantation until sacrifice via non-invasive telemetric ECG (Ponemah Physiology Platform, Data Sciences International (DSI), St. Paul, MN, USA). After completion of the thoracotomy, M01 telemetry devices (DSI) were installed with the leads placed within the chest wall adjacent to the base and apex of the heart. Electrocardiograms (ECG) were collected from the implanted device from all animals and telemetrically recorded continuously using Ponemah software (DSI). ECG traces were evaluated with Data Insights analysis software (DSI) and verified manually to determine all incidences of VT, defined as a run of 4 or more premature ventricular complexes (PVCs). Heart rate was measured at two points (12 am and 12pm) on days 1, 10 and 26 for vehicle and hESC-CMs recipients with averages and standard error of the mean being reported.

Cardiac MRI: Parameters including LV dimensions, LV ejection fraction (LVEF) and infarct size were assessed using the gold-standard technique of cardiac MRI. MRI scans were performed at baseline (i.e. prior to MI), at 2-weeks post-MI and at 4-weeks post-transplantation (corresponding to 7-weeks post-MI) using methods as previously reported (Ghugre et al., 2011). In brief, MRI scans were acquired using a 3T MR750 scanner (GE Healthcare, Markham, ON, Canada) and following three planes localization, multiple slices (5 mm thick) through the myocardium were obtained using steady-state free precession sequence in cine mode (FIESTA, GE Healthcare, Markham, ON, Canada) for global LV function. Contrast injection of Gadolinium-DTPA (0.2mg/Kg) was used to quantify infarction/scar size with a T1-weighted IR-FGRE sequence; late gadolinium enhancement (LGE) imaging was performed at 8-10 min post contrast injection. As determined by LGE-MRI, infarcts sizes ranged between 1.2-21.4% of LV mass, and animals showing infarct sizes <10% were excluded from the study (n=4). Regional wall thickness was determined as an average of segments 7 and 8 corresponding to the mid ventricular anterior and anteroseptal myocardium, respectively, as per AHA guideline recommendations (Cerqueira et al., 2002). These segments corresponded to the site of injection for vehicle and hESC-CM transplantation (i.e. the core region of the infarct). Offline analysis of MRI data was carried out using CVI42 software (Circle Imaging, Calgary, AB, Canada).

Electroanatomical mapping: Animals underwent terminal EAM mapping at 10 days post-thoracotomy and intra-cardiac implantation of hESC-CMs or vehicle. Endocardial mapping of the LV was performed via a transaortic retrograde approach using the Abbott Precision™ Research version mapping system and an HD16 grid™ catheter (Abbott Medical, Minneapolis, MN, USA) with 16 1mm-diameter electrodes (4mm center to center electrode spacing arranged in a fixed spaced array). All electrograms were acquired with filtering at 0.5-300Hz allowing for unipolar and bipolar data analysis. A quadripolar catheter was placed in the inferior vena cava and right ventricle (RV) apex as the unipolar reference. Acquisition of electrograms and projection onto the anatomical surfaces was performed with a threshold of 7mm of internal projection and 7mm interpolation. Field scaling was applied for all maps with low voltage areas defined as <1.5mV (Tung et al., 2016). Annotation of timing of bipolar electrograms was taken based on the maximum negative dV/dt of the bipolar signal as described previously (Cantwell et al., 2015). Electroanatomical maps were then registered with the MRI volumes offline using the aorta and the LV surface as fiducial points for better anatomical correlation with the location of the infarct. We also applied radiofrequency (RF) energy to "mark" the tissue at known distances relative to the site of earliest activation to allow for an even more precise correlation between histology and EAM in a subset of animals. For this, RF energy (30

Watts, 30 seconds, temperature controlled to 65ºC) was delivered with a non-irrigated FlexAbility™ Abbott ablation catheter.

In two hESC-CM recipients, epicardial mapping was also performed to rule out the possibility of an epicardial reentrant circuit. For this procedure, we used the same Abbott Precision™ mapping system and gained access via the subxiphoid approach as described previously (Sosa et al., 1996).

After the completion of EAM studies, we used standard clinical electrophysiological maneuvers to either: 1) assess arrhythmia vulnerability in infarcted vehicle controls, all of which were in normal sinus rhythm, or 2) help elucidate the mechanistic basis of spontaneous VT in infarcted hESC-CM recipients, all of which were already in VT at the time of the procedure. To assess VT inducibility in the former experimental group, we applied programmed ventricular stimulation consisting of a pacing train (at either 400 or 500ms cycle length (CL)) followed by the application of up to three extra-stimuli coupled to 190ms. In the case of hESC-CM recipients, overdrive pacing and entrainment of VT were performed from the RV outflow tract at varying CLs. RV pacing was used in P19, 20 and 21, while P20 was also paced at a site close to the focus of earliest activation.

Histological studies

All hearts were fixed with 10% neutral buffered formalin, transversely sectioned at 5 mm intervals on a commercial slicer (Berkel 827A-PLUS, Bonner Springs, KS, USA), processed and paraffin-embedded for histological analyses. Six-micrometer-thick sections were then immunostained using previously reported methods (Laflamme et al., 2007) and primary antibodies directed against cardiac antigens including sarcomeric myosin heavy chain (Developmental Studies Hybridoma Bank (DSHB), Iowa City, IA, USA: cat# MF 20), cardiac troponin T (DSHB: cat# CT3), α-actinin (clone EP2529Y; Abcam, Cambridge, UK: cat# ab68167), titin (DSHB; cat# 9 D10), myosin light chain 2V (Abcam: cat# ab79935), myosin light chain 2A (Sigma-Aldrich, St. Louis, MO, USA: cat# HPA013331), cardiac troponin I (abcam: cat# ab47003), slow skeletal troponin I (Novus Biologicals, Oakville, ON, CA: cat# NBP1-56641), caveolin-3 (Abcam: cat# ab2912), pan-cadherins (Sigma-Aldrich: cat# C3678) and connexin 43 (Abcam: cat# ab11370). Graft cell origin was rigorously confirmed by dual-immunolabeling with a human-specific antibody against the nuclear marker Ku80 (Allard et al., 2014; Weinberger et al., 2016) (Cell Signaling Technology: cat# 2180). To evaluate for the presence of non-cardiac graft elements, we also immunostained with human-specific antibodies against the following cell types: endothelium (CD31/PECAM, Agilent, Santa Clara, CA, USA: cat# M082301-2), epithelium (pan-cytokeratin cocktail AE1/AE3, Agilent: cat# M351501-2), and fibroblasts (clone TE-7, Millipore Sigma, Etobicoke, ON, Canada: cat# CBL271). Host endothelial cells were identified using a species specific antibody against von Willebrand factor (Cloud-Clone Corp., Katy, TX, USA: cat# PAA833Po01), and graft cell proliferation was assessed using a human specific antibody against the nuclear antigen Ki-67 (Agilent, Santa Clara, CA, USA: cat# M724029-2) and phospho-histone H3 (Ser10) (Cell Signaling Technology, Danvers, MA, USA: cat# 9701). H&E, β-tubulin III (Sigma-Aldrich: cat# T8578) and α-1-fetoprotein (Agilent: A0008) were used to detect the presence of any teratomas. CD20 (Biocare Medical, Pacheco, CA, USA: cat#3004), CD3 (Agilent: cat# A0452), and CD45 (Bio-Rad, Mississauga, ON, Canada: cat# MCA1447) were used to detect B-lymphocytes, T-lymphocytes, and pan-leukocytes respectively. Secondary antibodies were either Alexa-conjugated (Thermo Fisher, Waltham, MA, USA: cat# A28175, A27034, A11037, A11032, A28181, A27040) for confocal immunofluorescence or biotinylated (Vector Labs, Burlingame, CA, USA: cat# BA-9200 and BA-1000) for brightfield detection (ABC kit followed by alkaline phosphatase/Vector Red or HRP/DAB, Vector Labs, Burlingame, CA, USA). Scar and graft size was either determined by picrosirius red (Polysciences Inc., Warrington, PA, USA) or aniline blue staining (25g/L in 2% acetic acid) from two whole mount sections originating at 5 and 15mm from apex and quantified using ImageJ software (National Institutes of Health, Bethesda, MD). To suppress autofluorescence seen in tissues analyzed with confocal microscopy, after secondary antibody incubation and washing, the tissue was incubated with 10mM culpric sulfate in 50 mM ammonium acetate buffer (pH 5) for 5 minutes, rinsed with PBS, incubated with Hoechst 33342 Fluorescent Stain (Thermo Fisher, Waltham, MA, USA) and then mounted with ProLong Diamond Antifade Mountant (Thermo Fisher).

Immunofluorescence microscopy was performed using a Zeiss LSM700 inverted confocal microscope with either a FLUAR 10x/0.50 NA, Plan-Apochromat 20x/0.8 NA, Plan-Apochromat 40x/1.4 NA oil immersion, or Plan-Apochromat 63x/1.4 NA oil immersion objective. Images were acquired using the LSM Zen 2012 acquisition software and processed with Corel Draw Graphics Suite 2017.

Electron Microscopy (EM)

Graft from whole mount paraffin embedded blocks from P10 (2-weeks post-transplantation) and P13 (4-weeks post-transplantation) was cored out and processed for EM. Tissue pieces were deparaffinized in xylene, rehydrated in a

series of alcohol and water. The pieces were treated with 2% osmium tetroxide for one hour, rinsed, and dehydrated in alcohol series. A 1:1 mixture of propylene oxide and epon was added for one hour, placed in pure epon solution for 2 hours, embedded in labelled molds and placed in a 70°C oven overnight. One micron sections were cut on glass knives and stained with 1% toluidine blue. Thin sections 60-90nm were cut on a diamond knife (DiATOME, Hatfield, PA, USA) and placed on copper grids. The grids are stained in a saturated solution of aqueous uranyl acetate (2-5%) for one hour at room temperature followed by 12 minutes in lead citrate with a grid stick used for staining. The digital images were taken on a JEOL TEM at 80KV (JEM-1230; JEOL, Peabody, MA, USA).

Statistics

Data are depicted as the mean ± standard error of the mean (SEM). Comparisons were performed using two-tailed Student t-test assuming unequal variance. The significance threshold was set at $p<0.05$.

SUPPLEMENTAL REFERENCES

Allard, J., Li, K., Lopez, X.M., Blanchard, S., Barbot, P., Rorive, S., Decaestecker, C., Pochet, R., Bohl, D., Lepore, A.C., *et al.* (2014). Immunohistochemical toolkit for tracking and quantifying xenotransplanted human stem cells. Regen Med *9*, 437-452.

Cantwell, C.D., Roney, C.H., Ng, F.S., Siggers, J.H., Sherwin, S.J., and Peters, N.S. (2015). Techniques for automated local activation time annotation and conduction velocity estimation in cardiac mapping. Comput Biol Med *65*, 229-242.

Cerqueira, M.D., Weissman, N.J., Dilsizian, V., Jacobs, A.K., Kaul, S., Laskey, W.K., Pennell, D.J., Rumberger, J.A., Ryan, T., Verani, M.S., *et al.* (2002). Standardized myocardial segmentation and nomenclature for tomographic imaging of the heart. A statement for healthcare professionals from the Cardiac Imaging Committee of the Council on Clinical Cardiology of the American Heart Association. Circulation *105*, 539-542.

Chen, V.C., Ye, J., Shukla, P., Hua, G., Chen, D., Lin, Z., Liu, J.C., Chai, J., Gold, J., Wu, J., *et al.* (2015). Development of a scalable suspension culture for cardiac differentiation from human pluripotent stem cells. Stem Cell Res *15*, 365-375.

Chong, J.J., Yang, X., Don, C.W., Minami, E., Liu, Y.W., Weyers, J.J., Mahoney, W.M., Van Biber, B., Palpant, N.J., Gantz, J.A., *et al.* (2014). Human embryonic-stem-cell-derived cardiomyocytes regenerate non-human primate hearts. Nature *510*, 273-277.

Ghugre, N.R., Ramanan, V., Pop, M., Yang, Y., Barry, J., Qiang, B., Connelly, K.A., Dick, A.J., and Wright, G.A. (2011). Quantitative tracking of edema, hemorrhage, and microvascular obstruction in subacute myocardial infarction in a porcine model by MRI. Magn Reson Med *66*, 1129-1141.

Henderson, K.K., Mokelke, E.A., Turk, J.R., Rector, R.S., Laughlin, M.H., and Sturek, M. (2003). Maintaining patency and asepsis of vascular access ports in Yucatan miniature swine. Contemp Top Lab Anim Sci *42*, 28-32.

Laflamme, M.A., Chen, K.Y., Naumova, A.V., Muskheli, V., Fugate, J.A., Dupras, S.K., Reinecke, H., Xu, C., Hassanipour, M., Police, S., *et al.* (2007). Cardiomyocytes derived from human embryonic stem cells in pro-survival factors enhance function of infarcted rat hearts. Nat Biotechnol *25*, 1015-1024.

Laflamme, M.A., Gold, J., Xu, C., Hassanipour, M., Rosler, E., Police, S., Muskheli, V., and Murry, C.E. (2005). Formation of human myocardium in the rat heart from human embryonic stem cells. Am J Pathol *167*, 663-671.

McCall, F.C., Telukuntla, K.S., Karantalis, V., Suncion, V.Y., Heldman, A.W., Mushtaq, M., Williams, A.R., and Hare, J.M. (2012). Myocardial infarction and intramyocardial injection models in swine. Nat Protoc *7*, 1479-1496.

Prowse, A.B., Timmins, N.E., Yau, T.M., Li, R.K., Weisel, R.D., Keller, G., and Zandstra, P.W. (2014). Transforming the promise of pluripotent stem cell-derived cardiomyocytes to a therapy: challenges and solutions for clinical trials. Can J Cardiol *30*, 1335-1349.

Sosa, E., Scanavacca, M., d'Avila, A., and Pilleggi, F. (1996). A new technique to perform epicardial mapping in the electrophysiology laboratory. J Cardiovasc Electrophysiol *7*, 531-536.

Tung, R., Kim, S., Yagishita, D., Vaseghi, M., Ennis, D.B., Ouadah, S., Ajijola, O.A., Bradfield, J.S., Mahapatra, S., Finn, P., *et al.* (2016). Scar voltage threshold determination using ex vivo magnetic resonance imaging integration in

a porcine infarct model: Influence of interelectrode distances and three-dimensional spatial effects of scar. Heart Rhythm *13*, 1993-2002.

Weinberger, F., Breckwoldt, K., Pecha, S., Kelly, A., Geertz, B., Starbatty, J., Yorgan, T., Cheng, K.H., Lessmann, K., Stolen, T., *et al.* (2016). Cardiac repair in guinea pigs with human engineered heart tissue from induced pluripotent stem cells. Sci Transl Med *8*, 363ra148.

Xu, C., Police, S., Hassanipour, M., Li, Y., Chen, Y., Priest, C., O'Sullivan, C., Laflamme, M.A., Zhu, W.Z., Van Biber, B., *et al.* (2011). Efficient generation and cryopreservation of cardiomyocytes derived from human embryonic stem cells. Regenerative Medicine *6*, 53-66.
